# Kolmogorov Capacity with Overlap †

**DOI:** 10.3390/e27050472

**Published:** 2025-04-27

**Authors:** Anshuka Rangi, Massimo Franceschetti

**Affiliations:** Department of Electrical and Computer Engineering, University of California at San Diego, 9500 Gilman Drive, Mail Code 0407, La Jolla, CA 92093-0407, USA; rangianshukaiitr@gmail.com

**Keywords:** *ϵ*-capacity, (*ϵ*,*δ*)-capacity, mutual information, non-stochastic uncertainty

## Abstract

The notion of δ-mutual information between non-stochastic uncertain variables is introduced as a generalization of Nair’s non-stochastic information functional. Several properties of this new quantity are illustrated and used in a communication setting to show that the largest δ-mutual information between received and transmitted codewords over ϵ-noise channels equals the (ϵ,δ)-capacity. This notion of capacity generalizes the Kolmogorov ϵ-capacity to packing sets of overlap at most δ and is a variation of a previous definition proposed by one of the authors. Results are then extended to more general noise models, including non-stochastic, memoryless, and stationary channels. The presented theory admits the possibility of decoding errors, as in classical information theory, while retaining the worst-case, non-stochastic character of Kolmogorov’s approach.

## 1. Introduction

Shannon’s celebrated channel coding theorem states that the capacity is the supremum of the mutual information between the input and the output of the channel [1]. In this setting, the mutual information is intended as the amount of information obtained regarding the random variable at the input of the channel by observing the random variable at the output of the channel, and the capacity is the largest rate of communication that can be achieved with an arbitrarily small probability of error. In an effort to provide an analogous result for safety-critical control systems where occasional decoding errors can result in catastrophic failures, Nair introduced a non-stochastic mutual information functional and established that this equals the zero-error capacity [2], namely the largest rate of communication that can be achieved with zero probability of error. Nair’s approach is based on the calculus of non-stochastic uncertain variables (UVs), and his definition of mutual information in a non-stochastic setting is based on the quantization of the range of uncertainty of a UV induced by the knowledge of the other. While Shannon’s theorem leads to a single letter expression, Nair’s result is multi-letter, involving the non-stochastic information between codeword blocks of *n* symbols. The zero-error capacity can also be formulated as a graph-theoretic property, and the absence of a single-letter expression for general graphs is well known [3,4]. Extensions of Nair’s non-stochastic approach to characterize the zero-error capacity in the presence of feedback from the receiver to the transmitter using nonstochastic directed mutual information have been considered in [5].

Kolmogorov introduced the notion of ϵ-capacity in the context of functional spaces as the logarithm base two of the packing number of the space, namely the logarithm of the maximum number of balls of radius ϵ that can be placed in the space without overlap [6]. Determining this number is analogous to designing a channel codebook such that the distance between any two codewords is at least 2ϵ. In this way, any transmitted codeword that is subject to a perturbation of, at most, ϵ can be recovered at the receiver without error. It follows that the ϵ-capacity per transmitted symbol (*viz.* per signal dimension) corresponds to the zero-error capacity of an additive channel having arbitrary bounded noise of the radius at most ϵ. Lim and Franceschetti extended this concept introducing the (ϵ,δ) capacity [7], defined as the logarithm base two of the largest number of balls of radius ϵ that can be placed in the space with an average codeword overlap of, at most, δ. In this setting, δ measures the amount of error that can be tolerated when designing a codebook in a non-stochastic setting, and the (ϵ,δ) capacity per transmitted symbol corresponds to the largest rate of communication with error at most δ.

The first contribution of this paper is to consider a generalization of Nair’s mutual information based on a quantization of the range of uncertainty of a UV given the knowledge of another, which reduces the uncertainty to, at most, δ, and to show that this new notion corresponds to the (ϵ,δ) capacity. Our definition of (ϵ,δ) capacity is a variation of the one in [7], as it is required to bound the overlap between any pair of balls, rather than the average overlap. For δ=0, we recover Nair’s result for the Kolmogorov ϵ-capacity or, equivalently, for the zero-error capacity of an additive, bounded noise channel. We then extend the results to more general channels where the noise can be different across codewords and is not necessarily contained within a ball of radius ϵ. Finally, we consider the class of non-stochastic, memoryless, stationary uncertain channels, where the noise experienced by a codeword of *n* symbols factorizes into *n* identical terms describing the noise experienced by each codeword symbol. This is the non-stochastic analog of a discrete memoryless channel (DMC), where the current output symbol depends only on the current input symbol, not on any of the previous input symbols, and where the noise distribution is constant across symbol transmissions and differs from Kolmogorov’s ϵ-noise channel, where the noise experienced by one symbol affects the noise experienced by other symbols (in Kolmogorov’s setting, the noise occurs within a ball of radius ϵ. It follows that for any realization where the noise along one dimension (*viz.* symbol) is close to ϵ, the noise experienced by all other symbols lying in the remaining dimensions must be close to zero.). Letting $\left(1-\delta_{n}\right)$ be the confidence of correct decoding after transmitting *n* symbols, we introduce several notions of capacity and establish coding theorems in terms of mutual information for all of them, including a generalization of the zero-error capacity that requires the error sequence $\left\{\delta_{n}\right\}$ to remain constant and a non-stochastic analog of Shannon’s capacity that requires the error sequence to vanish, as in n→∞.

Finally, since in Nair’s case, all of our results are multi-letter, in the Supplementary Materials, we provide some sufficient conditions for the factorization of the mutual information leading to a single-letter expression for the non-stochastic capacity of stationary, memoryless, uncertain channels, provide some examples in which these conditions are satisfied, and compute the corresponding capacity.

The rest of the paper is organized as follows: Section 2 introduces the mathematical framework of non-stochastic uncertain variables that are used throughout the paper. Section 3 introduces the concept of non-stochastic mutual information. Section 4 gives an operational definition of the capacity of a communication channel and relates it to the mutual information. Section 5 extends the results to more general channel models, and Section 6 concentrates on the special case of stationary, memoryless, uncertain channels. Section 7 draws conclusions and discusses future directions.

## 2. Uncertain Variables

We start by reviewing the mathematical framework used in [2] to describe UVs. A UV *X* is a mapping from a sample space Ω to a set X, i.e., for all ω∈Ω, we have x=X(ω)∈X, namely

(1) $$\begin{array}{cc} X: & \;\Omega \to X\\ \; & \;\omega \mapsto X(\omega )=x. \end{array}$$

Given a UV *X*, the marginal range of *X* is

(2) 〚X〛={X(ω):ω∈Ω}.

The joint range of the two UVs *X* and *Y* is

(3) 〚X,Y〛={(X(ω),Y(ω)):ω∈Ω}.

Given a UV *Y*, the conditional range of *X* given Y=y is

(4) 〚X|y〛={X(ω):Y(ω)=y,ω∈Ω},

and the conditional range of *X* given *Y* is

(5) 〚X|Y〛={〚X|y〛:y∈〚Y〛}.

Thus, 〚X|Y〛 denotes the uncertainty in *X*, given the realization of *Y* and that 〚X,Y〛 represents the total joint uncertainty of *X* and *Y*, namely

(6) $$〚X,Y〛=\cup_{y\in 〚Y〛}〚X|y〛\times \left\{y\right\}.$$

Finally, two UVs, *X* and *Y*, are independent if for all x∈〚X〛,

(7) 〚Y|x〛=〚Y〛,

which also implies that for all y∈〚Y〛,

(8) 〚X|y〛=〚X〛.

## 3. δ-Mutual Information

### 3.1. Uncertainty Function

We now introduce a class of functions that are used to express the amount of uncertainty in determining one UV given another. In our setting, an uncertainty function associates a positive number with a given set, which expresses the “massiveness” or “size” of that set.

**Definition 1.**  *Given the set of non-negative real numbers $R_{0}^{+}$ and any set of X, $m_{X}:2^{X}\to R_{0}^{+}$ is an uncertainty function if it is finite and strongly transitive:*

*We have $m_{X}\left(\emptyset \right)=0$, and for all S⊆X,S≠∅,*

(9) $$\begin{array}{c} 0<m_{X}\left(S\right)<\infty . \end{array}$$

*For all $S_{1},S_{2}\subseteq X,$ we have*

(10) $$\max\left\{m_{X}\left(S_{1}\right),m_{X}\left(S_{2}\right)\right\}\leq m_{X}\left(S_{1}\cup S_{2}\right).$$

In the case where X is measurable, an uncertainty function can easily be constructed using a measure. In the case where X is a bounded (not necessarily measurable) metric space and the input set S contains at least two points, an example of an uncertainty function is the diameter.

### 3.2. Association and Dissociation Between UVs

We now introduce notions of association and dissociation between UVs. In the following definitions, we let $m_{X}\left(.\right)$ and $m_{Y}\left(.\right)$ be uncertainity functions defined over sets X and Y corresponding to UVs *X* and *Y*. We use the notation A≻δ to indicate that for all a∈A, we have a>δ. Similarly, we use A⪯δ to indicate that for all a∈A, we have a≤δ, where A⊆R and δ∈R. For A=∅, we assume that A⪯δ is always satisfied, while A≻δ is not. Whenever we consider i≠j, we also assume that $y_{i}\neq y_{j}$ and $x_{i}\neq x_{j}$, where $x_{i},x_{j}\in 〚X〛$ and $y_{i},y_{j}\in 〚Y〛$.

**Definition 2.**  *The sets of association for UVs X and Y are*

(11) $$\begin{array}{cc} A(X;Y) & =\left\{\frac{m_{X}\left(〚X\right|y_{1}〛\cap 〚X\left|y_{2}〛\right)}{m_{X}\left(〚X〛\right)}:y_{1},y_{2}\in 〚Y〛\right\}\setminus \left\{0\right\}, \end{array}$$

(12) $$\begin{array}{cc} A(Y;X) & =\left\{\frac{m_{Y}\left(〚Y\right|x_{1}〛\cap 〚Y\left|x_{2}〛\right)}{m_{Y}\left(〚Y〛\right)}:\;x_{1},x_{2}\in 〚X〛\right\}\setminus \left\{0\right\}. \end{array}$$

The sets of association are used to describe the correlation between two uncertain variables. Since $m_{X}\left(\emptyset \right)=0$, the exclusion of the zero value in (11) and (12) occurs when there is no overlap between the two conditional ranges.

**Definition 3.**  *For any $\delta_{1},\delta_{2}\in \left[0,1\right)$, UVs X and Y are disassociated at levels $\left(\delta_{1},\delta_{2}\right)$ if the following inequalities hold:*

(13) $$A\left(X;Y\right)≻\delta_{1},$$

(14) $$A\left(Y;X\right)≻\delta_{2},$$

*and, in this case, we write $\left(X,Y\right)\overset{d}{↔}\left(\delta_{1},\delta_{2}\right)$.*

**Figure 1:**
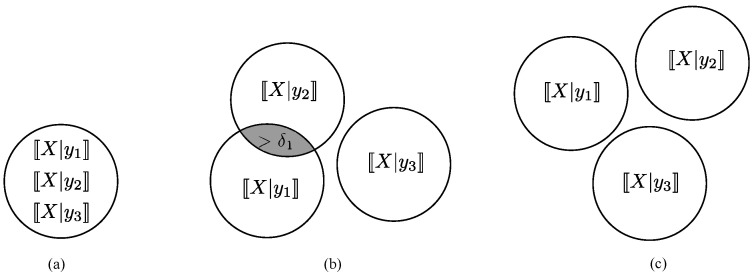
Illustration of disassociation between UVs. Case (**a**): variables are maximally disassociated, and all conditional ranges completely overlap, in that all conditional ranges are equal to 〚X〛 (or 〚Y〛). Case (**b**): variables are disassociated at some levels $\left(\delta_{1},\delta_{2}\right)$, and there is some overlap between at least two conditional ranges. Case (**c**): variables are not disassociated at any levels, and there is no overlap between the conditional ranges.

Having UVs *X* and *Y* be disassociated at levels $\left(\delta_{1},\delta_{2}\right)$ indicates that at least two conditional ranges $〚X|y_{1}〛$ and $〚X|y_{2}〛$ have non-zero overlap and that, given any two conditional ranges, either they do not overlap or the uncertainty associated with their overlap is greater than a $\delta_{1}$ fraction of the total uncertainty associated with 〚X〛; the same holds for conditional ranges $〚Y|x_{1}〛$ and $〚Y|x_{2}〛$ and for level $\delta_{2}$. The levels of disassociation can be viewed as lower bounds in the amount of residual uncertainty in each variable when the other is known. If *X* and *Y* are independent, then all the conditional ranges completely overlap, A(X;Y) and A(Y;X) contain only the element one, and the variables are maximally disassociated (see Figure 1a).

In this case, knowledge of *Y* does not reduce the uncertainty of *X*, and vice versa. On the other hand, when the uncertainty associated with any of the non-zero intersections of the conditional ranges decreases but remains positive, *X* and *Y* become less disassociated in the sense that knowledge of *Y* can reduce the residual uncertainty of *X*, and vice versa (see Figure 1b). When the intersection between every pair of conditional ranges becomes empty, the variables cease to be disassociated (see Figure 1c). Note that excluding the value of 1 in the definition of disassociation allows us to distinguish the case of disassociation from the case of full independence.

An analogous definition of association is given to provide upper bounds on the residual uncertainty of one uncertain variable when the other is known.

**Definition 4.**  *For any $\delta_{1},\delta_{2}\in \left[0,1\right]$, we say that UVs X and Y are associated at levels $\left(\delta_{1},\delta_{2}\right)$ if the following inequalities hold:*

(15) $$A\left(X;Y\right)⪯\delta_{1},$$

(16) $$A\left(Y;X\right)⪯\delta_{2},$$

*and in this case, we write $\left(X,Y\right)\overset{a}{↔}\left(\delta_{1},\delta_{2}\right)$.*

Note that $\delta_{1},\delta_{2}=1$ is included in Definition 4 and not in Definition 3. This is because in Definition 3, we have a strict lower bound on the uncertainty in the association sets.

The following lemma provides the necessary and sufficient conditions for association to hold at given levels. These conditions are stated for all points in the marginal ranges 〚Y〛 and 〚X〛. They show that in the case of association, one can also include in the definition the conditional ranges that have zero intersection. This is not the case for disassociation.

**Lemma 1.**  *For any $\delta_{1},\delta_{2}\in \left[0,1\right]$, $\left(X,Y\right)\overset{a}{↔}\left(\delta_{1},\delta_{2}\right)$ if and only if for all $y_{1},y_{2}\in 〚Y〛$, we have*

(17) $$\frac{m_{X}\left(〚X\right|y_{1}〛\cap 〚X|y_{2}〛\left|\right)}{m_{X}\left(〚X〛\right)}\leq \delta_{1},$$

*and for all $x_{1},x_{2}\in 〚X〛$, we have*

(18) $$\frac{m_{Y}\left(〚Y\right|x_{1}〛\cap 〚Y|x_{2}〛\left|\right)}{m_{Y}\left(〚Y〛\right)}\leq \delta_{2}.$$

**Proof.**  The proof is given in Appendix A. □

An immediate yet important consequence of our definitions is that both association and disassociation at given levels $\left(\delta_{1},\delta_{2}\right)$ cannot hold simultaneously. We also understand that, given any two UVs, one can always select $\delta_{1}$ and $\delta_{2}$ to be large enough such that they are associated at levels $\left(\delta_{1},\delta_{2}\right)$. In contrast, as the smallest value in the sets A(X;Y) and A(Y;X) tends towards zero, the variables eventually cease to be disassociated. Finally, it is possible that two uncertain variables are neither associated nor disassociated at given levels $\left(\delta_{1},\delta_{2}\right)$. Also, any two uncertain variables are associated at level (1,1) trivially by definition.

**Figure 2:**
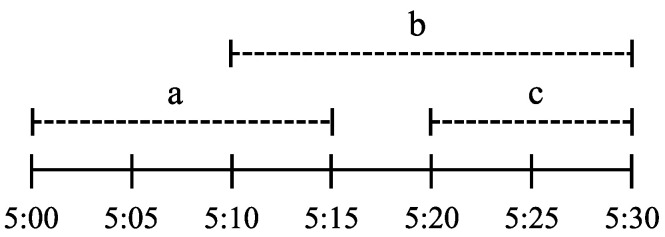
Illustration of the possible time intervals for the walkers on the path.

**Example 1.**  *Consider three individuals, a, b, and c, are going for a walk along a path. Assume they take, at most, 15, 20, and 10 min to finish their walk, respectively. Assume a starts walking at time 5:00, b starts walking at 5:10, and c starts walking at 5:20. Figure 2 shows the possible time intervals for the walkers on the path. Let an uncertain variable W represent the set of walkers that are present on the path at any time and an uncertain variable T represent the time at which any walker on the path finishes their walk. Then, we have the following marginal ranges:*

(19) 〚W〛={{a},{b},{c},{a,b},{b,c}},

(20) 〚T〛=[5:00,5:30].

*We also have the following conditional ranges:*

(21) 〚T|{a}〛=[5:00,5:15],

(22) 〚T|{b}〛=[5:10,5:30],

(23) 〚T|{c}〛=[5:20,5:30],

(24) 〚T|{a,b}〛=[5:10,5:15],

(25) 〚T|{b,c}〛=[5:20,5:30].

*For all t ∈ [5:00, 5:10), we have*

(26) 〚W|t〛={{a}};

*for all t ∈ [5:10, 5:15], we have*

(27) 〚W|t〛={{a,b},{a},{b}};

*for all t ∈ (5:15, 5:20), we have*

(28) 〚W|t〛={{b}};

*and for all t ∈ [5:20, 5:30], we have*

(29) 〚W|t〛={{b,c},{b},{c}}.

*Now, let the uncertainty function of a time set S be*

(30) $$m_{T}\left(S\right)=\left\{\begin{array}{c} L(S)+10\;if\;S\neq \emptyset ,\\ 0\;otherwise, \end{array}\right.$$

*where L(·) is the Lebesgue measure. Let the uncertainty function $m_{W}\left(.\right)$ associated with a set of individuals be the cardinality of the set. Then, the sets of association are*

(31) A(W;T)={1/5,3/5},

(32) A(T;W)={3/8,1/2}.

*It follows that for all $\delta_{1}<1/5$ and $\delta_{2}<3/8$, we have*

(33) $$\left(W,T\right)\overset{d}{↔}\left(\delta_{1},\delta_{2}\right),$$

*and the residual uncertainty in W given T is at least a $\delta_{1}$ fraction of the total uncertainty in W, while the residual uncertainty in T given W is at least a $\delta_{2}$ fraction of the total uncertainty in T. On the other hand, for all $\delta_{1}\geq 3/5$ and $\delta_{2}\geq 1/2$, we have*

(34) $$\left(W,T\right)\overset{a}{↔}\left(\delta_{1},\delta_{2}\right),$$

*and the residual uncertainty in W given T is at most a $\delta_{1}$ fraction of the total uncertainty in W, while the residual uncertainty in T given W is at most a $\delta_{2}$ fraction of the total uncertainty in T.*

*Finally, if $1/5\leq \delta_{1}<3/5$ or $3/8\leq \delta_{2}<1/2$, then W and T are neither associated nor disassociated.*

### 3.3. δ-Mutual Information

We now introduce the mutual information between uncertain variables in terms of some structural properties of covering sets. Intuitively, for any δ∈[0,1] the δ-mutual information, expressed in bits, represents the most refined knowledge that one uncertain variable provides about the other at a given level of confidence (1−δ). We express this idea by considering the quantization of the range of uncertainty of one variable, induced by the knowledge of the other. Such quantization ensures that the variable can be identified with uncertainty at most δ. The notions of association and disassociation introduced above are used to ensure that the mutual information is well defined, in that it can be positive and exhibits a certain symmetric property.

**Definition 5.**  *δ-Connectedness and δ-isolation.*

- *For any δ∈[0,1], points $x_{1},x_{2}\in 〚X〛$ are δ-connected via 〚X|Y〛 and are denoted by $x_{1}\overset{\delta }{↭}x_{2}$ if there exists a finite sequence ${\left\{〚X|y_{i}〛\right\}}_{i=1}^{N}$ of conditional sets such that $x_{1}\in 〚X|y_{1}〛$, $x_{2}\in 〚X|y_{N}〛$ and for all 1<i≤N, we have*
(35) $$\frac{m_{X}\left(〚X\right|y_{i}〛\cap 〚X\left|y_{i-1}〛\right)}{m_{X}\left(〚X〛\right)}>\delta .\;$$
*If $x_{1}\overset{\delta }{↭}x_{2}$ and N=1, then we say that $x_{1}$ and $x_{2}$ are singly δ-connected via 〚X|Y〛, i.e., there exists a y such that $x_{1},x_{2}\in 〚X|y〛$.*
- *A set S⊆〚X〛 is δ-connected via 〚X|Y〛 if every pair of points in the set is δ-connected via 〚X|Y〛.*
- *A set S⊆〚X〛 is singly δ-connected via 〚X|Y〛 if there exists a y∈〚Y〛 such that every point in the set is contained in 〚X|y〛, namely S⊆〚X|y〛.*
- *Two sets $S_{1},S_{2}\subseteq 〚X〛$ are δ-isolated via 〚X|Y〛 if no point in $S_{1}$ is δ-connected to any point in $S_{2}$.*

**Example 2.**  *Consider the same setting discussed in Example 1. For δ=2/8, two points at times 5:05 and 5:25 *∈* *〚*T*〛* are δ-connected. The sequence of conditional sets connecting the two points is {〚T|{a}〛,〚T|{b}〛}, where the sets are defined in (21) and (22). This is because 5:05 *∈* *〚*T*|{*a*}〛*, 5:25 *∈* *〚*T*|{*b*}〛* and *

(36) $$\frac{m_{T}(〚T|\left\{a\right\}〛\cap 〚T|\left\{b\right\}〛)}{m_{T}\left(〚T〛\right)}=\frac{3}{8}>\delta .$$

*For all δ≥0, two points at times 5:00 and 5:05 ∈〚T〛 are singly δ-connected since 5:00, 5:05 ∈〚T|{a}〛.*

*Likewise, for all δ≥0, the set S={〚T|{a}〛} is singly δ-connected by definition.*

*For δ=2/8, the set S={〚T|{a}〛,〚T|{b}〛} is δ-connected. This is because for all $x_{1},x_{2}\in S$, one of the following scenarios holds:*

- *$x_{1},x_{2}\in 〚T|\left\{a\right\}〛$.*
- *$x_{1},x_{2}\in 〚T|\left\{b\right\}〛$.*
- *$x_{1}\in 〚T|\left\{a\right\}〛$ and $x_{2}\in 〚T|\left\{b\right\}〛$, or vice-versa.*

*In the first two scenarios, the points $x_{1}$ and $x_{2}$ are singly δ-connected. In the third scenario, the points are δ-connected since*

(37) $$\frac{m_{T}(〚T|\left\{a\right\}〛\cap 〚T|\left\{b\right\}〛)}{m_{T}\left(〚T〛\right)}=\frac{3}{8}>\delta .$$

*For all δ>0, the two sets 〚T|{a}〛 and 〚T|{c}〛 are δ-isolated since there is no overlap between the two sets.*

**Definition 6.**  *δ-overlap family.*

*For any δ∈[0,1], a 〚X|Y〛 δ-overlap family of 〚X〛, denoted by $〚X|Y{〛}_{\delta }^{*}$, is the largest family of distinct sets covering 〚X〛, such that*

1. *Each set in the family is δ-connected and contains at least one singly δ-connected set of the form 〚X|y〛.*
2. *The measure of overlap between any two distinct sets in the family is at most $\delta m_{X}\left(〚X〛\right)$, namely for all $S_{1},S_{2}\in 〚X|Y{〛}_{\delta }^{*}$, such that $S_{1}\neq S_{2}$; we also have $m_{X}\left(S_{1}\cap S_{2}\right)\leq \delta m_{X}\left(〚X〛\right)$.*
3. *For every singly δ-connected set, there exist a set in the family containing it.*

The first property of the δ-overlap family ensures that points in the same set of the family *cannot* be distinguished with confidence of at least (1−δ), while also ensuring that each set cannot be arbitrarily small. The second and third properties ensure that points that are not covered by the same set of the family *can* be distinguished with confidence of at least (1−δ). It follows that the cardinality of the covering family represents the most refined knowledge at a given level of confidence (1−δ) that we can have about *X*, given the knowledge of *Y*. This also corresponds to the most refined quantization of the set 〚X〛 induced by *Y*. This interpretation is analogous to the one in [2], extending the concept of overlap partition introduced there to a δ-overlap family in this work. The stage is now set to introduce the δ-mutual information in terms of the δ-overlap family.

**Definition 7.**  *The δ-mutual information provided by Y about X is*

(38) $$I_{\delta }\left(X;Y\right)=\log_{2}|〚X|Y{〛}_{\delta }^{*}|bits,$$

*if a 〚X|Y〛δ-overlap family of 〚X〛 exists; otherwise, it is zero.*

We now show that when variables are associated at level $\left(\delta ,\delta_{2}\right)$, there exists a δ-overlap family, so that the mutual information is well defined.

**Theorem 1.**  *If $\left(X,Y\right)\overset{a}{↔}\left(\delta ,\delta_{2}\right)$, then there exists a δ-overlap family $〚X|Y{〛}_{\delta }^{*}$.*

**Proof.**  We show that

(39) 〚X|Y〛={〚X|y〛:y∈〚Y〛}

satisfies all the three properties of δ-overlap family in Definition 6. First, note that 〚X|Y〛 is a cover of 〚X〛, since $〚X〛=\cup_{y\in 〚Y〛}〚X|y〛$, even though 〚X|y〛 for different *y* may overlap with each other. Second, each set in the family 〚X|Y〛 is singly δ-connected via 〚X|Y〛, since trivially, any two points $x_{1},x_{2}\in 〚X|y〛$ are singly δ-connected via the same set. It follows that Property 1 of Definition 6 holds.

Now, since $\left(X,Y\right)\overset{a}{↔}\left(\delta ,\delta_{2}\right)$, then by Lemma 1, for all $y_{1},y_{2}\in 〚Y〛$, we have

(40) $$\frac{m_{X}\left(〚X\right|y_{1}〛\cap 〚X\left|y_{2}〛\right)}{m_{X}\left(〚X〛\right)}\leq \delta ,$$

which shows that Property 2 of Definition 6 holds. Finally, it is also easy to see that Property 3 of Definition 6 holds, since 〚X|Y〛 contains all sets 〚X|y〛. Hence, 〚X|Y〛 satisfies all the three properties in Definition 6, which implies that there exists at least one set satisfying these conditions. Hence, the maximum over these sets is defined and the claim follows. □

Next, we show that a δ-overlap family also exists when variables are disassociated at level $\left(\delta ,\delta_{2}\right)$. In this case, we also characterize the mutual information in terms of a partition of 〚X〛.

**Definition 8.**  *δ-isolated partition.*

*A 〚X|Y〛δ-isolated partition of 〚X〛, denoted by $〚X|Y{〛}_{\delta }$, is a partition of 〚X〛 such that any two sets in the partition are δ-isolated via 〚X|Y〛.*

**Theorem 2.**  *If $\left(X,Y\right)\overset{d}{↔}\left(\delta ,\delta_{2}\right)$, then the following holds:*

*1.*  *There exists a unique δ-overlap family $〚X|Y{〛}_{\delta }^{*}$.*
*2.*  *The δ-overlap family is the δ-isolated partition of largest cardinality, in that, for any $〚X|Y{〛}_{\delta }$, we have*
(41) $$|〚X|Y{〛}_{\delta }|\leq |〚X|Y{〛}_{\delta }^{*}|,$$
*where the equality holds if and only if $〚X|Y{〛}_{\delta }=〚X|Y{〛}_{\delta }^{*}$.*

**Proof.**  First, we show the existence of a δ-overlap family. For all x∈〚X〛, let C(x) be the set of points that are δ-connected to *x* via 〚X|Y〛, namely

(42) $$C\left(x\right)=\left\{x_{1}\in 〚X〛:x\overset{\delta }{↭}x_{1}\right\}.$$

Then, we let

(43) C={C(x):x∈〚X〛},

and show that this is a δ-overlap family. First, note that since $〚X〛=\cup_{S\in C}S$, we know that C is a cover of 〚X〛. Second, for all C(x)∈C, there exists a y∈〚Y〛 such that x∈〚X|y〛, and since any two points $x_{1},x_{2}\in 〚X|y〛$ are singly δ-connected via 〚X|Y〛, we understand that 〚X|y〛⊆C(x). It follows that every set in the family C contains at least one singly δ-connected set. For all $x_{1},x_{2}\in C\left(x\right)$, we also have $x_{1}\overset{\delta }{↭}x$ and $x\overset{\delta }{↭}x_{2}$. Since $\left(X,Y\right)\overset{d}{↔}\left(\delta ,\delta_{2}\right)$, by Lemma A2 in Appendix C, this implies that $x_{1}\overset{\delta }{↭}x_{2}$. It follows that every set in the family C is δ-connected and contains at least one singly δ-connected set, and we conclude that Property 1 of Definition 6 is satisfied.

We now claim that for all $x_{1},x_{2}\in 〚X〛$, if

(44) $$C\left(x_{1}\right)\neq C\left(x_{2}\right),$$

then

(45) $$m_{X}\left(C\left(x_{1}\right)\cap C\left(x_{2}\right)\right)=0.$$

This can be proven by contradiction. Let $C\left(x_{1}\right)\neq C\left(x_{2}\right)$ and assume that $m_{X}\left(C\left(x_{1}\right)\cap C\left(x_{2}\right)\right)\neq 0$. By (9), this implies that $C\left(x_{1}\right)\cap C\left(x_{2}\right)\neq \emptyset$. We can then select $z\in C\left(x_{1}\right)\cap C\left(x_{2}\right)$, such that we have $z\overset{\delta }{↭}x_{1}$ and $z\overset{\delta }{↭}x_{2}$. Since $\left(X,Y\right)\overset{d}{↔}\left(\delta ,\delta_{2}\right)$, by Lemma A2 in Appendix C, this also implies that $x_{1}\overset{\delta }{↭}x_{2}$ , and, therefore, $C\left(x_{1}\right)=C\left(x_{2}\right)$, which is a contradiction. It follows that if $C\left(x_{1}\right)\neq C\left(x_{2}\right)$, then we must have $m_{X}\left(C\left(x_{1}\right)\cap C\left(x_{2}\right)\right)=0$, and, therefore,

(46) $$\frac{m_{X}\left(C\left(x_{1}\right)\cap C\left(x_{2}\right)\right)}{m_{X}\left(〚X〛\right)}=0\leq \delta .$$

We conclude that Property 2 of Definition 6 is satisfied.

Finally, we understand that for any singly δ-connected set 〚X|y〛, there exist an x∈〚X〛 such that x∈〚X|y〛, which by, (42), implies that 〚X|y〛⊆C(x). Namely, for every singly δ-connected set, there exist a set in the family containing it. We can then conclude that C satisfies all the properties of a δ-overlap family.

Next, we show that C is a unique δ-overlap family, which implies that this is also the largest set satisfying the three conditions in Definition 6. By contradiction, consider another δ-overlap family D. For all x∈〚X〛, let D(x) denote a set in D containing *x*. Then, using the definition of C(x) and the fact that D(x) is δ-connected, it follows that

(47) D(x)⊆C(x).

Next, we show that for all x∈〚X〛, we also have

(48) C(x)⊆D(x),

from which, we conclude that D=C.

The proof of (48) is also obtained by contradiction. Assume there exists a point $\tilde{x}\in C\left(x\right)\setminus D\left(x\right)$. Since both *x* and $\tilde{x}$ are contained in C(x), $\tilde{x}\overset{\delta }{↭}x$. Let $x^{*}$ be a point in a singly connected set that is contained in D(x), namely $x^{*}\in 〚X|y^{*}〛\subseteq D\left(x\right)$. Since both *x* and $x^{*}$ are in D(x), we understand that $x\overset{\delta }{↭}x^{*}$. Since $\left(X,Y\right)\overset{d}{↔}\left(\delta ,\delta_{2}\right)$, we can apply Lemma A2 in Appendix C to conclude that $\tilde{x}\overset{\delta }{↭}x^{*}$. It follows that there exists a sequence of conditional ranges ${\left\{〚X|y_{i}〛\right\}}_{i=1}^{N}$ such that $\tilde{x}\in 〚X|y_{1}〛$ and $x^{*}\in 〚X|y_{N}〛$, which satisfies (35). Since $x^{*}$ is in both $〚X|y_{N}〛$ and $〚X|y^{*}〛$, we obtain $〚X|y_{N}〛\cap 〚X|y^{*}〛\neq \emptyset$, and since $\left(X,Y\right)\overset{d}{↔}\left(\delta ,\delta_{2}\right)$, we obtain

(49) $$\frac{m_{X}\left(〚X\right|y_{N}〛\cap 〚X\left|y^{*}〛\right)}{m_{X}\left(〚X〛\right)}>\delta .$$

Without loss of generality, we can then assume that the last element of our sequence is $〚X|y^{*}〛$. By Property 3 of Definition 6, every conditional range in the sequence must be contained in some set of the δ-overlap family D. Since $〚X|y^{*}〛\subseteq D\left(x\right)$ and $〚X|y_{1}〛⊈D\left(x\right)$, it follows that there exist two consecutive conditional ranges along the sequence and two sets of the δ-overlap family covering them, such that $〚X|y_{i-1}〛\subseteq D\left(x_{i-1}\right)$, $〚X|y_{i}〛\subseteq D\left(x_{i}\right)$, and $D\left(x_{i-1}\right)\neq D\left(x_{i}\right)$. Then, we have

(50) $$\begin{array}{cc} \; & \;m_{X}\left(D\left(x_{i-1}\right)\cap D\left(x_{i}\right)\right)\\ = & \;m_{X}\left((〚X\right|y_{i-1}〛\cap 〚X|y_{i}〛)\cup \left(D\left(x_{i-1}^{*}\right)\cap D\left(x_{i}^{*}\right)\right))\\ \overset{\left(a\right)}{\geq } & \;m_{X}\left(〚X\right|y_{i-1}〛\cap 〚X\left|y_{i}〛\right)\\ \overset{\left(b\right)}{>} & \;\delta m_{X}\left(〚X〛\right), \end{array}$$

where (a) follows from (10) and (b) follows from (35). It follows that

(51) $$\frac{m_{X}\left(D\left(x_{i-1}\right)\cap D\left(x_{i}\right)\right)}{m_{X}\left(〚X〛\right)}>\delta ,$$

and Property 2 of Definition 6 is violated. Thus, $\tilde{x}$ does not exists, which implies C(x)⊆D(x). Combining (47) and (48), we conclude that the δ-overlap family C is unique.

We now turn to the proof of the second part of the theorem. Since by (46), the uncertainty associated with the overlap between any two sets of the δ-overlap family C is zero, it follows that C is also a partition.

Now, we show that C is also a δ-isolated partition. This can be proven by contradiction. Assume that C is not a δ-isolated partition. Then, there exists two distinct sets $C\left(x_{1}\right),C\left(x_{2}\right)\in C$ such that $C(x_{1})$ and $C(x_{2})$ are not δ-isolated. This implies that there exists a point ${\bar{x}}_{1}\in C\left(x_{1}\right)$ and ${\bar{x}}_{2}\in C\left(x_{2}\right)$ such that ${\bar{x}}_{1}\overset{\delta }{↭}{\bar{x}}_{2}$. Using the fact that $C(x_{1})$ and $C(x_{2})$ are δ-connected and Lemma A2 in Appendix C, this implies that all points in the set $C(x_{1})$ are δ-connected to all points in the set $C(x_{2})$. Now, let $x_{1}^{*}$ and $x_{2}^{*}$ be points in a singly δ-connected set contained in $C(x_{1})$ and $C(x_{2})$, respectively: $x_{1}^{*}\in 〚X|y_{1}^{*}〛\subseteq C\left(x_{1}\right)$ and $x_{2}^{*}\in 〚X|y_{2}^{*}〛\subseteq C\left(x_{2}\right)$. Since $x_{1}^{*}\overset{\delta }{↭}x_{2}^{*}$, there exists a sequence of conditional ranges ${\left\{〚X|y_{i}〛\right\}}_{i=1}^{N}$ satisfying (35), such that $x_{1}\in 〚X|y_{1}〛$ and $x_{2}\in 〚X|y_{N}〛$. Without loss of generality, we can assume $〚X|y_{1}〛=〚X|y_{1}^{*}〛$ and $〚X|y_{2}〛=〚X|y_{2}^{*}〛$. Since C is a partition, we understand that $〚X|y_{1}^{*}〛\subseteq C\left(x_{1}\right)$ and $〚X|y_{2}^{*}〛⊈C\left(x_{1}\right)$. It follows that there exist two consecutive conditional ranges along the sequence ${\left\{〚X|y_{i}〛\right\}}_{i=1}^{N}$ and two sets of the δ-overlap family C covering them, such that $〚X|y_{i-1}〛\subseteq C\left(x_{i-1}\right)$ and $〚X|y_{i}〛\subseteq C\left(x_{i}\right)$ and that $C\left(x_{i-1}\right)\neq C\left(x_{i}\right)$. Similarly to (50), we hold that

(52) $$\frac{m_{X}\left(C\left(x_{i-1}\right)\cap C\left(x_{i}\right)\right)}{m_{X}\left(〚X〛\right)}>\delta ,$$

and Property 2 of Definition 6 is violated. Thus, $C(x_{1})$ and $C(x_{2})$ do not exist, which implies that C is a δ-isolated partition.

Let P be any other δ-isolated partition. We wish to show that |C|≥|P| and that the equality holds if and only if P=C. First, note that every set C(x)∈C can intersect, at most, one set in P; otherwise, the sets in P would not be δ-isolated. Second, since C is a cover of 〚X〛, every set in P must be intersected by at least one set in C. It follows that

(53) |C|≥|P|.

Now, assume the equality holds. In this case, there is a one-to-one correspondence P:C→P, such that for all x∈〚X〛, we have C(x)⊆P(C(x)), and since both C and P are partitions of 〚X〛, it follows that C=P. Conversely, assuming that C=P, then |C|=|P| follows trivially. □

We have introduced the notion of mutual information from *Y* to *X* in terms of the conditional range 〚X|Y〛. Since, in general, we have 〚X|Y〛≠〚Y|X〛, one may expect the definition of mutual information to be asymmetric in its arguments. Namely, the amount of information provided about *X* by the knowledge of *Y* may not be the same as the amount of information provided about *Y* by the knowledge of *X*. Although this is true in general, we show that for disassociated UVs, symmetry is retained, provided that when swapping *X* with *Y*, one also rescales δ appropriately. The following theorem establishes the symmetry in the mutual information under the appropriate scaling of the parameters $\delta_{1}$ and $\delta_{2}$. The proof requires the introduction of the notions of taxicab connectedness, taxicab family, and taxicab partition, which are given in Appendix C.1, along with the proof of the theorem.

**Theorem 3.**  *If $\left(X,Y\right)\overset{d}{↔}\left(\delta_{1},\delta_{2}\right)$ and a $\left(\delta_{1},\delta_{2}\right)$-taxicab family of 〚X,Y〛 exists, then we have*

(54) $$I_{\delta_{1}}\left(X;Y\right)=I_{\delta_{2}}\left(Y;X\right).$$

## 4. (ϵ,δ)-Capacity

We now give a definition of the capacity of a communication channel and relate it to the notion of mutual information between the UVs introduced above. We consider a normed space X to be totally bounded if, for every ϵ>0, X can be covered by a finite number of open balls of radius ϵ. We let X be a totally bounded, normed space such that for all x∈X, we have ∥x∥≤1, where ∥.∥ represents the norm. This normalization is for convenience in the notation process, and all results can easily be extended to metric spaces of any bounded norm. Let 𝒳⊆X be a discrete set of points in the space, which represents a codebook.

**Definition 9.**  *ϵ-perturbation channel.*

*A channel is called ϵ-perturbation if for any transmitted codeword x∈𝒳, x is received with noise perturbation at most ϵ. Namely, we receive a point in the set*

(55) $$S_{\epsilon }\left(x\right)=\left\{y\in X:∥x-y∥\leq \epsilon \right\}.$$

Given the codebook 𝒳 is transmitted over an ϵ-perturbation channel, all received codewords lie in the set $𝒴={⋃}_{x\in 𝒳}S_{\epsilon }\left(x\right)$, where 𝒴⊆Y=X. Transmitted codewords can be decoded correctly as long as the corresponding uncertainty sets at the receiver do not overlap. This can be achieved by simply associating the received codeword to the point in the codebook that is closest to it.

**Figure 3:**
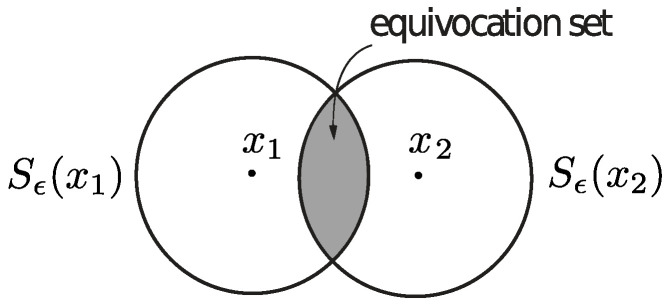
The size of the equivocation set is inversely proportional to the amount of adversarial effort required to induce an error.

For any $x_{1},x_{2}$∈𝒳, we now let

(56) $$e_{\epsilon }\left(x_{1},x_{2}\right)=\frac{m_{Y}\left(S_{\epsilon }\left(x_{1}\right)\cap S_{\epsilon }\left(x_{2}\right)\right)}{m_{Y}\left(Y\right)},$$

where $m_{Y}\left(.\right)$ is an uncertainty function defined over the space Y. We also assume without loss of generality that the uncertainty associated with the whole space Y of received codewords is $m_{Y}\left(Y\right)=1$. Finally, we let $V_{\epsilon }\subseteq Y$ be the smallest uncertainty set corresponding to a transmitted codeword, namely $V_{\epsilon }=S_{\epsilon }\left(x^{*}\right)$, where $x^{*}={\mathrm{argmin}}_{x\in X}m_{Y}\left(S_{\epsilon }\left(x\right)\right)$. The quantity $1-e_{\epsilon }\left(x_{1},x_{2}\right)$ can be viewed as the confidence we have in not confusing $x_{1}$ and $x_{2}$ in any transmission or, equivalently, as the amount of adversarial effort required to induce a confusion between the two codewords. For example, if the uncertainty function is constructed using a measure, then all the erroneous codewords generated by an adversary to decode $x_{2}$ instead of $x_{1}$ must lie inside the equivocation set depicted in Figure 3, whose relative size is given by (56). The smaller the equivocation set is, the larger the effort required by the adversary to induce an error must be. If the uncertainty function represents the diameter of the set, then all the erroneous codewords generated by an adversary to decode $x_{2}$ instead of $x_{1}$ will be close to each other in the sense of (56). Once again, the closer the possible erroneous codewords are, the harder it must be for the adversary to generate an error, since any small deviation allows the decoder to correctly identify the transmitted codeword.

We now introduce the notion of a *distinguishable codebook*, ensuring that every codeword cannot be confused with any other codeword, rather than with a specific one, at a given level of confidence.

**Definition 10.**  *(ϵ,δ)-distinguishable codebook.*

*For any 0<ϵ≤1, $0\leq \delta <m_{Y}\left(V_{\epsilon }\right)$, a codebook 𝒳⊆X is (ϵ,δ)-distinguishable if for all $x_{1},x_{2}\in 𝒳$, we have $e_{\epsilon }\left(x_{1},x_{2}\right)\leq \delta /|𝒳|$.*

For any (ϵ,δ)-distinguishable codebook 𝒳 and x∈𝒳, we let

(57) $$e_{\epsilon }\left(x\right)=\sum_{x^{\prime }\in 𝒳:x^{\prime }\neq x}e_{\epsilon }\left(x,x^{\prime }\right).$$

It now follows from Definition 10 that

(58) $$e_{\epsilon }\left(x\right)\leq \delta ,$$

and each codeword in an (ϵ,δ)-distinguishable codebook can be decoded correctly with confidence of at least 1−δ. Definition 10 guarantees even more, namely that the confidence of not confusing any pair of codewords is uniformly bounded by 1−δ/|𝒳|. This stronger constraint implies that we cannot “balance” the error associated with a codeword transmission by allowing some decoding pair to have a lower confidence and enforcing other pairs to have higher confidence. This is the main difference between our definition and the one used in [7], which bounds the average confidence and allows us to relate the notion of capacity to the mutual information between pairs of codewords.

**Definition 11.**  *(ϵ,δ)-capacity.*

*For any totally bounded, normed metric space X, 0<ϵ≤1, $0\leq \delta <m_{Y}\left(V_{\epsilon }\right)$, and the (ϵ,δ)-capacity of X is*

(59) $$C_{\epsilon }^{\delta }=\underset{𝒳\in X_{\epsilon }^{\delta }}{\sup}\log_{2}\left|𝒳\right|bits,$$

*where $X_{\epsilon }^{\delta }=\left\{𝒳:𝒳is\left(\epsilon ,\delta \right)-distinguishable\right\}$ is the set of (ϵ,δ)-distinguishable codebooks.*

**Figure 4:**
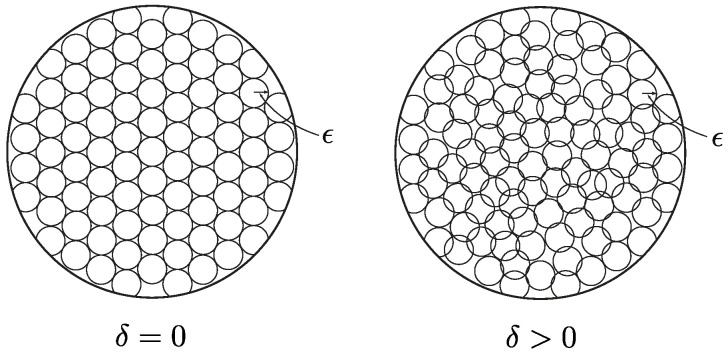
Illustration of the (ϵ,δ)-capacity in terms of packing ϵ-balls with maximum overlap δ.

The (ϵ,δ)-capacity represents the largest number of bits that can be communicated by using any (ϵ,δ)-distinguishable codebook. The corresponding geometric picture is illustrated in Figure 4. For δ=0, our notion of capacity reduces to Kolmogorov’s ϵ-capacity, which is the logarithm of the packing number of the space with balls of radius ϵ.

In the definition of capacity, we have restricted $\delta <m_{Y}\left(V_{\epsilon }\right)$ to rule out cases when the decoding error can be at least as large as the error introduced by the channel and when the (ϵ,δ)-capacity is infinite. Also, note that $m_{Y}\left(V_{\epsilon }\right)\leq 1$ since $V_{\epsilon }\subseteq Y$ and (10) holds.

**Figure 5:**
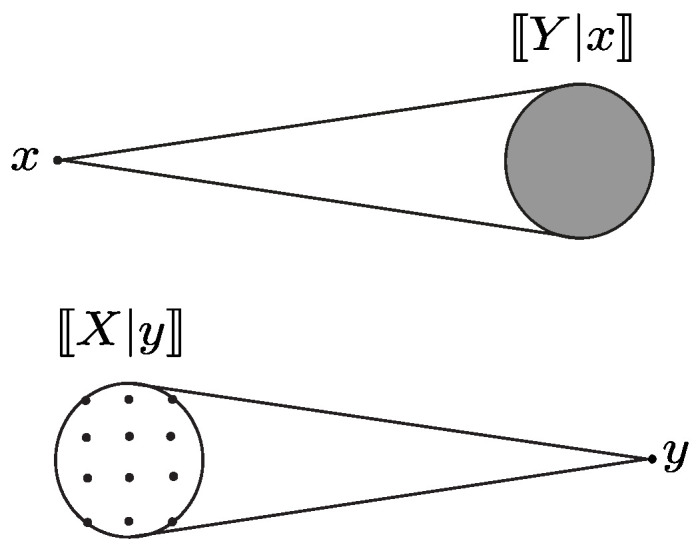
Conditional ranges 〚Y|x〛 and 〚X|y〛 due to the ϵ-perturbation channel.

We now relate our operational definition of capacity to the notion of UVs and mutual information introduced in Section 3. Let *X* be the UV corresponding to the transmitted codeword. This is a map X:X→𝒳 and 〚X〛=𝒳⊆X. Likewise, let *Y* be the UV corresponding to the received codeword. This is a map Y:Y→𝒴 and 〚Y〛=𝒴⊆Y. For our ϵ-perturbation channel, these UVs are such that for all y∈〚Y〛 and x∈〚X〛, we have

(60) $$\begin{array}{cc} 〚Y|x〛 & =\{y\in 〚Y〛:∥x-y∥\leq \epsilon \}, \end{array}$$

(61) $$\begin{array}{cc} 〚X|y〛 & =\{x\in 〚X〛:∥x-y∥\leq \epsilon \}, \end{array}$$

(see Figure 5). Clearly, the set in (60) is continuous, while the set in (61) is discrete.

To measure the levels of association and disassociation between *X* and *Y*, we use an uncertainty function $m_{X}\left(.\right)$ defined over X and $m_{Y}\left(.\right)$ defined over Y. We introduce the feasible set

(62) $$\begin{array}{cc} F_{\delta } & =\{X:〚X〛\subseteq X,\;\mathrm{and}\;\mathrm{either}\\ \; & \;\left(X,Y\right)\overset{d}{↔}\left(0,\delta /|〚X〛|\right)\\ \; & \;\mathrm{or}\left(X,Y\right)\overset{a}{↔}(1,\delta /|〚X〛|)\}, \end{array}$$

representing the set of UVs *X* such that the marginal range 〚X〛 is a discrete set representing a codebook, and the UV can either achieve (0,δ/|〚X〛|) levels of disassociation or (1,δ/|〚X〛|) levels of association with *Y*. In our channel model, this feasible set also depends on the ϵ-perturbation through (60) and (61).

We can now state the non-stochastic channel coding theorem for our ϵ-perturbation channel.

**Theorem 4.**  *For any totally bounded, normed metric space X, ϵ-perturbation channel satisfying (60) and (61), 0<ϵ≤1, and $0\leq \delta <m_{Y}\left(V_{\epsilon }\right)$, we have*

(63) $$C_{\epsilon }^{\delta }=\underset{X\in F_{\tilde{\delta }},\tilde{\delta }\leq \delta /m_{Y}\left(〚Y〛\right)}{\sup}I_{\tilde{\delta }/\left|〚X〛\right|}\left(Y;X\right)bits.$$

**Proof.**  First, we show that there exists a UV *X* and $\tilde{\delta }\leq \delta /m_{Y}\left(〚Y〛\right)$ such that $X\in F_{\tilde{\delta }}$, which implies that the supremum is well defined. Second, for all *X* and $\tilde{\delta }$ such that

(64) $$X\in F_{\tilde{\delta }},$$

and

(65) $$\tilde{\delta }\leq \delta /m_{Y}\left(〚Y〛\right),$$

we show that

(66) $$I_{\tilde{\delta }/\left|〚X〛\right|}\left(Y;X\right)\leq C_{\epsilon }^{\delta }.$$

Finally, we show the existence of $X\in F_{\tilde{\delta }}$ and $\tilde{\delta }\leq \delta /m_{Y}\left(〚Y〛\right)$ such that $I_{\tilde{\delta }/\left|〚X〛\right|}\left(Y;X\right)=C_{\epsilon }^{\delta }$.

Let us begin with the first step. Consider a point x∈X. Let *X* be a UV such that

(67) 〚X〛={x}.

Then, we hold that the marginal range of the UV *Y* corresponding to the received variable is

(68) 〚Y〛=〚Y|x〛,

and, therefore, for all y∈〚Y〛, we have

(69) 〚X|y〛={x}.

Using Definition 2 and (67), we hold that

(70) A(Y;X)=∅,

because 〚X〛 consists of a single point, and, therefore, the set in (12) is empty.

On the other hand, using Definition 2 and (69), we have

(71) $$A\left(X;Y\right)=\left\{\begin{array}{c} \left\{1\right\}\;\mathrm{if}\;\exists y_{1},y_{2}\in 〚Y〛,\\ \emptyset \;\mathrm{otherwise}. \end{array}\right.$$

Using (70), and since A⪯δ holds for A=∅, we have

(72) $$A\left(Y;X\right)⪯\delta /(|〚X〛|m_{Y}\left(〚Y〛\right)).$$

Similarly, using (71), we have

(73) A(X;Y)⪯1.

Now, combining (72) and (73), we have

(74) $$\left(X,Y\right)\overset{a}{↔}\left(1,\delta /(|〚X〛\right|m_{Y}\left(〚Y〛\right))).$$

Letting $\tilde{\delta }=\delta /m_{Y}\left(〚Y〛\right)$, this implies that $X\in F_{\tilde{\delta }}$ and the first step of the proof is complete.

To prove the second step, we define the set of discrete UVs

(75) $$\begin{array}{cc} G & =\{X:〚X〛\subseteq X,\exists \tilde{\delta }\leq \delta /m_{Y}\left(〚Y〛\right)\\ \; & \;\;\mathrm{such}\;\mathrm{that}\;\forall S_{1},S_{2}\in 〚Y|X〛,\\ \; & \;\;m_{Y}\left(S_{1}\cap S_{2}\right)/m_{Y}\left(〚Y〛\right)\leq \tilde{\delta }/|〚X〛|\}, \end{array}$$

which is a larger set than the one containing all UVs *X* that are $\left(1,\tilde{\delta }/|〚X〛|\right)$ associated with *Y*. Now, we will show that if a UV X∈G, then the corresponding codebook $𝒳\in X_{\epsilon }^{\delta }$. If X∈G, then there exists a $\tilde{\delta }\leq \delta /m_{Y}\left(〚Y〛\right)$ such that for all $S_{1},S_{2}\in 〚Y|X〛$, we have

(76) $$\frac{m_{Y}\left(S_{1}\cap S_{2}\right)}{m_{Y}\left(〚Y〛\right)}\leq \frac{\tilde{\delta }}{\left|〚X〛\right|}.$$

It follows that for all $x_{1},x_{2}\in 〚X〛$, we have

(77) $$\frac{m_{Y}\left(〚Y\right|x_{1}〛\cap 〚Y\left|x_{2}〛\right)}{m_{Y}\left(〚Y〛\right)}\leq \frac{\tilde{\delta }}{\left|〚X〛\right|}.$$

Using 𝒳=〚X〛, (60), $〚Y〛=𝒴={⋃}_{x\in 𝒳}S_{\epsilon }\left(x\right)$ and $m_{Y}\left(Y\right)=1$, for all $x_{1},x_{2}\in 𝒳$, we have

(78) $$\begin{array}{cc} \frac{m_{Y}\left(S_{\epsilon }\left(x_{1}\right)\cap S_{\epsilon }\left(x_{2}\right)\right)}{m_{Y}\left(Y\right)} & \leq \frac{\tilde{\delta }m_{Y}\left(〚Y〛\right)}{\left|𝒳\right|},\\ \; & \overset{\left(a\right)}{\leq }\frac{\delta }{\left|𝒳\right|}, \end{array}$$

where (a) follows from $\tilde{\delta }\leq \delta /m_{Y}\left(〚Y〛\right)$. Putting things together, it follows that

(79) $$X\in G\Rightarrow 𝒳\in X_{\epsilon }^{\delta }$$

Consider now a pair of *X* and $\tilde{\delta }$ such that $\tilde{\delta }\leq \delta /m_{Y}\left(〚Y〛\right)$ and

(80) $$X\in F_{\tilde{\delta }}.$$

If $\left(X,Y\right)\overset{d}{↔}(0,\tilde{\delta }/\left|〚X〛|\right)$, then, using Lemma A1 in Appendix C, there exist two UVs, $\bar{X}$ and $\bar{Y}$ and $\bar{\delta }\leq \delta /m_{Y}\left(〚\bar{Y}〛\right)$, such that

(81) $$\left(\bar{X},\bar{Y}\right)\overset{a}{↔}(1,\bar{\delta }/|〚\bar{X}〛\left|\right),$$

and

(82) $$|〚Y|X{〛}_{\tilde{\delta }/\left|〚X〛\right|}^{*}|=|〚\bar{Y}\left|\bar{X}{〛}_{\bar{\delta }/\left|〚\bar{X}〛\right|}^{*}\right|.$$

On the other hand, if $\left(X,Y\right)\overset{a}{↔}(1,\tilde{\delta }/\left|〚X〛|\right)$, then (81) and (82) also trivially hold. It then follows that (81) and (82) hold for all $X\in F_{\tilde{\delta }}$. We now have

(83) $$\begin{array}{cc} I_{\tilde{\delta }/\left|〚X〛\right|}\left(Y;X\right) & =\log(|〚Y|X{〛}_{\tilde{\delta }/\left|〚X〛\right|}^{*}|)\\ \; & \overset{\left(a\right)}{=}\log(|〚\bar{Y}|\bar{X}{〛}_{\bar{\delta }/\left|〚\bar{X}〛\right|}^{*}\left|\right)\\ \; & \overset{\left(b\right)}{\leq }\log(|〚\bar{X}〛\left|\right),\\ \; & \overset{\left(c\right)}{=}\log(|\bar{𝒳}\left|\right),\\ \; & \overset{\left(d\right)}{\leq }C_{\epsilon }^{\delta }, \end{array}$$

where (a) follows from (81) and (82), (b) follows from Lemma A3 in Appendix C since $\bar{\delta }\leq \delta /m_{Y}\left(〚\bar{Y}〛\right)<m_{Y}\left(V_{\epsilon }\right)/m_{Y}\left(〚\bar{Y}〛\right)$, (c) follows by defining the codebook $\bar{𝒳}$ corresponding to the UV $\bar{X}$, and (d) follows from the fact that using (81) and Lemma 1 allows $\bar{X}\in G$, which implies for (79) that $\bar{𝒳}\in X_{\epsilon }^{\delta }$.

Finally, let

(84) $$X^{*}={argsup}_{𝒳\in X_{\epsilon }^{\delta }}\log\left(|𝒳|\right),$$

which achieves the capacity $C_{\epsilon }^{\delta }$. Let $X^{*}$ be the UV whose marginal range corresponds to the codebook $X^{*}$. It follows that for all $S_{1},S_{1}\in 〚Y^{*}|X^{*}〛$, we have

(85) $$\frac{m_{Y}\left(S_{1}\cap S_{1}\right)}{m_{Y}\left(Y\right)}\leq \frac{\delta }{|〚X^{*}〛|},$$

which implies that $m_{Y}\left(Y\right)=1$,

(86) $$\frac{m_{Y}\left(S_{1}\cap S_{1}\right)}{m_{Y}\left(〚Y^{*}〛\right)}\leq \frac{\delta }{|〚X^{*}〛|m_{Y}\left(〚Y^{*}〛\right)}.$$

Letting $\delta^{*}=\delta /m_{Y}\left(〚Y^{*}〛\right)$, and using Lemma 1, we hold that $\left(X^{*},Y^{*}\right)\overset{a}{↔}(1,\delta^{*}/|〚X^{*}〛\left|\right)$, which implies that $X^{*}\in \cup_{\tilde{\delta }\leq \delta /m_{Y}(〚Y^{*}〛}F_{\tilde{\delta }}$, and the proof is complete. □

Theorem 4 characterizes the capacity as the supremum of the mutual information over all UVs in the feasible set. The following theorem shows that the same characterization is obtained if we optimize the right-hand side in (63) over all UVs in the space. It follows by Theorem 4 that rather than optimizing over all UVs representing all the codebooks in the space, a capacity-achieving codebook can be found within the smaller class $\cup_{\tilde{\delta }\leq \delta /m_{Y}\left(V_{\epsilon }\right)}F_{\tilde{\delta }}$ of feasible sets with error at most $\delta /m_{Y}\left(V_{\epsilon }\right)$, since for all 〚Y〛⊆Y, $m_{Y}\left(V_{\epsilon }\right)\leq m_{Y}\left(〚Y〛\right)$.

**Theorem 5.**  *The (ϵ,δ)-capacity in (63) can also be written as*

(87) $$C_{\epsilon }^{\delta }=\underset{\begin{array}{c} X:〚X〛\subseteq X,\\ \tilde{\delta }\leq \delta /m_{Y}\left(〚Y〛\right) \end{array}}{\sup}I_{\tilde{\delta }/\left|〚X〛\right|}\left(Y;X\right)bits.$$

**Proof.**  Consider a UV $X\notin \cup_{\tilde{\delta }\leq \delta /m_{Y}\left(〚Y〛\right)}F_{\tilde{\delta }}$, where *Y* is the corresponding UV at the receiver. The idea of the proof is to show the existence of a UV $\bar{X}\in \cup_{\tilde{\delta }\leq \delta /m_{Y}\left(〚\bar{Y}〛\right)}F_{\tilde{\delta }}$ and the corresponding UV $\bar{Y}$ at the receiver, and

(88) $$\bar{\delta }=\tilde{\delta }m_{Y}\left(〚Y〛\right)/m_{Y}\left(〚\bar{Y}〛\right)\leq \delta /m\left(〚\bar{Y}〛\right),$$

such that the cardinality of the overlap partitions

(89) $$|〚\bar{Y}|\bar{X}{〛}_{\bar{\delta }/\left|〚\bar{X}〛\right|}^{*}|=|〚Y|X{〛}_{\tilde{\delta }/\left|〚X〛\right|}^{*}|.$$

Let the cardinality

(90) $$|〚Y|X{〛}_{\tilde{\delta }/\left|〚X〛\right|}^{*}|=K.$$

By Property 1 of Definition 6, we hold that for all $S_{i}\in 〚Y|X{〛}_{\tilde{\delta }/\left|〚X〛\right|}^{*}$, there exists an $x_{i}\in 〚X〛$ such that $〚Y|x_{i}〛\subseteq S_{i}$. Now, consider another UV $\bar{X}$ whose marginal range is composed of *K* elements of 〚X〛, namely

(91) $$〚\bar{X}〛=\left\{x_{1},\ldots x_{K}\right\}.$$

Let $\bar{Y}$ be the UV corresponding to the received variable. Using the fact that for all x∈X, we have $〚\bar{Y}\left|x〛=〚Y\right|x〛$ since (60) holds, and using Property 2 of Definition 6, for all $x,x^{\prime }\in 〚\bar{X}〛$, we obtain

(92) $$\begin{array}{cc} \frac{m_{Y}(〚\bar{Y}|x〛\cap 〚\bar{Y}\left|x^{\prime }〛\right)}{m_{Y}\left(〚Y〛\right)} & \leq \frac{\tilde{\delta }}{\left|〚X〛\right|},\\ \; & \overset{\left(a\right)}{\leq }\frac{\tilde{\delta }}{|〚\bar{X}〛|}, \end{array}$$

where (a) follows from the fact that $〚\bar{X}〛\subseteq 〚X〛$ using (91). Then, for all $x,x^{\prime }\in 〚\bar{X}〛$, we hold that

(93) $$\begin{array}{cc} \frac{m_{Y}(〚\bar{Y}|x〛\cap 〚\bar{Y}\left|x^{\prime }〛\right)}{m_{Y}\left(〚\bar{Y}〛\right)} & \leq \frac{\tilde{\delta }m_{Y}\left(〚Y〛\right)}{|〚\bar{X}〛|m_{Y}\left(〚\bar{Y}〛\right)}\\ \; & =\frac{\bar{\delta }}{|〚\bar{X}〛|}, \end{array}$$

since $\bar{\delta }=\tilde{\delta }m_{Y}\left(〚Y〛\right)/m_{Y}\left(〚\bar{Y}〛\right)$. Then, by Lemma 1, it follows that

(94) $$\left(\bar{X},\bar{Y}\right)\overset{a}{↔}(1,\bar{\delta }/|〚\bar{X}〛\left|\right).$$

Since $\tilde{\delta }\leq \delta /m_{Y}\left(〚Y〛\right)$, we have

(95) $$\bar{\delta }\leq \delta /m_{Y}\left(〚\bar{Y}〛\right)<m_{Y}\left(V_{\epsilon }\right)/m_{Y}\left(〚\bar{Y}〛\right).$$

Therefore, $\bar{X}\in F_{\bar{\delta }}$ and $\bar{\delta }\leq \delta /m_{Y}\left(〚\bar{Y}〛\right)$. We now hold that

(96) $$\begin{array}{cc} |〚\bar{Y}\left|\bar{X}{〛}_{\bar{\delta }/\left|〚\bar{X}〛\right|}^{*}\right| & \overset{\left(a\right)}{=}\left|〚\bar{X}〛\right|\\ \; & \overset{\left(b\right)}{=}|〚Y|X{〛}_{\tilde{\delta }/\left|〚X〛\right|}^{*}|, \end{array}$$

where (a) follows by applying Lemma A4 in Appendix C using (94) and (95) and (b) follows from (90) and (91). Combining (96) with Theorem 4, the proof is complete. □

We now make some considerations with respect to previous results in the literature. First, we note that for δ=0, all of our definitions reduce to Nair’s ones and Theorem 4 recovers Nair’s coding theorem ([2] (Theorem 4.1)) for the zero-error capacity of an additive ϵ-perturbation channel.

Second, we point out that the (ϵ,δ)-capacity considered in [7] defines the set of (ϵ,δ)-distinguishable codewords such that the *average* overlap among all codewords is at most δ. In contrast, our definition requires the overlap for *each* pair of codewords to be at most δ/|𝒳|. The following theorem provides the relationship between our $C_{\epsilon }^{\delta }$ and the capacity ${\tilde{C}}_{\epsilon }^{\delta }$ considered in [7], which is defined using the Euclidean norm.

**Theorem 6.**  *Let ${\tilde{C}}_{\epsilon }^{\delta }$ be the (ϵ,δ)-capacity defined in [7]. We have*

(97) $$C_{\epsilon }^{\delta }\leq {\tilde{C}}_{\epsilon }^{\delta /(2m_{Y}\left(V_{\epsilon }\right))},$$

*and*

(98) $${\tilde{C}}_{\epsilon }^{\delta }\leq C_{\epsilon }^{\delta m_{Y}\left(V_{\epsilon }\right)2^{2{\tilde{C}}_{\epsilon }^{\delta }+1}}.$$

**Proof.**  For every codebook $𝒳\in X_{\epsilon }^{\delta }$ and $x_{1},x_{2}\in 𝒳$, we have

(99) $$e_{\epsilon }\left(x_{1},x_{2}\right)\leq \delta /\left|𝒳\right|.$$

Since $m_{Y}\left(Y\right)=1$, this implies that for all $x_{1},x_{2}\in 𝒳$, we have

(100) $$\begin{array}{cc} m_{Y}\left(S_{\epsilon }\left(x_{1}\right)\cap S_{\epsilon }\left(x_{2}\right)\right) & \leq \delta /|𝒳|. \end{array}$$

For all 𝒳∈X, the average overlap defined in ([7] (53)) is

(101) $$\Delta =\frac{1}{\left|𝒳\right|}\sum_{x\in 𝒳}\frac{e_{\epsilon }\left(x\right)}{2m_{Y}\left(V_{\epsilon }\right)}.$$

Then, we have

(102) $$\begin{array}{cc} \Delta & =\frac{1}{2|𝒳|m_{Y}\left(V_{\epsilon }\right)}\sum_{x_{1},x_{2}\in 𝒳}m_{Y}\left(S_{\epsilon }\left(x_{1}\right)\cap S_{\epsilon }\left(x_{2}\right)\right),\\ \; & \overset{\left(a\right)}{\leq }\frac{{\delta |𝒳|}^{2}}{{2|𝒳|}^{2}m_{Y}\left(V_{\epsilon }\right)},\\ \; & \leq \frac{\delta }{2m_{Y}\left(V_{\epsilon }\right)}, \end{array}$$

where (a) follows from (100). Thus, we have

(103) $$C_{\epsilon }^{\delta }\leq {\tilde{C}}_{\epsilon }^{\delta /(2m_{Y}\left(V_{\epsilon }\right))},$$

and (97) follows.

Now, let 𝒳 be a codebook with average overlap at most δ, namely

(104) $$\frac{1}{2|𝒳|m_{Y}\left(V_{\epsilon }\right)}\sum_{x_{1},x_{2}\in 𝒳}m_{Y}\left(S_{\epsilon }\left(x_{1}\right)\cap S_{\epsilon }\left(x_{2}\right)\right)\leq \delta .$$

This implies that for all $x_{1},x_{2}\in 𝒳$, we have

(105) $$\begin{array}{cc} \frac{\left|𝒳\right|m_{Y}\left(S_{\epsilon }\left(x_{1}\right)\cap S_{\epsilon }\left(x_{2}\right)\right)}{m_{Y}\left(Y\right)} & \leq \frac{{2\delta |𝒳|}^{2}m_{Y}\left(V_{\epsilon }\right)}{m_{Y}\left(Y\right)},\\ \; & \overset{\left(a\right)}{=}{2\delta |𝒳|}^{2}m_{Y}\left(V_{\epsilon }\right),\\ \; & \leq \delta 2^{2{\tilde{C}}_{\epsilon }^{\delta }+1}m_{Y}\left(V_{\epsilon }\right), \end{array}$$

where (a) follows from the fact that $m_{Y}\left(Y\right)=1$. Thus, we have

(106) $${\tilde{C}}_{\epsilon }^{\delta }\leq C_{\epsilon }^{\delta 2^{2{\tilde{C}}_{\epsilon }^{\delta }+1}m_{Y}\left(V_{\epsilon }\right)},$$

and (98) follows. □

**Figure 6:**
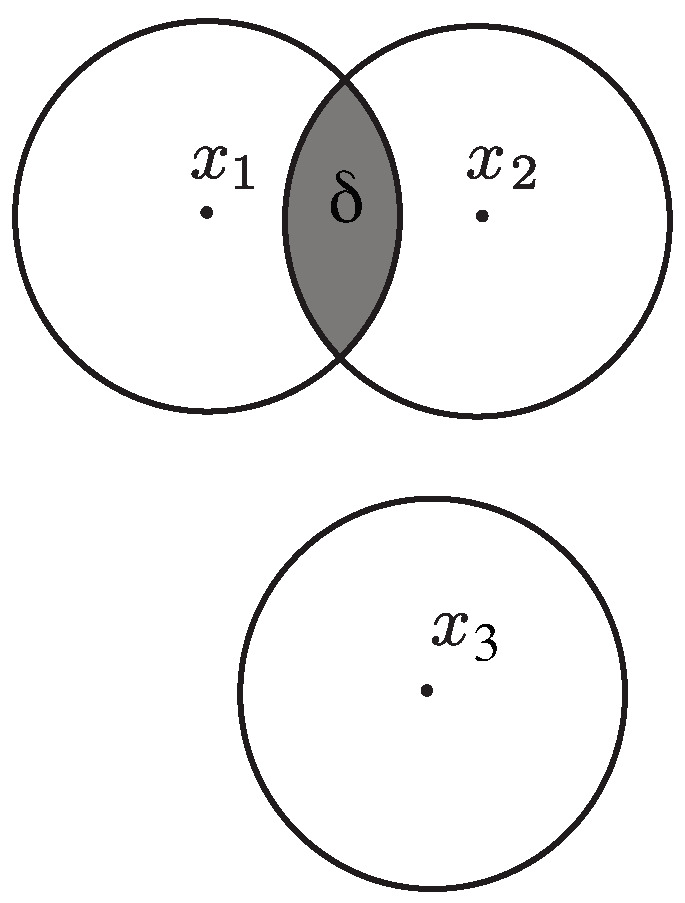
Output configuration for the computation of $C_{\epsilon }^{\delta^{\prime }}$ and ${\tilde{C}}_{\epsilon }^{\delta^{\prime }}$.

To better understand the relationship between the two capacities and show how they can be distinct, consider the case in which the output space is the union of the three ϵ-balls depicted in Figure 6; this is the only feasible output configuration.

We now compute the two capacities $C_{\epsilon }^{\delta^{\prime }}$ and ${\tilde{C}}_{\epsilon }^{\delta^{\prime }}$ in this case. We have

(107) $$m_{Y}\left(S_{\epsilon }\left(x_{1}\right)\cap S_{\epsilon }\left(x_{2}\right)\right)=\delta ,$$

and the average overlap (101) is

(108) $$\Delta =\frac{1}{3}\frac{\delta }{2}=\frac{\delta }{6}.$$

It follows that

(109) $${\tilde{C}}_{\epsilon }^{\delta^{\prime }}=\left\{\begin{array}{cc} \log_{2}3, & \mathrm{if}\;\delta^{\prime }\geq \delta /6.\\ \log_{2}2, & \mathrm{otherwise}. \end{array}\right.$$

On the other hand, the worst case overlap is

(110) $$m_{Y}\left(S_{\epsilon }\left(x_{1}\right)\cap S_{\epsilon }\left(x_{2}\right)\right)=\delta =\frac{3\delta }{\left|X\right|},$$

and it follows that

(111) $$C_{\epsilon }^{\delta^{\prime }}=\left\{\begin{array}{cc} \log_{2}3, & \mathrm{if}\;\delta^{\prime }\geq 3\delta .\\ \log_{2}2, & \mathrm{otherwise}. \end{array}\right.$$

## 5. (N,δ)-Capacity of General Channels

We now extend our results to more general channels where the noise can be different across codewords and is not necessarily contained within a ball of radius ϵ.

Let 𝒳⊆X be a discrete set of points in the space, which represents a codebook. Any point x∈𝒳 represents a codeword that can be selected at the transmitter, sent over the channel, and received with perturbation. A channel with transition mapping N:X→Y associates with any point in X a set in Y, such that the received codeword lies in the set

(112) $$S_{N}\left(x\right)=\left\{y\in Y:y\in N\left(x\right)\right\}.$$

**Figure 7:**
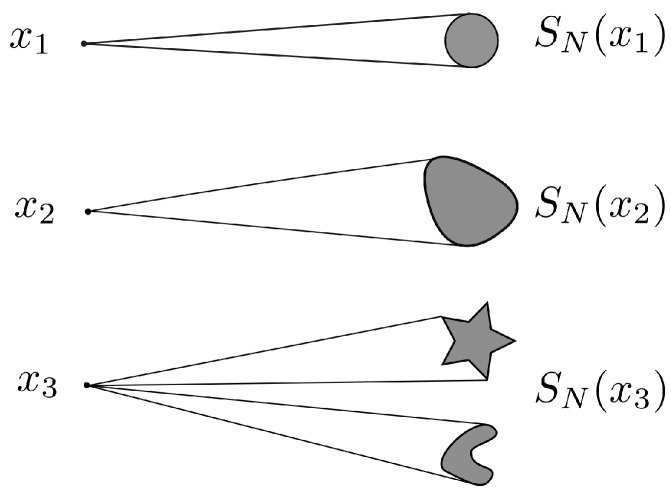
Uncertainty sets associated with three different codewords. Sets are not necessarily balls; they can be different across codewords and can also be composed of disconnected subsets.

Figure 7 illustrates possible uncertainty sets associated with three different codewords.

All received codewords lie in the set $𝒴={⋃}_{x\in 𝒳}S_{N}\left(x\right)$, where 𝒴⊆Y. For any $x_{1},x_{2}$∈X, we now let

(113) $$e_{N}\left(x_{1},x_{2}\right)=\frac{m_{Y}\left(S_{N}\left(x_{1}\right)\cap S_{N}\left(x_{2}\right)\right)}{m_{Y}\left(Y\right)},$$

where $m_{Y}\left(.\right)$ is an uncertainty function defined over Y. We also assume without loss of generality that the uncertainty associated with the space Y of received codewords is $m_{Y}\left(Y\right)=1$. We also let $V_{N}=N\left(x^{*}\right)$, where $x^{*}={\mathrm{argmin}}_{x\in X}m_{Y}\left(N\left(x\right)\right)$. Thus, $V_{N}$ is the set corresponding to the minimum uncertainty introduced by the noise mapping *N*.

**Definition 12.**  *(N,δ)-distinguishable codebook.*

*For any $0\leq \delta <m_{Y}\left(V_{N}\right)$, a codebook 𝒳⊆X is (N,δ)-distinguishable if for all $x_{1},x_{2}\in 𝒳$, we have $e_{N}\left(x_{1},x_{2}\right)\leq \delta /\left|𝒳\right|$.*

**Definition 13.**  *(N,δ)-capacity.*

*For any totally bounded, normed metric space X, channel with transition mapping N, and $0\leq \delta <m_{Y}\left(V_{N}\right)$, the (N,δ)-capacity of X is*

(114) $$C_{N}^{\delta }=\underset{𝒳\in X_{N}^{\delta }}{\sup}\log_{2}\left|𝒳\right|bits,$$

*where $X_{N}^{\delta }=\left\{𝒳:𝒳is\left(N,\delta \right)-distinguishable\right\}$.*

We now relate our definition of capacity to the notion of UVs and mutual information introduced in Section 3. As usual, let *X* be the UV corresponding to the transmitted codeword and *Y* be the UV corresponding to the received codeword. For a channel with transition mapping *N*, these UVs are such that for all y∈〚Y〛 and x∈〚X〛, we have

(115) $$\begin{array}{cc} 〚Y|x〛 & =\{y\in 〚Y〛:y\in N(x)\}, \end{array}$$

(116) $$\begin{array}{cc} 〚X|y〛 & =\{x\in 〚X〛:y\in N(x)\}. \end{array}$$

To measure the levels of association and disassociation between UVs *X* and *Y*, we use an uncertainty function $m_{X}\left(.\right)$ defined over X, and $m_{Y}\left(.\right)$ is defined over Y. The definition of the feasible set is the same as the one given in (62). In our channel model, this feasible set depends on the transition mapping *N* through (115) and (116).

We can now state the non-stochastic channel coding theorem for channels with transition mapping *N*.

**Theorem 7.**  *For any totally bounded, normed metric space X, channel with transition mapping N satisfying (115) and (116), and $0\leq \delta <m_{Y}\left(V_{N}\right)$, we have*

(117) $$C_{N}^{\delta }=\underset{X\in F_{\tilde{\delta }},\tilde{\delta }\leq \delta /m_{Y}\left(〚Y〛\right)}{\sup}I_{\tilde{\delta }/\left|〚X〛\right|}\left(Y;X\right)\;bits.$$

The proof is along the same lines as the one of Theorem 4 and is omitted.

Theorem 7 characterizes the capacity as the supremum of the mutual information over all codebooks in the feasible set. The following theorem shows that the same characterization is obtained if we optimize the right hand side in (117) over all codebooks in the space. It follows by Theorem 7 that rather than optimizing over all codebooks, a capacity-achieving codebook can be found within the smaller class $\cup_{\tilde{\delta }\leq \delta /m_{Y}\left(V_{N}\right)}F_{\tilde{\delta }}$ of feasible sets with error at most $\delta /m_{Y}\left(V_{N}\right)$.

**Theorem 8.**  *The (N,δ)-capacity in (117) can also be written as*

(118) $$C_{N}^{\delta }=\underset{\begin{array}{c} X:〚X〛\subseteq X,\\ \tilde{\delta }\leq \delta /m_{Y}\left(〚Y〛\right) \end{array}}{\sup}I_{\tilde{\delta }/\left|〚X〛\right|}\left(Y;X\right)bits.$$

The proof is along the same lines as the one of Theorem 5 and is omitted.

## 6. Capacity of Stationary Memoryless Uncertain Channels

In this section, we consider the special case of stationary, memoryless, uncertain channels.

Let $X^{\infty }$ be the space of X-valued discrete-time functions $x:Z_{>0}\to X$, where $Z_{>0}$ is the set of positive integers denoting the time step. Let x(a:b) denote the function $x\in X^{\infty }$ restricted over the time interval [a,b]. Let $𝒳\subseteq X^{\infty }$ be a discrete set which represents a codebook. Also, let $𝒳\left(1:n\right)=\cup_{x\in 𝒳}x\left(1:n\right)$ denote the set of all codewords up to time *n* and $𝒳\left(n\right)=\cup_{x\in 𝒳}x\left(n\right)$ denote the set of all codeword symbols in the codebook at time *n*. The codeword symbols can be viewed as the coefficients representing a continuous signal in an infinite-dimensional space. For example, transmitting one symbol per time step can be viewed as transmitting a signal of unit spectral support over time. Any discrete-time function x∈𝒳 can be selected at the transmitter, sent over a channel, received with noise perturbation, and introduced by the channel. The perturbation of the signal at the receiver due to the noise can be described as a displacement experienced by the corresponding codeword symbols x(1),x(2),…. To describe this perturbation, we consider the set-valued map $N^{\infty }:X^{\infty }\to 2^{Y^{\infty }}$, associating any point in $X^{\infty }$ to a set in $Y^{\infty }$, where $Y^{\infty }$ is the space of Y-values discrete-time functions. For any transmitted codeword $x\in 𝒳\subseteq X^{\infty }$, the corresponding received codeword lies in the set

(119) $$S_{N^{\infty }}\left(x\right)=\left\{y\in Y^{\infty }:y\in N^{\infty }\left(x\right)\right\}.$$

Also, the noise set associated with x(1:n)∈𝒳(1:n) is

(120) $$S_{N^{\infty }}\left(x\left(1:n\right)\right)=\left\{y\left(1:n\right)\in Y^{n}:y\in N^{\infty }\left(x\right)\right\},$$

where $Y^{n}={\underset{︸}{Y\times Y\times \cdots \times Y}}_{n}.$ We are now ready to define stationary, memoryless, uncertain channels.

**Definition 14.**  *A stationary, memoryless, uncertain channel is a transition mapping $N^{\infty }:X^{\infty }\to 2^{Y^{\infty }}$ that can be factorized into identical terms describing the noise experienced by the codeword symbols. Namely, there exists a set-valued map N:X→Y such that for all $n\in Z_{>0}$ and $x\left(1:n\right)\in X^{\infty }$, we have*

(121) $$S_{N^{\infty }}\left(x\left(1:n\right)\right)=N\left(x\left(1\right)\right)\times \ldots \times N\left(x\left(n\right)\right).$$

According to the definition, a stationary, memoryless, uncertain channel maps the *n*th input symbol into the *n*th output symbol in a way that does not depend on the symbols at other time steps, and the mapping is the same at all time steps. Since the channel can be characterized by the mapping *N*, to simplify the notation, we will use $S_{N}\left(.\right)$ instead of $S_{N^{\infty }}\left(.\right)$.

Another important observation is that the ϵ-perturbation channel in Definition 9 may not admit a factorization like the one in (121). For example, consider the space to be equipped with the $L^{2}$ norm, the codeword symbols to represent the coefficients of an orthogonal representation of a transmitted signal, and the noise experienced by any codeword to be within a ball of radius ϵ. In this case, if a codeword symbol is perturbed by a value close to ϵ, the perturbation of all other symbols must be close to zero.

For stationary, memoryless, uncertain channels, all received codewords lie in the set $𝒴=\cup_{x\in 𝒳}S_{N}\left(x\right)$, and the received codewords up to time *n* lie in the set $𝒴\left(1:n\right)=\cup_{x\in 𝒳}S_{N}\left(x\left(1:n\right)\right)$. Then, for any $x_{1}\left(1:n\right),x_{2}\left(1:n\right)\in 𝒳\left(1:n\right)$, we let

(122) $$\begin{array}{cc} \; & e_{N}\left(x_{1}\left(1:n\right),x_{2}\left(1:n\right)\right)\\ \; & =\frac{m_{Y}\left(S_{N}\left(x_{1}\left(1:n\right)\right)\cap S_{N}\left(x_{2}\left(1:n\right)\right)\right)}{m_{Y}\left(Y^{n}\right)}, \end{array}$$

where $m_{Y}\left(.\right)$ is an uncertainty function defined over the space of the received codewords. We also assume without loss of generality that at any time step *n*, the uncertainty associated with the space $Y^{n}$ of received codewords is $m_{Y}\left(Y^{n}\right)=1$. We also let $V_{N}=N\left(x^{*}\right)$, where $x^{*}={\mathrm{argmin}}_{x\in X}m_{Y}\left(N\left(x\right)\right)$. Thus, $V_{N}$ is the set corresponding to the minimum uncertainty introduced by the noise mapping at a single time step. Finally, we let $V_{N}^{n}=\underset{\underset{n}{︸}}{V_{N}\times V_{N}\times \cdots \times V_{N}}.$ The quantity $1-e_{\epsilon }\left(x_{1}\left(1:n\right),x_{2}\left(1:n\right)\right)$ can be viewed as the confidence we have of not confusing $x_{1}\left(1:n\right)$ and $x_{2}\left(1:n\right)$ in any transmission or, equivalently, as the amount of adversarial effort required to induce a confusion between the two codewords. For example, if the uncertainty function is constructed using a measure, then all the erroneous codewords generated by an adversary to decode $x_{2}\left(1:n\right)$ instead of $x_{1}\left(1:n\right)$ must lie inside the equivocation set $S_{N}\left(x_{1}\left(1:n\right)\right)\cap S_{N}\left(x_{2}\left(1:n\right)\right)$ whose relative size is given by (122). The smaller the equivocation set is, the larger the effort required by the adversary to induce an error must be. If the uncertainty function represents the diameter of the set, then all the erroneous codewords generated by an adversary to decode $x_{2}\left(1:n\right)$ instead of $x_{1}\left(1:n\right)$ will be close to each other, in the sense of (122).

We now introduce the notion of a *distinguishable codebook*, ensuring that every codeword cannot be confused with any other codeword, rather than with a specific one, at a given level of confidence.

**Definition 15.**  *$\left(N,\delta_{n}\right)$-distinguishable codebook.*

*For all $n\in Z_{>0}$ and $0\leq \delta_{n}<m_{Y}\left(V_{N}^{n}\right)$, a codebook ${𝒳}_{n}=𝒳\left(1:n\right)$ is $\left(N,\delta_{n}\right)$-distinguishable if for all $x_{1}\left(1:n\right),x_{2}\left(1:n\right)\in {𝒳}_{n}$, we have*

(123) $$e_{N}\left(x_{1}\left(1:n\right),x_{2}\left(1:n\right)\right)\leq \delta_{n}/\left|{𝒳}_{n}\right|.$$

It immediately follows that for any $\left(N,\delta_{n}\right)$-distinguishable codebook ${𝒳}_{n}$, we have

(124) $$e_{N}\left(x\left(1:n\right)\right)=\sum_{\begin{array}{c} x^{\prime }\left(1:n\right)\in {𝒳}_{n}:\\ x^{\prime }\left(1:n\right)\neq x\left(1:n\right) \end{array}}e_{N}\left(x\left(1:n\right),x^{\prime }\left(1:n\right)\right)\leq \delta_{n},$$

so that each codeword in ${𝒳}_{n}$ can be decoded correctly with confidence at least $1-\delta_{n}$. Definion 15 guarantees even more, namely that the confidence of not confusing any pair of codewords is at least $1-\delta_{n}/\left|{𝒳}_{n}\right|$.

We now associate with any sequence $\left\{\delta_{n}\right\}$ the largest distinguishable rate sequence $\left\{R_{\delta_{n}}\right\}$, whose elements represent the largest rates that satisfy that confidence sequence.

**Definition 16.**  *Largest $\left\{\delta_{n}\right\}$-distinguishable rate sequence.*

*For any sequence $\left\{\delta_{n}\right\}$, the largest $\left\{\delta_{n}\right\}$-distinguishable rate sequence $\left\{R_{\delta_{n}}\right\}$ is such that for all $n\in Z_{>0}$, we have*

(125) $$R_{\delta_{n}}=\underset{{𝒳}_{n}\in X_{N}^{\delta_{n}}\left(n\right)}{\sup}\frac{\log_{2}\left|{𝒳}_{n}\right|}{n}bits\;per\;symbol,$$

*where $X_{N}^{\delta_{n}}\left(n\right)=\left\{{𝒳}_{n}:{𝒳}_{n}\;is\left(N,\delta_{n}\right)-distinguishable\right\}.$*

We say that any constant rate *R* that lays below the largest $\left\{\delta_{n}\right\}$-distinguishable rate sequence is $\left\{\delta_{n}\right\}$-distinguishable. Such a $\left\{\delta_{n}\right\}$-distinguishable rate ensures the existence of a sequence of distinguishable codes that, for all $n\in Z_{>0}$, have a rate of at least *R* and confidence of at least $1-\delta_{n}$.

**Definition 17.**  *$\left\{\delta_{n}\right\}$-distinguishable rate.*

*For any sequence $\left\{\delta_{n}\right\}$, a constant rate R is said to be $\left\{\delta_{n}\right\}$-distinguishable if for all $n\in Z_{>0}$, we have*

(126) $$R\leq R_{\delta_{n}}.$$

We now give our first definition of capacity for stationary, memoryless, uncertain channels as the supremum of the $\left\{\delta_{n}\right\}$-distinguishable rates. Using this definition, transmitting at a constant rate below capacity ensures the existence of a sequence of codes that, for all $n\in Z_{>0}$, have confidence of at least $1-\delta_{n}$.

**Definition 18.**  *${\left(N,\left\{\delta_{n}\right\}\right)}_{*}$ capacity.*

*For any stationary, memoryless, uncertain channel with transition mapping N, and any given sequence $\left\{\delta_{n}\right\}$, we let*

(127) $$\begin{array}{cc} C_{N}{\left(\left\{\delta_{n}\right\}\right)}_{*} & =\sup\{R:Ris\left\{\delta_{n}\right\}-distinguishable\} \end{array}$$

(128) $$\begin{array}{cc} \; & =\underset{n\in Z_{>0}}{\inf}R_{\delta_{n}}\;\;bits\;per\;symbol. \end{array}$$

Another definition of capacity arises if, rather than the largest lower bound to the sequence of rates, one considers the least upper bound for which we can transmit, satisfying a given confidence sequence. Using this definition, transmitting at a constant rate below capacity ensures the existence of a finite-length code (rather than a sequence of codes) that satisfies at least one confidence value along the sequence $\left\{\delta_{n}\right\}$.

**Definition 19.**  *${\left(N,\left\{\delta_{n}\right\}\right)}^{*}$ capacity.*

*For any stationary, memoryless, uncertain channel with transition mapping N, and any given sequence $\left\{\delta_{n}\right\}$, we define*

(129) $$\begin{array}{cc} C_{N}{\left(\left\{\delta_{n}\right\}\right)}^{*} & =\underset{n\in Z_{>0}}{\sup}R_{\delta_{n}}\;\;bits\;per\;symbol. \end{array}$$

Next, consider Definition 19 in the case of $\left\{\delta_{n}\right\}$ as a constant sequence; namely, for all $n\in Z_{>0}$, we have $\delta_{n}=\delta \geq 0$. In this case, transmitting below capacity ensures the existence of a finite-length code that has confidence of at least 1−δ. This is a generalization of the zero-error capacity,

**Definition 20.**  *${\left(N,\delta \right)}^{*}$ capacity.*

*For any stationary, memoryless, uncertain channel with transition mapping N and any sequence $\left\{\delta_{n}\right\}$, where for all $n\in Z_{>0}$ we have $\delta_{n}=\delta \geq 0$, we define*

(130) $$\begin{array}{cc} C_{N}^{\delta *} & =\underset{n\in Z_{>0}}{\sup}R_{\delta_{n}}\;\;bits\;per\;symbol. \end{array}$$

Letting δ=0, we obtain the zero-error capacity. In this case, below capacity, there exists a code with which we can transmit with full confidence.

Finally, to give a definition of a non-stochastic analog of Shannon’s probabilistic capacity, we first say that any constant rate *R* is achievable if there exists a sequence $\delta_{n}\to 0$ as n→∞ such that *R* lays below ${lim sup}_{n}R_{\delta_{n}}$. An achievable rate *R* then ensures that for all ϵ>0, there exists an infinite sequence of distinguishable codes of rate of at least R−ϵ whose confidence tends towards one as n→∞. It follows that in this case, we can achieve communication at rate *R* with arbitrarily high confidence by choosing a sufficiently large codebook.

**Definition 21.**  *Achievable rate.*

*A constant rate R is achievable if there exists a sequence $\left\{\delta_{n}\right\}$ such that $\delta_{n}\to 0$ as n→∞ and*

(131) $$R\leq \underset{n\to \infty }{lim sup}R_{\delta_{n}}.$$

We now introduce the non-stochastic analog of Shannon’s probabilistic capacity as the supremum of the achievable rates. This means that we can pick any confidence sequence such that $\delta_{n}$ tends towards zero as n→∞. In this way, $\delta_{n}$ plays the role of the probability of error and the capacity is the largest rate that can be achieved by a sequence of codebooks with an arbitrarily high confidence level. Using this definition, transmitting at a rate below capacity ensures the existence of a sequence of codes achieving arbitrarily high confidence by increasing the codeword size.

**Definition 22.**  *${\left(N,\left\{↓0\right\}\right)}^{*}$ capacity.*

*For any stationary, memoryless, uncertain channel with transition mapping N, we define the ${\left(N,\left\{↓0\right\}\right)}^{*}$ capacity as*

(132) $$\begin{array}{cc} C_{N}{\left(\left\{↓0\right\}\right)}^{*} & =\sup\{R:R\;is\;achievable\} \end{array}$$

(133) $$\begin{array}{cc} \; & =\underset{\left\{\delta_{n}\right\}:\delta_{n}=o\left(1\right)}{\sup}\underset{n\to \infty }{lim sup}R_{\delta_{n}}. \end{array}$$

We point out the key difference between Definitions 20 and 22. Transmitting below the ${\left(N,\delta \right)}^{*}$ capacity ensures the existence of a fixed codebook that has confidence of at least 1−δ. In contrast, transmitting below the ${\left(N,\left\{↓0\right\}\right)}^{*}$ capacity allows us to achieve arbitrarily high confidence by increasing the codeword size.

**Figure 8:**
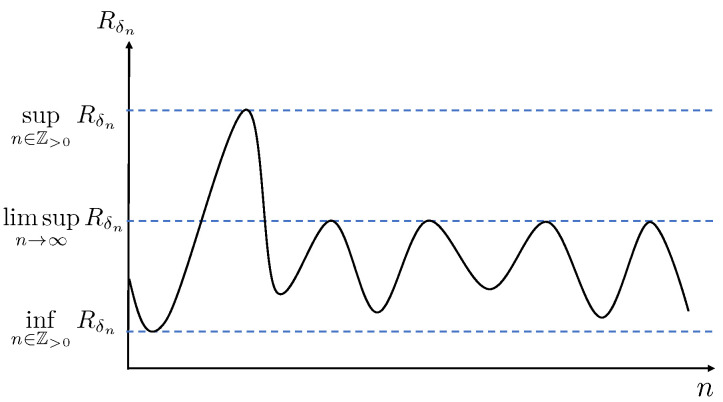
Illustration of capacities: This figure plots the sequence $R_{\delta_{n}}$ for a given sequence of $\delta_{n}$ with respect to n>0.

To give a visual illustration of the different definitions of capacity, we refer to Figure 8.

For a given sequence $\left\{\delta_{n}\right\}$, the figure sketches the largest $\left\{\delta_{n}\right\}$-distinguishable rate sequence $R_{\delta_{n}}$. According to definitions 18 and 19, the capacities $C_{N}{\left(\left\{\delta_{n}\right\}\right)}^{*}$ and $C_{N}{\left(\left\{\delta_{n}\right\}\right)}_{*}$ are given by the supremum and infimum of this sequence, respectively. On the other hand, according to Definition 22, the capacity $C_{N}{\left(\left\{↓0\right\}\right)}^{*}$ is the largest limsup over all vanishing sequences $\left\{\delta_{n}\right\}$. Assuming the figure refers to a vanishing sequence $\left\{\delta_{n}\right\}$ that achieves the supremum in (133), we have

(134) $$C_{N}{\left(\left\{\delta_{n}\right\}\right)}_{*}\geq C_{N}{\left(\left\{↓0\right\}\right)}^{*}\geq C_{N}{\left(\left\{\delta_{n}\right\}\right)}^{*}.$$

We now relate our notions of capacity to the *mutual information rate* between transmitted and received codewords. Let *X* be the UV corresponding to the transmitted codeword. This is a map of $X:X^{\infty }\to 𝒳$ and $〚X〛=𝒳\subseteq X^{\infty }$. Restricting this map to a finite time $n\in Z_{>0}$ yields another UV X(n) and 〚X(n)〛=𝒳(n)⊆X. Likewise, a codebook segment is a UV $X\left(a:b\right)={\left\{X\left(n\right)\right\}}_{a\leq n\leq b}$ of marginal range $〚X\left(a:b\right)〛=𝒳\left(a:b\right)\subseteq X^{b-a+1}.$ Likewise, let *Y* be the UV corresponding to the received codeword. It is a map of $Y:Y^{\infty }\to 𝒴$ and $〚Y〛=𝒴\subseteq Y^{\infty }$. Y(n) and Y(a:b) are UVs, and $〚Y\left(n\right)〛=𝒴\subseteq Y^{\infty }$ and $〚Y\left(a:b\right)〛=𝒴\left(a:b\right)\subseteq Y^{b-a+1}$. For a stationary, memoryless, uncertain channel with transition mapping *N*, these UVs are such that for all $n\in Z_{>0}$, y(1:n)∈〚Y(1:n)〛 and x(1:n)∈〚X(1:n)〛, and we have

(135) $$\begin{array}{cc} 〚Y(1:n)|x(1:n)〛= & \{y(1:n)\in 〚Y(1:n)〛:\\ \; & y\left(1:n\right)\in S_{N}\left(x\left(1:n\right)\right)\}, \end{array}$$

(136) $$\begin{array}{cc} 〚X(1:n)|y(1:n)〛= & \{x(1:n)\in 〚X(1:n)〛:\\ \; & y\left(1:n\right)\in S_{N}\left(x\left(1:n\right)\right)\}. \end{array}$$

Now, we define the largest $\delta_{n}$-*mutual information rate* as the supremum mutual information per unit-symbol transmission that a codeword X(1:n) can provide about Y(1:n) with confidence of at least $1-\delta_{n}/\left|〚X\left(1:n\right)〛\right|$.

**Definition 23.**  *Largest $\delta_{n}$-information rate.*

*For all $n\in Z_{>0}$, the largest $\delta_{n}$-information rate from X(1:n) to Y(1:n) is*

(137) $$R_{\delta_{n}}^{I}=\underset{\begin{array}{c} X\left(1:n\right):〚X\left(1:n\right)〛\subseteq X^{n},\\ \tilde{\delta }\leq \delta_{n}/m_{Y}\left(〚Y\left(1:n\right)〛\right) \end{array}}{\sup}\frac{I_{\tilde{\delta }/\left|〚X\left(1:n\right)〛\right|}\left(Y\left(1:n\right);X\left(1:n\right)\right)}{n}.$$

In the following theorem. we establish the relationship between $R_{\delta_{n}}$ and $R_{\delta_{n}}^{I}$.

**Theorem 9.**  *For any totally bounded, normed metric space X, disrete-time space $X^{\infty }$, stationary, memoryless, uncertain channel with transition mapping N satisfying (135) and (136), and sequence $\left\{\delta_{n}\right\}$ such that, for all $n\in Z_{>0}$, we have $0\leq \delta_{n}<m_{Y}\left(V_{N}^{n}\right)$, we have*

(138) $$R_{\delta_{n}}=\underset{\begin{array}{c} X\left(1:n\right)\in F_{\tilde{\delta }}\left(n\right),\\ \tilde{\delta }\leq \delta_{n}/m_{Y}\left(〚Y\left(1:n\right)〛\right) \end{array}}{\sup}\frac{I_{\tilde{\delta }/\left|〚X\left(1:n\right)〛\right|}\left(Y\left(1:n\right);X\left(1:n\right)\right)}{n}.$$

*We also have*

(139) $$R_{\delta_{n}}=R_{\delta_{n}}^{I}.$$

**Proof.**  The proof of the theorem is similar to the one of Theorem 4 and is given in Appendix B. □

The following coding theorem is now an immediate consequence of Theorem 9 and of our capacity definitions.

**Theorem 10.**  *For any totally bounded, normed metric space X, disrete-time space $X^{\infty }$, stationary, memoryless, uncertain channel with transition mapping N satisfying (135) and (136), and sequence $\left\{\delta_{n}\right\}$ such that for all $n\in Z_{>0}$, $0\leq \delta_{n}<m_{Y}\left(V_{N}^{n}\right)$ and $0\leq \delta <m_{Y}\left(V_{N}^{n}\right)$, we have*

(140) $$\begin{array}{cc} (1)\;\;\;\; & C_{N}{\left(\left\{\delta_{n}\right\}\right)}_{*}=\underset{n\in Z_{>0}}{\inf}R_{\delta_{n}}^{I}, \end{array}$$

(141) $$\begin{array}{cc} (2)\;\;\;\; & C_{N}{\left(\left\{\delta_{n}\right\}\right)}^{*}=\underset{n\in Z_{>0}}{\sup}R_{\delta_{n}}^{I}, \end{array}$$

(142) $$\begin{array}{cc} (3)\;\;\;\; & C_{N}{\left(\left\{↓0\right\}\right)}^{*}=\underset{\left\{\delta_{n}\right\}:\delta_{n}=o\left(1\right)}{\sup}\underset{n\to \infty }{lim sup}R_{\delta_{n}}^{I}, \end{array}$$

(143) $$\begin{array}{cc} (4)\;\;\;\; & C_{N}^{\delta *}=\underset{n\in Z_{>0}}{\sup}R_{\delta_{n}}^{I}:\forall n\in Z_{>0},\delta_{n}=\delta . \end{array}$$

Theorem 10 provides multi-letter expressions of capacity, since $R_{\delta_{n}}^{I}$ depends on $I_{\tilde{\delta }/\left|〚X\left(1:n\right)〛\right|}\left(Y\left(1:n\right);X\left(1:n\right)\right)$ according to (137). In the Supplementary Materials, we establish some special cases of uncertainty functions, confidence sequences, and classes of stationary, memoryless, uncertain channels, leading to the factorization of the mutual information and to single-letter expressions.

## 7. Conclusions and Future Directions

We presented a non-stochastic notion of information with worst-case confidence and related it to the capacity of a communication channel subject to unknown noise. Using the non-stochastic variables framework of Nair [5] and a generalization of the Kolmogorov capacity allowing some amount of overlap in the packing sets [7], we showed that the capacity equals the largest amount of information conveyed by the transmitter to the receiver, with a given level of confidence. These results are the natural generalization of Nair’s ones, obtained in a zero-error framework, and provide an information-theoretic interpretation of the geometric problem of sphere packing with overlap, as studied in [7].

Non-stochastic approaches to information and their use to quantify the performance of various engineering systems have recently received attention in the context of estimation, control, security, communication over non-linear optical channels, and learning systems [8,9,10,11,12,13,14]. We hope that the theory developed here can be useful in the future in some of these contexts. While refinements and extensions of the theory are certainly of interest, explorations of application domains are of paramount importance. There is evidence in the literature regarding the need for a non-stochastic approach to study the flow of information in complex systems, and there is a certain tradition in computer science, especially in the field of online learning, to study various problems in both a stochastic and a non-stochastic setting [15,16,17]. Nevertheless, it seems that only a few isolated efforts have been made towards the formal development of a non-stochastic information theory. Wider involvement of the community in developing alternative, even competing, theories is certainly advisable to eventually fulfill the need of these application areas.

## Data Availability

Data is contained within the article.

## Supplementary Materials

The following supporting information can be downloaded at https://www.mdpi.com/article/10.3390/e27050472/s1, Kolmogorov capacity with overlap.

## Appendix A. Proof of Lemma 1

**Proof.** Let $\left(X,Y\right)\overset{a}{↔}\left(\delta_{1},\delta_{2}\right)$. Then,

(A1) $$A\left(X;Y\right)⪯\delta_{1},$$

(A2) $$A\left(Y;X\right)⪯\delta_{2}.$$

Let

(A3) $$S_{1}=\left\{\left(y_{1},y_{2}\right):\frac{m_{X}\left(〚X\right|y_{1}〛\cap 〚X|y_{2}〛\left|\right)}{m_{X}\left(〚X〛\right)}=0\right\}.$$

Then, for all $\left(y_{1},y_{2}\right)\in S_{1}$, we have

(A4) $$\frac{m_{X}\left(〚X\right|y_{1}〛\cap 〚X|y_{2}〛\left|\right)}{m_{X}\left(〚X〛\right)}=0\leq \delta_{1}.$$

Also, if $\left(y_{1},y_{2}\right)\in S_{1}$, then

(A5) $$\frac{m_{X}\left(〚X\right|y_{1}〛\cap 〚X|y_{2}〛\left|\right)}{m_{X}\left(〚X〛\right)}\notin A\left(X;Y\right),$$

and if $\left(y_{1},y_{2}\right)\notin S_{1}$, then using (9), we have

(A6) $$\frac{m_{X}\left(〚X\right|y_{1}〛\cap 〚X|y_{2}〛\left|\right)}{m_{X}\left(〚X〛\right)}\in A\left(X;Y\right).$$

This along with (A1) and (A4) implies that (17) follows. Likewise, let

(A7) $$S_{2}=\left\{\left(x_{1},x_{2}\right):\frac{m_{Y}\left(〚Y\right|x_{1}〛\cap 〚Y|x_{2}〛\left|\right)}{m_{Y}\left(〚Y〛\right)}=0\right\}.$$

Then, for all $\left(x_{1},x_{2}\right)\in S_{2}$,

(A8) $$\frac{m_{Y}\left(〚Y\right|x_{1}〛\cap 〚Y|x_{2}〛\left|\right)}{m_{Y}\left(〚Y〛\right)}=0\leq \delta_{2}.$$

Also, if $\left(x_{1},x_{2}\right)\in S_{2}$, then

(A9) $$\frac{m_{Y}\left(〚Y\right|x_{1}〛\cap 〚Y|x_{2}〛\left|\right)}{m_{Y}\left(〚Y〛\right)}\notin A\left(Y;X\right),$$

and if $\left(y_{1},y_{2}\right)\notin S_{2}$, then using (9), we have

(A10) $$\frac{m_{Y}\left(〚Y\right|x_{1}〛\cap 〚Y|x_{2}〛\left|\right)}{m_{Y}\left(〚Y〛\right)}\in A\left(Y;X\right).$$

This along with (A2) and (A8) implies that (18) follows. Now, we prove the opposite direction of the statement. Given that for all $y_{1},y_{2}\in 〚Y〛$, we have

(A11) $$\frac{m_{X}\left(〚X\right|y_{1}〛\cap 〚X|y_{2}〛\left|\right)}{m_{X}\left(〚X〛\right)}\leq \delta_{1},$$

and for all $x_{1},x_{2}\in 〚X〛$, we have

(A12) $$\frac{m_{Y}\left(〚Y\right|x_{1}〛\cap 〚Y|x_{2}〛\left|\right)}{m_{Y}\left(〚Y〛\right)}\leq \delta_{2}.$$

Then, using the definition of A(X;Y) and A(Y;X), we have

(A13) $$A\left(X;Y\right)⪯\delta_{1},$$

(A14) $$A\left(Y;X\right)⪯\delta_{2}.$$

The statement of the lemma follows. □

## Appendix B. Proof of Theorem 9

**Proof.** We will show (138). Then, using Lemma A4 in Appendix C, (139) follows using the same argument as in the proof of Theorem 8. We proceed in three steps. First, we show that for all n>0, there exists a UV X(1:n) and $\tilde{\delta }\leq \delta_{n}/m_{Y}\left(〚Y\left(1:n\right)〛\right)$ such that $X\left(1:n\right)\in F_{\tilde{\delta }}\left(n\right)$, which implies that $F_{\tilde{\delta }}\left(n\right)$ is not empty, and so the supremum is well defined. Second, for all n>0, X(1:n), and $\tilde{\delta }$ such that

(A15) $$X\left(1:n\right)\in F_{\tilde{\delta }}\left(n\right),$$

and

(A16) $$\tilde{\delta }\leq \delta_{n}/m_{Y}\left(〚Y\left(1:n\right)〛\right),$$

we show that

$$\frac{I_{\tilde{\delta }/\left|〚X\left(1:n\right)〛\right|}\left(Y\left(1:n\right);X\left(1:n\right)\right)}{n}\leq R_{\delta_{n}}.$$

Finally, for all n>0, we show the existence of $X\left(1:n\right)\in F_{\tilde{\delta }}\left(n\right)$ and $\tilde{\delta }\leq \delta_{n}/m_{Y}\left(〚Y\left(1:n\right)〛\right)$ such that

(A17) $$\frac{I_{\tilde{\delta }/\left|〚X\left(1:n\right)〛\right|}\left(Y\left(1:n\right);X\left(1:n\right)\right)}{n}=R_{\delta_{n}}.$$

Let us begin with the first step. Consider a point $x\left(1:n\right)\in X^{n}$. Let X(1:n) be a UV such that

(A18) 〚X(1:n)〛={x(1:n)}.

Then, we hold that the marginal range of the UV Y(1:n) corresponding to the received variable is

(A19) 〚Y(1:n)〛=〚Y(1:n)|x(1:n)〛,

and therefore, for all y(1:n)∈〚Y(1:n)〛, we have

(A20) 〚X(1:n)|y(1:n)〛={x(1:n)}.

Using Definition 2 and (A18), we have

(A21) A(Y(1:n);X(1:n))=∅,

because 〚X(1:n)〛 consists of a single point, and therefore, the set in (12) is empty. On the other hand, using Definition 2 and (A20), we have

(A22) $$A\left(X\left(1:n\right);Y\left(1:n\right)\right)=\left\{\begin{array}{c} \left\{1\right\}\;\mathrm{if}\exists y_{1}\left(1:n\right),y_{2}\left(1:n\right)\\ \;\;\in 〚Y(1:n)〛,\\ \emptyset \;\mathrm{otherwise}. \end{array}\right.$$

Using (A21), and since A⪯δ holds for A=∅, we have

(A23) $$A\left(Y\left(1:n\right);X\left(1:n\right)\right)⪯\delta_{n}/\left(|〚X\left(1:n\right)〛\right|m_{Y}\left(〚Y\left(1:n\right)〛\right)).$$

Similarly, using (A22), we have

(A24) A(X(1:n);Y(1:n))⪯1.

Now, combining (A23) and (A24), we have

(A25) $$\left(X\left(1:n\right),Y\left(1:n\right)\right)\overset{a}{↔}(1,\delta_{n}/\left(|〚X\left(1:n\right)〛\right|m_{Y}\left(〚Y\left(1:n\right)〛\right))).$$

Letting $\tilde{\delta }=\delta_{n}/m_{Y}\left(〚Y\left(1:n\right)〛\right)$, this implies that $X\left(1:n\right)\in F_{\tilde{\delta }}\left(n\right)$ and that the first step of the proof is complete. To prove the second step, we define

(A26) $$\begin{array}{cc} G(n)= & \{X\left(1:n\right):〚X\left(1:n\right)〛\subseteq X^{n},\\ \; & \exists \tilde{\delta }\leq \delta_{n}/m_{Y}\left(〚Y\left(1:n\right)〛\right)\mathrm{such}\;\mathrm{that}\\ \; & \forall S_{1},S_{2}\in 〚Y\left(1:n\right)|X\left(1:n\right)〛,\\ \; & \frac{m_{Y}\left(S_{1}\cap S_{2}\right)}{m_{Y}\left(〚Y\left(1:n\right)〛\right)}\leq \frac{\tilde{\delta }}{\left|〚X(1:n)〛\right|}\}, \end{array}$$

which is a larger set than the one containing all UVs X(1:n) that are $\left(1,\tilde{\delta }/|〚X\left(1:n\right)〛|\right)$ associated with Y(1:n). Similarly to (79), it can be shown that

(A27) $$X\left(1:n\right)\in G\left(n\right)\Rightarrow 𝒳\left(1:n\right)\in X_{N}^{\delta_{n}}\left(n\right)$$

Consider now a pair, X(1:n) and $\tilde{\delta }$, such that $\tilde{\delta }\leq \delta_{n}/m_{Y}\left(〚Y\left(1:n\right)〛\right)$ and

(A28) $$X\left(1:n\right)\in F_{\tilde{\delta }}\left(n\right).$$

If $\left(X\left(1:n\right),Y\left(1:n\right)\right)\overset{d}{↔}(0,\tilde{\delta }/\left|〚X\left(1:n\right)〛|\right)$, then, using Lemma A1 in Appendix C, there exist UVs $\bar{X}\left(1:n\right)$ and $\bar{Y}\left(1:n\right)$ and $\bar{\delta }\leq \delta_{n}/m_{Y}\left(〚\bar{Y}\left(1:n\right)〛\right)$ such that

(A29) $$\left(\bar{X}\left(1:n\right),\bar{Y}\left(1:n\right)\right)\overset{a}{↔}(1,\bar{\delta }/|〚\bar{X}\left(1:n\right)〛\left|\right),$$

and

(A30) $$\begin{array}{cc} \; & |〚Y\left(1:n\right)|X\left(1:n\right){〛}_{\tilde{\delta }/\left|〚X\left(1:n\right)〛\right|}^{*}|\\ \; & =|〚\bar{Y}\left(1:n\right)\left|\bar{X}\left(1:n\right){〛}_{\bar{\delta }/\left|〚\bar{X}\left(1:n\right)〛\right|}^{*}\right|. \end{array}$$

On the other hand, if $\left(X\left(1:n\right),Y\left(1:n\right)\right)\overset{a}{↔}(1,\tilde{\delta }/\left|〚X\left(1:n\right)〛|\right)$, then (A29) and (A30) also trivially hold. It then follows that (A29) and (A30) hold for all $X\left(1:n\right)\in F_{\tilde{\delta }}\left(n\right)$. We now have

(A31) $$\begin{array}{cc} \; & I_{\tilde{\delta }/\left|〚X\left(1:n\right)〛\right|}\left(Y\left(1:n\right);X\left(1:n\right)\right)\\ \; & =\log(|〚Y\left(1:n\right)|X\left(1:n\right){〛}_{\tilde{\delta }/\left|〚X\left(1:n\right)〛\right|}^{*}|)\\ \; & \overset{\left(a\right)}{=}\log(|〚\bar{Y}\left(1:n\right)|\bar{X}\left(1:n\right){〛}_{\bar{\delta }/\left|〚\bar{X}\left(1:n\right)〛\right|}^{*}\left|\right)\\ \; & \overset{\left(b\right)}{\leq }\log(|〚\bar{X}\left(1:n\right)〛\left|\right),\\ \; & \overset{\left(c\right)}{=}\log(|\bar{𝒳}\left(1:n\right)\left|\right),\\ \; & \overset{\left(d\right)}{\leq }nR_{\delta_{n}}, \end{array}$$

where (a) follows from (A29) and (A30), (b) follows from Lemma A3 in Appendix C since $\bar{\delta }\leq \delta_{n}/m_{Y}\left(〚\bar{Y}\left(1:n\right)〛\right)<m_{Y}\left(V_{N}^{n}\right)/m_{Y}\left(〚\bar{Y}\left(1:n\right)〛\right)$, (c) follows by defining the codebook $\bar{𝒳}\left(1:n\right)$ corresponding to the UV $〚\bar{X}\left(1:n\right)〛$ , and (d) follows from the fact that using (A29) and Lemma 1, we have $\bar{X}\left(1:n\right)\in G\left(n\right)$, which implies by (A27) that $\bar{𝒳}\left(1:n\right)\in X_{N}^{\delta_{n}}\left(n\right)$. For any $n\in Z_{>0}$, let

(A32) $${𝒳}_{n}^{*}={\mathrm{argsup}}_{{𝒳}_{n}\in X_{N}^{\delta_{n}}\left(n\right)}\frac{\log(|{𝒳}_{n}|)}{n},$$

which achieves the rate $R_{\delta_{n}}$. Let $X^{*}$ be the UV whose marginal range corresponds to the codebook $X_{n}^{*}$. It follows that for all $S_{1},S_{1}\in 〚Y^{*}|X^{*}〛$, we have

(A33) $$\frac{m_{Y}\left(S_{1}\cap S_{1}\right)}{m_{Y}\left(Y^{n}\right)}\leq \frac{\delta_{n}}{|〚X^{*}〛|},$$

which implies that $m_{Y}\left(Y^{n}\right)=1$,

(A34) $$\frac{m_{Y}\left(S_{1}\cap S_{1}\right)}{m_{Y}\left(〚Y^{*}〛\right)}\leq \frac{\delta_{n}}{\left(|〚X^{*}〛|m_{Y}\left(〚Y^{*}〛\right)\right)}.$$

Letting $\delta^{*}=\delta_{n}/m_{Y}\left(〚Y^{*}〛\right)$, and using Lemma 1, we hold that $\left(X^{*},Y^{*}\right)\overset{a}{↔}(1,\delta^{*}/|〚X^{*}〛\left|\right)$, which implies

(A35) $$X^{*}\in \cup_{\tilde{\delta }\leq \delta_{n}/m_{Y}\left(〚Y^{*}〛\right)}F_{\tilde{\delta }}\left(n\right),$$

and (138) follows. □

## Appendix C. Auxiliary Results

**Lemma A1.** 
*Given a $\delta <m_{Y}\left(V_{N}\right)$, two UVs X and Y satisfying (60) and (61), and a $\tilde{\delta }\leq \delta /m_{Y}\left(〚Y〛\right)$ such that*

(A36)
$$\left(X,Y\right)\overset{d}{↔}(0,\tilde{\delta }/|〚X〛|).$$

*Then, there exist two UVs $\bar{X}$ and $\bar{Y}$ satisfying (60) and (61), and there exists a $\bar{\delta }\leq \delta /m_{Y}\left(〚\bar{Y}〛\right)$ such that*

(A37)
$$\left(\bar{X},\bar{Y}\right)\overset{a}{↔}(1,\bar{\delta }/|〚\bar{X}〛\left|\right),$$

*and*

(A38)
$$|〚Y|X{〛}_{\tilde{\delta }/\left|〚X〛\right|}^{*}|=|〚\bar{Y}\left|\bar{X}{〛}_{\bar{\delta }/\left|〚\bar{X}〛\right|}^{*}\right|.$$

**Proof.** Let the cardinality

(A39) $$|〚Y|X{〛}_{\tilde{\delta }/\left|〚X〛\right|}^{*}|=K.$$

By Property 1 of Definition 6, we hold that for all $S_{i}\in 〚Y|X{〛}_{\tilde{\delta }/\left|〚X〛\right|}^{*}$, there exists a $x_{i}\in 〚X〛$ such that $〚Y|x_{i}〛\subseteq S_{i}$. Now, consider a new UV $\bar{X}$ whose marginal range is composed of *K* elements of 〚X〛, namely

(A40) $$〚\bar{X}〛=\left\{x_{1},x_{2},\ldots ,x_{K}\right\}.$$

Let $\bar{Y}$ be the UV corresponding to the received variable. Using the fact that for all x∈X, we have $〚\bar{Y}\left|x〛=〚Y\right|x〛$ since (60) holds, and using Property 2 of Definition 6, for all $x,x^{\prime }\in 〚\bar{X}〛$, we have

(A41) $$\begin{array}{cc} \frac{m_{Y}(〚\bar{Y}|x〛\cap 〚\bar{Y}\left|x^{\prime }〛\right)}{m_{Y}\left(〚Y〛\right)} & \leq \frac{\tilde{\delta }}{\left|〚X〛\right|},\\ \; & \overset{\left(a\right)}{\leq }\frac{\tilde{\delta }}{|〚\bar{X}〛|}, \end{array}$$

where (a) follows from the fact that $〚\bar{X}〛\subseteq 〚X〛$ using (A40). Then, for all $x,x^{\prime }\in 〚\bar{X}〛$, we hold that

(A42) $$\begin{array}{cc} \frac{m_{Y}(〚\bar{Y}|x〛\cap 〚\bar{Y}\left|x^{\prime }〛\right)}{m_{Y}\left(〚\bar{Y}〛\right)} & \leq \frac{\tilde{\delta }m_{Y}\left(〚Y〛\right)}{\left|〚X〛\right|m_{Y}\left(〚\bar{Y}〛\right)},\\ \; & \overset{\left(a\right)}{\leq }\frac{\bar{\delta }}{|〚\bar{X}〛|}, \end{array}$$

where $\bar{\delta }=\tilde{\delta }m_{Y}\left(〚Y〛\right)/m_{Y}\left(〚\bar{Y}〛\right)$. Then, by Lemma 1, it follows that

(A43) $$\left(\bar{X},\bar{Y}\right)\overset{a}{↔}(1,\bar{\delta }/|〚\bar{X}〛\left|\right).$$

Since $\tilde{\delta }\leq \delta /m_{Y}\left(〚Y〛\right)$, we have

(A44) $$\bar{\delta }\leq \delta /m_{Y}\left(〚\bar{Y}〛\right)<m_{Y}\left(V_{\epsilon }\right)/m_{Y}\left(〚\bar{Y}〛\right).$$

Using (A43) and (A44), we now hold that

(A45) $$\begin{array}{cc} |〚\bar{Y}\left|\bar{X}{〛}_{\bar{\delta }/\left|〚\bar{X}〛\right|}^{*}\right| & \overset{\left(a\right)}{=}\left|〚\bar{X}〛\right|\\ \; & \overset{\left(b\right)}{=}|〚Y|X{〛}_{\tilde{\delta }/\left|〚X〛\right|}^{*}|, \end{array}$$

where (a) follows from Lemma A4 in Appendix C and (b) follows from (A39) and (A40). Hence, the statement of the lemma follows. □

**Lemma A2.** 
*Let*

(A46)
$$\left(X,Y\right)\overset{d}{↔}\left(\delta ,\delta_{2}\right).$$

*If $x\overset{\delta }{↭}x_{1}$ and $x\overset{\delta }{↭}x_{2}$, then we hold that $x_{1}\overset{\delta }{↭}x_{2}$.*

**Proof.** Let ${\left\{〚X|y_{i}〛\right\}}_{i=1}^{N}$ be the sequence of conditional range connecting *x* and $x_{1}$. Likewise, let ${\left\{〚X|{\tilde{y}}_{i}〛\right\}}_{i=1}^{\tilde{N}}$ be the sequence of conditional range connecting *x* and $x_{2}$. Now, by Definition 5, we have

(A47) $$x_{1}\in 〚X|y_{N}〛,$$

(A48) $$x_{2}\in 〚X|{\tilde{y}}_{\tilde{N}}〛,$$

(A49) $$x\in 〚X|y_{1}〛,$$

and

(A50) $$x\in 〚X|{\tilde{y}}_{1}〛$$

Then, using (9), we hold that

(A51) $$\frac{m_{X}\left(〚X\right|y_{1}〛\cap 〚X\left|{\tilde{y}}_{1}〛\right|}{m_{X}\left(〚X〛\right)}>0,$$

which implies that

(A52) $$\frac{m_{X}\left(〚X\right|y_{1}〛\cap 〚X\left|{\tilde{y}}_{1}〛\right|}{m_{X}\left(〚X〛\right)}\in A\left(X;Y\right).$$

Using the fact that

(A53) $$\left(X,Y\right)\overset{d}{↔}\left(\delta ,\delta_{2}\right),$$

we will now show that

(A54) $$\{〚X|y_{N}〛,〚X|y_{N-1}〛,\ldots 〚X|y_{1}〛,〚X|{\tilde{y}}_{1}〛,\ldots 〚X|{\tilde{y}}_{\tilde{N}}〛\},$$

is a sequence of conditional ranges connecting $x_{1}$ and $x_{2}$. Using (A52) and (A53), we hold that

(A55) $$\frac{m_{X}\left(〚X\right|y_{1}〛\cap 〚X\left|{\tilde{y}}_{1}〛\right|}{m_{X}\left(〚X〛\right)}>\delta .$$

Also, for all 1<i≤N and $1<j\leq \tilde{N}$, we have

(A56) $$\frac{m_{X}\left(〚X\right|y_{i}〛\cap 〚X\left|y_{i-1}〛\right|}{m_{X}\left(〚X〛\right)}>\delta ,$$

and

(A57) $$\frac{m_{X}\left(〚X\right|{\tilde{y}}_{j}〛\cap 〚X\left|{\tilde{y}}_{j-1}〛\right|}{m_{X}\left(〚X〛\right)}>\delta .$$

Also, we have

(A58) $$x_{1}\in 〚X\left|y_{N}〛,\;\mathrm{and}\;x_{2}\in 〚X\right|{\tilde{y}}_{\tilde{N}}〛.$$

Hence, combining (A55), (A56), (A57) and (A58), we hold that $x_{1}\overset{\delta }{↭}x_{2}$ via (A54). □

**Lemma A3.** 
*Consider two UVs X and Y. Let*

(A59)
$$\delta^{*}=\frac{\min_{y\in 〚Y〛}m_{X}\left(〚X|y〛\right)}{m_{X}\left(〚X〛\right)}.$$

*If $\delta_{1}<\delta^{*}$, then we have*

(A60)
$$|〚X|Y{〛}_{\delta_{1}}^{*}|\leq |〚Y〛|.$$

**Proof.** We will prove this by contradiction. Let

(A61) $$|〚X|Y{〛}_{\delta_{1}}^{*}|>|〚Y〛|.$$

Then, by Property 1 of Definition 6, there exists two sets $S_{1},S_{2}\in 〚X|Y{〛}_{\delta_{1}}^{*}$ and one singly $\delta_{1}$-connected set 〚X|y〛 such that

(A62) $$〚X|y〛\subseteq S_{1},\;\mathrm{and}\;〚X|y〛\subseteq S_{2}.$$

Then, we have

(A63) $$\begin{array}{cc} \frac{m_{X}\left(S_{1}\cap S_{2}\right)}{m_{X}\left(〚X〛\right)}\; & \overset{\left(a\right)}{\geq }\frac{m_{X}\left(〚X|y〛\right)}{m_{X}\left(〚X〛\right)},\\ \; & \overset{\left(b\right)}{\geq }\delta^{*},\\ \; & \overset{\left(c\right)}{>}\delta_{1}, \end{array}$$

where (a) follows from (A62) and (10), (b) follows from (A59), and (c) follows from the fact that $\delta_{1}<\delta^{*}$. However, by Property 2 of Definition 6, we have

(A64) $$\frac{m_{X}\left(S_{1}\cap S_{2}\right)}{m_{X}\left(〚X〛\right)}\leq \delta_{1}.$$

Hence, we hold that (A63) and (A64) contradict each other, which implies that (A61) does not hold. Hence, the statement of the theorem follows. □

**Lemma A4.** 
*Consider two UVs X and Y. Let*

(A65)
$$\delta^{*}=\frac{\min_{y\in 〚Y〛}m_{X}\left(〚X|y〛\right)}{m_{X}\left(〚X〛\right)}.$$

*For all $\delta_{1}<\delta^{*}$ and $\delta_{2}\leq 1$, if $\left(X,Y\right)\overset{a}{↔}\left(\delta_{1},\delta_{2}\right)$, then we have*

(A66)
$$|〚X|Y{〛}_{\delta_{1}}^{*}|=|〚Y〛|.$$

*Additionally, 〚X|Y〛 is a $\delta_{1}$-overlap family.*

**Proof.** We show that

(A67) 〚X|Y〛={〚X|y〛:y∈〚Y〛}

is a $\delta_{1}$-overlap family. First, note that 〚X|Y〛 is a cover of 〚X〛, since $〚X〛=\cup_{y\in 〚Y〛}〚X|y〛$. Second, each set in the family 〚X|Y〛 is singly $\delta_{1}$-connected via 〚X|Y〛, since trivially any two points $x_{1},x_{2}\in 〚X|y〛$ are singly $\delta_{1}$-connected via the same set. It follows that Property 1 of Definition 6 holds. Now, since $\left(X,Y\right)\overset{a}{↔}\left(\delta_{1},\delta_{2}\right)$, then by Lemma 1 for all $y_{1},y_{2}\in 〚Y〛$ we have

(A68) $$\frac{m_{X}\left(〚X\right|y_{1}〛\cap 〚X\left|y_{2}〛\right)}{m_{X}\left(〚X〛\right)}\leq \delta_{1},$$

which shows that Property 2 of Definition 6 holds. Finally, it is also easy to see that Property 3 of Definition 6 holds, since 〚X|Y〛 contains all sets 〚X|y〛. Hence, 〚X|Y〛 satisfies all the properties of $\delta_{1}$-overlap family, which implies that

(A69) $$\left|〚X|Y〛\right|\leq |〚X|Y{〛}_{\delta_{1}}^{*}|.$$

Since |〚X|Y〛|=|〚Y〛|, using Lemma A3, we also have

(A70) $$\left|〚X|Y〛\right|\geq |〚X|Y{〛}_{\delta_{1}}^{*}|.$$

Combining (A69), (A70) and the fact that 〚X|Y〛 satisfies all the properties of $\delta_{1}$-overlap family, the statement of the lemma follows. □

### Appendix C.1. Taxicab Symmetry of the Mutual Information

**Definition A1.** 
*$\left(\delta_{1},\delta_{2}\right)$-taxicab connectedness and $\left(\delta_{1},\delta_{2}\right)$-taxicab isolation.*

- *Points $\left(x,y\right),\left(x^{\prime },y^{\prime }\right)\in 〚X,Y〛$ are $\left(\delta_{1},\delta_{2}\right)$-taxicab connected via 〚X,Y〛 and are denoted by $\left(x,y\right)\overset{\delta_{1},\delta_{2}}{↭}\left(x^{\prime },y^{\prime }\right)$, if there exists a finite sequence ${\left\{\left(x_{i},y_{i}\right)\right\}}_{i=1}^{N}$ of points in 〚X,Y〛 such that $\left(x,y\right)=\left(x_{1},y_{1}\right)$, $\left(x^{\prime },y^{\prime }\right)=\left(x_{N},y_{N}\right)$, and for all 2<i≤N, we have either*
  $$\begin{array}{cc} A_{1} & =\{x_{i}=x_{i-1}\;\mathrm{and}\;\frac{m_{X}\left(〚X\right|y_{i}〛\cap 〚X\left|y_{i-1}〛\right)}{m_{X}\left(〚X〛\right)}>\delta_{1}\}, \end{array}$$
  *or*
  $$\begin{array}{cc} A_{2} & =\{y_{i}=y_{i-1}\;\mathrm{and}\;\frac{m_{Y}\left(〚Y\right|x_{i}〛\cap 〚Y\left|x_{i-1}〛\right)}{m_{Y}\left(〚Y〛\right)}>\delta_{2}\}. \end{array}$$
  *If $\left(x,y\right)\overset{\delta_{1},\delta_{2}}{↭}\left(x^{\prime },y^{\prime }\right)$ and N=2, then we say that (x,y) and $\left(x^{\prime },y^{\prime }\right)$ are singly $\left(\delta_{1},\delta_{2}\right)$-taxicab connected, i.e., either $y=y^{\prime }$ and $x,x^{\prime }\in 〚X|y〛$ or $x=x^{\prime }$ and $y,y^{\prime }\in 〚Y|x〛$.*
- *A set S⊆〚X,Y〛 is (singly) $\left(\delta_{1},\delta_{2}\right)$-taxicab connected via 〚X,Y〛 if every pair of points in the set is (singly) $\left(\delta_{1},\delta_{2}\right)$-taxicab connected in 〚X,Y〛.*
- *Two sets $S_{1},S_{2}\subseteq 〚X,Y〛$ are $\left(\delta_{1},\delta_{2}\right)$-taxicab isolated via 〚X,Y〛 if no point in $S_{1}$ is $\left(\delta_{1},\delta_{2}\right)$-taxicab connected to any point in $S_{2}$.*

**Definition A2.** 
*Projection of a set*

- *The projection $S_{x}^{+}$ of a set S⊆〚X,Y〛 on the x-axis is defined as*
  (A71)
  $$S_{x}^{+}=\left\{x:\left(x,y\right)\in S\right\}.$$
- *The projection $S_{y}^{+}$ of a set S⊆〚X,Y〛 on the y-axis is defined as*
  (A72)
  $$S_{y}^{+}=\left\{y:\left(x,y\right)\in S\right\}.$$

**Definition A3.** 
*$\left(\delta_{1},\delta_{2}\right)$-taxicab family*

*A $\left(\delta_{1},\delta_{2}\right)$-taxicab family of 〚X,Y〛, denoted by $〚X,Y{〛}_{\left(\delta_{1},\delta_{2}\right)}^{*}$, is a largest family of distinct sets covering 〚X,Y〛 such that*

1. *Each set in the family is $\left(\delta_{1},\delta_{2}\right)$-taxicab connected and contains at least one singly $\delta_{1}$-connected set of form 〚X|y〛×{y} and at least one singly $\delta_{2}$-connected set of the form 〚Y|x〛×{x}.*
2. *The measure of overlap between the projections on the x-axis and y-axis of any two distinct sets in the family are at most $\delta_{1}m_{X}\left(〚X〛\right)$ and $\delta_{2}m_{Y}\left(〚Y〛\right)$, respectively.*
3. *For every singly $\left(\delta_{1},\delta_{2}\right)$-connected set, there exists a set in the family containing it.*

We now show that when $\left(X,Y\right)\overset{d}{↔}\left(\delta_{1},\delta_{2}\right)$ hold, the cardinality of the $\left(\delta_{1},\delta_{2}\right)$-taxicab family is same as the cardinality of the 〚X|Y〛$\delta_{1}$-overlap family and 〚Y|X〛$\delta_{2}$-overlap family.

**Proof of Theorem 3.** We will show that $|〚X,Y{〛}_{\left(\delta_{1},\delta_{2}\right)}^{*}|=|〚X|Y{〛}_{\delta_{1}}^{*}|$. Then, $|〚X,Y{〛}_{\left(\delta_{1},\delta_{2}\right)}^{*}|=|〚Y|X{〛}_{\delta_{2}}^{*}|$ can be derived along the same lines. Hence, the statement of the theorem follows. First, we will show that

(A73) $$D=\{S_{x}^{+}:S\in 〚X,Y{〛}_{\left(\delta_{1},\delta_{2}\right)}^{*}\},$$

satisfies all the properties of $〚X|Y{〛}_{\delta_{1}}^{*}$. Since $〚X,Y{〛}_{\left(\delta_{1},\delta_{2}\right)}^{*}$ is a covering of 〚X,Y〛, we have

(A74) $$\cup_{S_{x}^{+}\in D}S_{x}^{+}=〚X〛,$$

which implies that D is a covering of 〚X〛. Consider a set $S\in 〚X,Y{〛}_{\left(\delta_{1},\delta_{2}\right)}^{*}$. For all $\left(x,y\right),\left(x^{\prime },y^{\prime }\right)\in S$, (x,y) and $\left(x^{\prime },y^{\prime }\right)$ are $\left(\delta_{1},\delta_{2}\right)$-taxicab connected. Then, there exists a taxicab sequence of the form

$$\left(x,y\right),\left(x_{1},y\right),\left(x_{1},y_{1}\right),\ldots \left(x_{n-1},y^{\prime }\right),\left(x^{\prime },y^{\prime }\right).$$

such that either $A_{1}$ or $A_{2}$ in Definition A1 is true. Then, the sequence $\left\{y,y_{1},\ldots ,y_{n-1},y^{\prime }\right\}$ yields a sequence of conditional range ${\left\{〚X|{\tilde{y}}_{j}〛\right\}}_{j=1}^{n+1}$ such that for all 1<j≤n+1,

(A75) $$\frac{m_{X}\left(〚X\right|{\tilde{y}}_{j}〛\cap 〚X\left|{\tilde{y}}_{j-1}〛\right)}{m_{X}\left(〚X〛\right)}>\delta_{1},$$

(A76) $$x\in 〚X|{\tilde{y}}_{1}〛,\;\mathrm{and}\;x^{\prime }\in 〚X|{\tilde{y}}_{n+1}〛.$$

Hence, $x\overset{\delta_{1}}{↭}x^{\prime }$ via 〚X|Y〛. Hence, $S_{x}^{+}$ is $\delta_{1}$-connected via 〚X|Y〛. Also, S contains at least one singly $\delta_{1}$-connected set of the form 〚X|y〛×{y}, which implies $〚X|y〛\subseteq S_{x}^{+}$. Hence, $S_{x}^{+}$ contains at least one singly $\delta_{1}$-connected set of the form 〚X|y〛. Hence, D satisfies Property 1 in Definition 6. For all $S_{1},S_{2}\in 〚X,Y{〛}_{\left(\delta_{1},\delta_{2}\right)}^{*}$, we have

(A77) $$m_{X}\left(S_{1,x}^{+}\cap S_{2,x}^{+}\right)\leq \delta_{1}m_{X}\left(〚X〛\right),$$

using Property 2 in Definition A3. Hence, D satisfies Property 2 in Definition 6. Using Property 3 in Definition A3, we hold that for all 〚X|y〛×{y}, there exists a set $S\left(y\right)\in 〚X,Y{〛}_{\left(\delta_{1},\delta_{2}\right)}^{*}$ containing it. This implies that for all 〚X|y〛∈〚X|Y〛, we have

(A78) $${〚X|y〛\subseteq S\left(y\right)}_{x}^{+}.$$

Hence, D satisfies Property 3 in Definition 6. Thus, D satisfies all the three properties of $〚X|Y{〛}_{\delta_{1}}^{*}$. This implies, along with Theorem 2, that

(A79) $$\left|D\right|=|〚X|Y{〛}_{\delta_{1}}^{*}|,$$

which implies that

(A80) $$|〚X,Y{〛}_{\left(\delta_{1},\delta_{2}\right)}^{*}|=|〚X|Y{〛}_{\delta_{1}}^{*}|.$$

Hence, the statement of the theorem follows. □
