# Supplementary material for: Kolmogorov Capacity with Overlap
Source: Entropy (Basel). 2025 Apr 27;27(5):472. doi: 10.3390/e27050472 (PMC12109944; doi:10.3390/e27050472)
Supplement: Supplementary file 1 [file entropy-27-00472-s001.zip › entropy-3527220-supplementary.pdf]

Supplementary Materials: Kolmogorov Capacity with Overlap

Anshuka Rangi , Massimo Franceschetti

S1. Factorization of the Mutual Information

To obtain sufficient conditions for the factorization of the mutual information, we choose to work with a *product uncertainty function*.

Assumption S1. *(Product uncertainty function). The uncertainty function of a Cartesian product of n sets can be factorized in the product of its terms, namely for any $n \in \mathbb{Z}_{>0}$ and $\mathscr{S} \subseteq [\![Y]\!]$, such that for*

$$\mathscr{S} = \mathscr{S}_1 \times \mathscr{S}_2 \times \ldots \times \mathscr{S}_n, \tag{S1}$$

we have

$$m_{\mathscr{Y}}(\mathscr{S}) = m_{\mathscr{Y}}(\mathscr{S}_1) \times m_{\mathscr{Y}}(\mathscr{S}_2) \times \ldots \times m_{\mathscr{Y}}(\mathscr{S}_n). \tag{S2}$$

We also assume that the product uncertainty function satisfies a union bound.

Assumption S2. *(Union bound). For all $\mathscr{S}_1, \mathscr{S}_2 \subseteq [\![Y]\!]$, we have*

$$m_{\mathscr{Y}}(\mathscr{S}_1 \cup \mathscr{S}_2) \leq m_{\mathscr{Y}}(\mathscr{S}_1) + m_{\mathscr{Y}}(\mathscr{S}_2). \tag{S3}$$

Before stating the main result of this section, we state the following useful lemma.

Lemma S1. *Let $X(1:n)$ and $Y(1:n)$ be two UVs such that*

$$[\![X(1:n)]\!] = [\![X(1)]\!] \times [\![X(2)]\!] \ldots \times [\![X(n)]\!], \tag{S4}$$

and for all $x(1:n) \in [\![X(1:n)]\!]$, we have

$$[\![Y(1:n)|x(1:n)]\!] = [\![Y(1)|x(1)]\!] \times \ldots [\![Y(n)|x(n)]\!]. \tag{S5}$$

Let

$$0 \leq \delta < \min_{\substack{1 \leq i \leq n, \\ x(i) \in [\![X(i)]\!]}} m_{\mathscr{Y}}([\![Y(i)|x(i)]\!]). \tag{S6}$$

Finally, let either

$$(X(1:n), Y(1;n)) \overset{d}{\leftrightarrow} (0, \delta^n), \text{ or} \tag{S7}$$

$$(X(1:n), Y(1;n)) \overset{a}{\leftrightarrow} (1, \delta^n). \tag{S8}$$

Under Assumption S1, we hold the following:

1. *The cartesian product $\prod_{i=1}^n [\![Y(i)|X(i)]\!]^*_\delta$ is a covering of $[\![Y(1:n)]\!]$.*
2. *Every $\mathscr{S} \in \prod_{i=1}^n [\![Y(i)|X(i)]\!]^*_\delta$ is δ^n-connected and contains at least one singly δ^n-connected set of the form $[\![Y(1:n)|x(1:n)]\!]$.*
3. *For every singly δ^n-connected set of the form $[\![Y(1:n)|x(1:n)]\!]$, there exists a set in $\prod_{i=1}^n [\![Y(i)|X(i)]\!]^*_\delta$ containing it; namely, for all $x(1:n) \in [\![X(1:n)]\!]$, there exists a set $\mathscr{S} \in \prod_{i=1}^n [\![Y(i)|X(i)]\!]^*_\delta$ such that $[\![Y(1:n)|x(1:n)]\!] \subseteq \mathscr{S}$.*

4. *For all* $\mathscr{S}_1, \mathscr{S}_2 \in \prod_{i=1}^{n} [\![Y(i)|X(i)]\!]_\delta^*$, *we have*

$$\frac{m_{\mathscr{Y}}(\mathscr{S}_1 \cap \mathscr{S}_2)}{m_{\mathscr{Y}}([\![Y(1:n)]\!])} \le \delta(\hat{\delta}(n))^{n-1}, \tag{S9}$$

where

$$\hat{\delta}(n) = \max_{\substack{1 \le i \le n, \\ \mathscr{S} \in [\![Y(i)|X(i)]\!]_\delta^*}} \frac{m_{\mathscr{Y}}(\mathscr{S})}{m_{\mathscr{Y}}([\![Y(i)]\!])}. \tag{S10}$$

Proof. The proof is given in Section S4. □

Under Assumption S1 and Assumption S2, given two UVs that can be written in Cartesian product form and that are either associated at level $(0, \delta^n)$ or disassociated at level $(1, \delta^n)$, we now obtain an upper bound on the mutual information at level δ^n in terms of the sum of the mutual information at level δ of their components. An analogous result in the stochastic setting states that the mutual information between two n-dimensional random variables $X^n = \{X_1, \ldots, X_n\}$ and $Y^n = \{Y_1, \ldots, Y_n\}$ is at most the sum of the component-wise mutual information, namely

$$I(X^n; Y^n) \le \sum_{i=1}^{n} I(X_i; Y_i), \tag{S11}$$

where $I(X;Y)$ represents the Shannon mutual information between two random variables X and Y. In contrast to the stochastic setting, here, the mutual information is associated with a confidence parameter δ^n that is re-scaled to δ when this is decomposed into the sum of n terms.

Theorem S1. *Let* $X(1:n)$ *and* $Y(1:n)$ *be two UVs such that*

$$[\![X(1:n)]\!] = [\![X(1)]\!] \times [\![X(2)]\!] \ldots [\![X(n)]\!], \tag{S12}$$

and for all $x(1:n) \in [\![X(1:n)]\!]$, *we have*

$$[\![Y(1:n)|x(1:n)]\!] = [\![Y(1)|x(1)]\!] \times \ldots [\![Y(n)|x(n)]\!]. \tag{S13}$$

Also, let

$$0 \le \delta < \frac{\min_{1 \le i \le n, x(i) \in [\![X(i)]\!]} m_{\mathscr{Y}}([\![Y(i)|x(i)]\!])}{\max_{1 \le i \le n} |[\![X(i)]\!]|}. \tag{S14}$$

Finally, let either

$$(X(1:n), Y(1:n)) \overset{d}{\leftrightarrow} (0, \delta^n), \text{ or} \tag{S15}$$

$$(X(1:n), Y(1:n)) \overset{a}{\leftrightarrow} (1, \delta^n). \tag{S16}$$

Under Assumptions S1 and 2, we have

$$I_{\delta^n}(Y(1:n); X(1:n)) \le \sum_{i=1}^{n} I_\delta(Y(i); X(i)). \tag{S17}$$

Proof. First, we will show that for all $\mathscr{S} \in \prod_{i=1}^{n} [\![Y(i)|X(i)]\!]_\delta^*$, there exist a point $x_{\mathscr{S}}(1:n) \in [\![X(1:n)]\!]$ and a set $\mathscr{D}(\mathscr{S}) \in [\![Y(1:n)|X(1:n)]\!]_{\delta^n}^*$ such that

$$[\![Y(1:n)|x_{\mathscr{S}}(1:n)]\!] \subseteq \mathscr{S}, \tag{S18}$$

$$[\![Y(1:n)|x_{\mathscr{S}}(1:n)]\!] \subseteq \mathscr{D}(\mathscr{S}). \tag{S19}$$

Using this result, we will then show that

$$|\prod_{i=1}^{n}[\![Y(i)|X(i)]\!]^*_{\delta}| \geq |[\![Y(1:n)|X(1:n)]\!]^*_{\delta^n}|, \tag{S20}$$

which immediately implies (S17).

Let us begin with the first step. We have

$$\begin{aligned}\delta &\overset{(a)}{<} \frac{\min_{1\leq i\leq n, x(i)\in[\![X(i)]\!]} m_{\mathscr{Y}}([\![Y(i)|x(i)]\!])}{\max_{1\leq i\leq n}|[\![X(i)]\!]|},\\ &\overset{(b)}{\leq} \min_{1\leq i\leq n, x(i)\in[\![X(i)]\!]} m_{\mathscr{Y}}([\![Y(i)|x(i)]\!]),\end{aligned} \tag{S21}$$

where (a) follows from (S14), and (b) follows from the fact that for all $1 \leq i \leq n$, we have $|[\![X(i)]\!]| \geq 1$.

Now, consider a set $\mathscr{S} \in \prod_{i=1}^{n}[\![Y(i)|X(i)]\!]^*_{\delta}$. Using (S21), by Lemma S1 part 2, we hold that there exists a point $x'(1:n) \in [\![X(1:n)]\!]$ such that

$$[\![Y(1:n)|x'(1:n)]\!] \subseteq \mathscr{S}. \tag{S22}$$

Now, using (S21), part 1 in Lemma S1, and [Definition 6 Main Doc], we have

$$\begin{aligned}\cup_{\mathscr{S}\in\prod_{i=1}^{n}[\![Y(i)|X(i)]\!]^*_{\delta}}\mathscr{S} &= [\![Y(1:n)]\!],\\ &= \cup_{\mathscr{D}\in[\![Y(1:n)|X(1:n)]\!]^*_{\delta^n}}\mathscr{D}.\end{aligned} \tag{S23}$$

Using (S23) and Property 3 of [Definition 6 Main Doc], there exists a set $\mathscr{D}(x'(1:n)) \in [\![Y(1:n)|X(1:n)]\!]^*_{\delta^n}$ such that

$$[\![Y(1:n)|x'(1:n)]\!] \subseteq \mathscr{D}(x'(1:n)). \tag{S24}$$

Letting $x_{\mathscr{S}}(1:n) = x'(1:n)$ and $\mathscr{D}(\mathscr{S}) = \mathscr{D}(x'(1:n))$ in (S22) and (S24), we hold that (S18) and (S19) follow.

We now proceed with proving (S20). We distinguish two cases. In the first case, there exist two sets $\mathscr{S} \in \prod_{i=1}^{n}[\![Y(i)|X(i)]\!]^*_{\delta}$ and $\mathscr{D}_1 \in [\![Y(1:n)|X(1:n)]\!]^*_{\delta^n}$ such that

$$\mathscr{D}_1 \cap \mathscr{S} \setminus \mathscr{D}(\mathscr{S}) \neq \emptyset. \tag{S25}$$

In the second case, the sets $\mathscr{S}$ and $\mathscr{D}_1$ satisfying (S25) do not exist. We will show that the first case is not possible, and in the second case, we conclude that (S20) holds.

To rule out the first case, consider two points of

$$y_1(1:n) \in [\![Y(1:n)|x_{\mathscr{S}}(1:n)]\!] \subseteq \mathscr{D}(\mathscr{S}) \tag{S26}$$

and

$$y_2(1:n) \in \mathscr{D}_1 \cap \mathscr{S} \setminus \mathscr{D}(\mathscr{S}). \tag{S27}$$

If $(X(1:n), Y(1:n) \overset{a}{\leftrightarrow} (1,\delta^n)$, then we have

$$\begin{aligned}
&\delta^n |[\![X(1:n)]\!]| \\
&\overset{(a)}{<} \left(\frac{\min_{1\le i\le n, x(i)\in[\![X(i)]\!]} m_{\mathscr{Y}}([\![Y(i)|x(i)]\!])}{\max_{1\le i\le n} |[\![X(i)]\!]|} \right)^n |[\![X(1:n)]\!]|, \\
&\overset{(b)}{\le} \left(\min_{\substack{1\le i\le n,\\ x(i)\in[\![X(i)]\!]}} m_{\mathscr{Y}}([\![Y(i)|x(i)]\!]) \right)^n, \\
&\overset{(c)}{\le} \min_{x(1:n)\in[\![X(1:n)]\!]} m_{\mathscr{Y}}([\![Y(1:n)|x(1:n)]\!]), \\
&\overset{(d)}{\le} \frac{\min_{x(1:n)\in[\![X(1:n)]\!]} m_{\mathscr{Y}}([\![Y(1:n)|x(1:n)]\!])}{m_{\mathscr{Y}}([\![Y(1:n)]\!])},
\end{aligned} \tag{S28}$$

where (a) follows from (S14), (b) follows from (S12) and the fact that

$$|[\![X(1)]\!] \times [\![X(2)]\!] \dots [\![X(n)]\!]| \le (\max_{1\le i\le n} |[\![X(i)]\!]|)^n, \tag{S29}$$

(c) follows from (S13) and Assumption S1, and (d) follows from [(10) MainDoc] and the facts that $[\![Y(1:n)]\!] \subseteq \mathscr{Y}^n$ and $m_{\mathscr{Y}}(\mathscr{Y}^n) = 1$. Combining (S28), Assumption S2, and Lemma S2 in Section S5, we hold that there exists a point $y(1:n) \in [\![Y(1:n)|x_{\mathscr{S}}(1:n)]\!]$ such that for all $[\![Y(1:n)|x(1:n)]\!] \in [\![Y(1:n)|X(1:n)]\!] \setminus \{[\![Y(1:n)|x_{\mathscr{S}}(1:n)]\!]\}$, we have.

$$y(1:n) \notin [\![Y(1:n)|x(1:n)]\!]. \tag{S30}$$

Without loss of generality, let

$$y_1(1:n) = y(1:n). \tag{S31}$$

It now follows that $y_1(1:n)$ and $y_2(1:n)$ cannot be δ^n-connected. This follows because

$$y_1(1:n) \in [\![Y(1:n)|x_{\mathscr{S}}(1:n)]\!], \tag{S32}$$

$$y_2(1:n) \notin [\![Y(1:n)|x_{\mathscr{S}}(1:n)]\!], \tag{S33}$$

(S30) and $(X(1:n), Y(1:n) \overset{a}{\leftrightarrow} (1,\delta^n)$, so that there does not exist a sequence $\{[\![Y(1:n)|x_i(1:n)]\!]\}_{i=1}^N$ such that for all $1 < i \le N$

$$\frac{m_{\mathscr{Y}}([\![Y(1:n)|x_i(1:n)]\!] \cap [\![Y(1:n)|x_{i-1}(1:n)]\!])}{m_{\mathscr{Y}}([\![Y(1:n)]\!])} > \delta^n. \tag{S34}$$

On the other hand, if $(X(1:n), Y(1:n) \overset{d}{\leftrightarrow} (0,\delta^n)$, then using [Theorem 2 Main Doc],we hold that $[\![Y(1:n)|X(1:n)]\!]^*_{\delta^n}$ is a δ^n-isolated partition. Thus, $y_1(1:n)$ and $y_2(1:n)$ are not δ^n-connected, since $y_1(1:n) \in \mathscr{D}(\mathscr{S})$ and $y_2(1:n) \in \mathscr{D}_1 \cap \mathscr{S} \setminus \mathscr{D}(\mathscr{S})$. However, since $y_1(1:n), y_2(1:n) \in \mathscr{S}$, and $\mathscr{S}$ is δ^n-connected using (S21) and 2) in Lemma S1, we hold that $y_1(1:n) \overset{\delta^n}{\leftrightsquigarrow} y_2(1:n)$. This contradiction implies that $\mathscr{D}_1$ and $\mathscr{S}$ do not exist.

In the second case, if $\mathscr{S}$ and $\mathscr{D}_1$ do not exist, then for all $\mathscr{S}' \in \prod_{i=1}^n [\![Y(i)|X(i)]\!]^*_\delta$ and $\mathscr{D}' \in [\![Y(1:n)|X(1:n)]\!]^*_{\delta^n}$, we have

$$\mathscr{D}' \cap \mathscr{S}' \setminus \mathscr{D}(\mathscr{S}') = \varnothing, \tag{S35}$$

which implies that

$$\begin{aligned}\mathscr{S}' &\stackrel{(a)}{=} \cup_{\mathscr{D}' \in [\![Y(1:n)|X(1:n)]\!]^*_{\delta^n}} (\mathscr{S}' \cap \mathscr{D}'),\\ &\stackrel{(b)}{\subseteq} \cup_{\mathscr{D}' \in [\![Y(1:n)|X(1:n)]\!]^*_{\delta^n}} \Big(\mathscr{D}(\mathscr{S}') \cup (\mathscr{S}' \cap \mathscr{D}' \setminus \mathscr{D}(\mathscr{S}')\Big),\\ &= \mathscr{D}(\mathscr{S}') \cup_{\mathscr{D}' \in [\![Y(1:n)|X(1:n)]\!]^*_{\delta^n}} (\mathscr{S}' \cap \mathscr{D}' \setminus \mathscr{D}(\mathscr{S}')),\\ &\stackrel{(c)}{=} \mathscr{D}(\mathscr{S}'),\end{aligned} \tag{S36}$$

where (a) follows from (S23), (b) follows from the trivial fact that, for any three sets, $\mathscr{A}$, $\mathscr{B}$ and $\mathscr{C}$,

$$\mathscr{A} \cap \mathscr{B} \subseteq \mathscr{C} \cup (\mathscr{A} \cap \mathscr{B} \setminus \mathscr{C}), \tag{S37}$$

and (c) follows from (S35). Combining (S36) and (S23), we hold that

$$|\prod_{i=1}^{n} [\![Y(i)|X(i)]\!]^*_{\delta}| \geq |[\![Y(1:n)|X(1:n)]\!]^*_{\delta^n}|. \tag{S38}$$

The statement of the theorem now follows. □

The following corollary shows that the bound-in Theorem S1 is tight in the zero-error case.

Corollary S1. *Let $X(1:n)$ and $Y(1:n)$ satisfy (S12) and (S13). Under Assumptions S1 and S2, we hold that*

$$I_0(Y(1:n); X(1:n)) = \sum_{i=1}^{n} I_0(Y(i); X(i)). \tag{S39}$$

Proof. The proof is along the same lines as the one of Theorem S1. For all $X(1:n)$ and $Y(1:n)$, if $\mathcal{A}(Y;X) = \emptyset$, then

$$(X(1:n), Y(1:n)) \stackrel{a}{\leftrightarrow} (1,0); \tag{S40}$$

otherwise,

$$(X(1:n), Y(1:n)) \stackrel{d}{\leftrightarrow} (0,0). \tag{S41}$$

Hence, either (S15) or (S16) holds for $\delta = 0$. Now, by replacing $\delta = 0$ in $1) - 4)$ of Lemma S1, we hold that $\prod_{i=1}^{n} [\![Y(i)|X(i)]\!]^*_0$ satisfies all the properties of a 0-overlap family. Combining this fact and Theorem S1, the statement of the corollary follows. □

S2. Single letter expressions

We are now ready to present sufficient conditions leading to single-letter expressions for $C(\{\delta_n\})_*$, $C_N^{\delta *}$, and $C(\{\delta_n\})^*$. Under these conditions, the multi dimensional optimization problem of searching for a codebook that achieves capacity over an interval of size n can be reduced to searching for a codebook over a single step. The single letter characterization of $C_N(\{\downarrow 0\})^*$ is particularly challenging, which is also evident from the fact that the single letter characterization may not always exist, as in the case of the zero-error capacity. Nevertheless, we show that under some sufficient conditions, $C_N(\{\downarrow 0\})^*$ is lower bounded by a single-letter expression.

First, we start with the single-letter expression for $C(\{\delta_n\})^*$.

Theorem S2. *For any stationary memoryless uncertain channel N and for any $0 \leq \delta_1 < m_{\mathscr{Y}}(V_N)$, let $\bar{X} \in \mathscr{F}_{\bar{\delta}}(1)$ be a UV over one time step associated with a one-dimensional codebook that achieves*

the capacity $C_N(\{\delta_1\})^* = C_N(\{\delta_1\})_* = R_{\delta_1}$*, and let* $\bar{Y}$ *be the UV corresponding to the received codeword, namely*

$$\begin{aligned} C_N(\{\delta_1\})^* &= I_{\bar{\delta}/|[\![\bar{X}]\!]|}(\bar{Y};\bar{X}) \\ &= \sup_{\substack{X(1)\in\mathscr{F}_{\bar{\delta}}(1):\\ \bar{\delta}\le\delta_1/m_{\mathscr{Y}}([\![Y(1)]\!])}} I_{\bar{\delta}/|[\![X(1)]\!]|}(Y(1);X(1)). \end{aligned} \tag{S42}$$

If for all one-dimensional codewords $x \in \mathscr{X} \setminus [\![\bar{X}]\!]$*, there exists a set* $\mathscr{S} \in [\![\bar{Y}|\bar{X}]\!]^*_{\bar{\delta}/|[\![\bar{X}]\!]|}$ *such that the uncertainty region* $[\![Y|x]\!] \subseteq \mathscr{S}$*,* $\bar{\delta}(1 + 1/|[\![\bar{X}]\!]|) \le \delta_1/m_{\mathscr{Y}}([\![\bar{Y}]\!])$*, and for all* $n > 1$*, we have* $0 \le \delta_n \le (\bar{\delta}m_{\mathscr{Y}}(V_N)/|[\![\bar{X}]\!]|)^n$*, then, under Assumptions S1 and S2, we hold that the n-dimensional capacity*

$$C_N(\{\delta_n\})^* = I_{\bar{\delta}/|[\![\bar{X}]\!]|}(\bar{Y};\bar{X}). \tag{S43}$$

Proof. Let

$$[\![\bar{X}(1:n)]\!] = \underbrace{[\![\bar{X}]\!] \times \cdots \times [\![\bar{X}]\!]}_{n}, \tag{S44}$$

and

$$[\![\bar{Y}(1:n)]\!] = \underbrace{[\![\bar{Y}]\!] \times \cdots \times [\![\bar{Y}]\!]}_{n}. \tag{S45}$$

For all $n > 0$, we have

$$\begin{aligned} \delta_n &\le \left(\frac{\bar{\delta}m_{\mathscr{Y}}(V_N)}{|[\![\bar{X}]\!]|}\right)^n, \\ &\overset{(a)}{\le} \left(\frac{\delta_1 m_{\mathscr{Y}}(V_N)}{|[\![\bar{X}]\!]|m_{\mathscr{Y}}([\![\bar{Y}]\!])}\right)^n, \\ &\overset{(b)}{\le} \left(\frac{\delta_1}{|[\![\bar{X}]\!]|}\right)^n, \\ &\overset{(c)}{<} (m_{\mathscr{Y}}(V_N))^n, \\ &\overset{(d)}{=} m_{\mathscr{Y}}(V_N^n), \end{aligned} \tag{S46}$$

where (a) follows from the fact that $\bar{\delta} \le \delta_1/m_{\mathscr{Y}}([\![\bar{Y}]\!])$, (b) follows from $m_{\mathscr{Y}}(V_N) \le m_{\mathscr{Y}}([\![\bar{Y}]\!])$, (c) follows from $\delta_1 < m_{\mathscr{Y}}(V_N)$ and $|[\![\bar{X}]\!]| \ge 1$, and (d) follows from Assumption S1.

We now proceed in three steps. First, using (S46) and [Theorem 9 MainDoc],we have

$$\begin{aligned} C_N(\{\delta_n\})^* &= \sup_{n\in\mathbb{Z}_{>0}} R_{\delta_n} \\ &\ge R_{\delta_1} = R^I_{\delta_1} = I_{\bar{\delta}/|[\![\bar{X}]\!]|}(\bar{Y};\bar{X}). \end{aligned} \tag{S47}$$

Second, we will show that for all $n \in \mathbb{Z}_{>0}$, we have

$$\begin{aligned} &\sup_{\substack{X(1:n):[\![X(1:n)]\!]\subseteq\mathscr{X}^n,\\ \bar{\delta}\le\delta_n/m_{\mathscr{Y}}([\![Y(1:n)]\!])}} I_{\bar{\delta}/|[\![X(1:n)]\!]|}(Y(1:n);X(1:n)) \\ &\le nI_{\bar{\delta}/|[\![\bar{X}]\!]|}(\bar{Y};\bar{X}). \end{aligned} \tag{S48}$$

Finally, using (S46), [Theorem 9 MainDoc] and (S48), for all $n \in \mathbb{Z}_{>0}$, we hold that

$$R_{\delta_n} = R^I_{\delta_n} \le I_{\bar{\delta}/|[\![\bar{X}]\!]|}(\bar{Y};\bar{X}), \tag{S49}$$

which implies that
$$C_N(\{\delta_n\})^* = \sup_{n\in\mathbb{Z}_{>0}} R_{\delta_n} \leq I_{\bar{\delta}/|[\![\bar{X}]\!]|}(\bar{Y};\bar{X}). \tag{S50}$$
Using (S47) and (S50), the result (S43) follows.

Now, we only need to prove (S48). We will prove this by contradiction. Consider a UV $X(1:n)$ and
$$\delta' \leq \delta_n / m_{\mathscr{Y}}([\![Y(1:n)]\!]), \tag{S51}$$
such that
$$\begin{aligned}&|[\![Y(1:n)|X(1:n)]\!]^*_{\delta'/|[\![X(1:n)]\!]|}|\\&> |\prod_{i=1}^{n}[\![\bar{Y}|\bar{X}]\!]^*_{\bar{\delta}/|[\![\bar{X}]\!]|}|.\end{aligned} \tag{S52}$$
We will show that (S52) cannot hold using the following four claims, whose proofs appear in Section S6.

- **Claim 1:** If (S52) holds, then there exist two UVs $\tilde{X}(1:n)$ and $\tilde{Y}(1:n)$ such that letting
$$\tilde{\delta} = \frac{\delta' m_{\mathscr{Y}}([\![Y(1:n)]\!])}{m_{\mathscr{Y}}([\![\tilde{Y}(1:n)]\!])}, \tag{S53}$$
we have
$$\tilde{\delta} \leq \frac{\delta_n}{m_{\mathscr{Y}}([\![\tilde{Y}(1:n)]\!])}, \tag{S54}$$
$$(\tilde{X}(1:n),\tilde{Y}(1:n)) \overset{a}{\leftrightarrow} (1,\tilde{\delta}/|[\![\tilde{X}(1:n)]\!]|), \tag{S55}$$
and
$$|[\![\tilde{Y}(1:n)|\tilde{X}(1:n)]\!]^*_{\tilde{\delta}/|[\![\tilde{X}(1:n)]\!]|}| > |\prod_{i=1}^{n}[\![\bar{Y}|\bar{X}]\!]^*_{\bar{\delta}/|[\![\bar{X}]\!]|}|. \tag{S56}$$
- **Claim 2:** For all $\tilde{x}(1:n) \in [\![\tilde{X}(1:n)]\!]$, there exists a set $\mathscr{S} \in \prod_{i=1}^{n}[\![\bar{Y}|\bar{X}]\!]^*_{\bar{\delta}/|[\![\bar{X}]\!]|}$ such that
$$[\![\tilde{Y}(1:n)|\tilde{x}(1:n)]\!] \subseteq \mathscr{S}. \tag{S57}$$
- **Claim 3:** Using Claims 1 and 2, there exist a set $\mathscr{S} \in \prod_{i=1}^{n}[\![\bar{Y}|\bar{X}]\!]^*_{\bar{\delta}/|[\![\bar{X}]\!]|}$ and two points $\tilde{x}_1(1:n), \tilde{x}_2(1:n) \in [\![\tilde{X}(1:n)]\!]$ such that
$$[\![\tilde{Y}(1:n)|\tilde{x}_1(1:n)]\!], [\![\tilde{Y}(1:n)|\tilde{x}_2(1:n)]\!] \subset \mathscr{S}. \tag{S58}$$
Also, there exists a $1 \leq i^* \leq n$ such that
$$\begin{aligned}&\frac{m_{\mathscr{Y}}([\![\tilde{Y}(i^*)|\tilde{x}_1(i^*)]\!] \cap [\![\tilde{Y}(i^*)|\tilde{x}_2(i^*)]\!])}{(m_{\mathscr{Y}}([\![\tilde{Y}(1:n)]\!]))^{1/n}}\\&\leq \frac{\bar{\delta}}{|[\![\bar{X}]\!]|}.\end{aligned} \tag{S59}$$
- **Claim 4:** Using Claim 3, we hold that there exist two UVs X' and Y' and that $\delta^* \leq \delta_1 / m_{\mathscr{Y}}([\![Y']\!])$ such that
$$|[\![Y'|X']\!]^*_{\delta^*/|[\![X']\!]|}| > |[\![\bar{Y}|\bar{X}]\!]^*_{\bar{\delta}/|[\![\bar{X}]\!]|}|. \tag{S60}$$

The result in Claim 4 contradicts (S42). It follows that (S52) cannot hold and the proof of Theorem S2 is complete. □

Since $C_N^{\delta *}$ is a special case of $C_N(\{\delta_n\})^*$ for which the sequence δ_n is constant, it seems natural to use Theorem S2 to obtain a single-letter expression for $C_N^{\delta *}$ as well. However, the range of δ_n in Theorem S2 restricts the obtained single-letter expression for this case to the zero-error capacity C_N^{0*} only. To see this, note that δ_n in Theorem S2 is constrained to

$$\begin{aligned}\delta_n &\leq (\bar{\delta} m_{\mathscr{Y}}(V_N)/|[\![\bar{X}]\!]|)^n \\ &< (m_{\mathscr{Y}}^2(V_N)/m_{\mathscr{Y}}([\![\bar{Y}]\!]))^n.\end{aligned} \tag{S61}$$

It follows that if $m_{\mathscr{Y}}(V_N) < m_{\mathscr{Y}}(\mathscr{Y}) = 1$, then we have $\delta_n = o(1)$ as $n \to \infty$. Hence, in this case, a single letter expression for $C_N^{\delta *}$ can only be obtained for $\delta = 0$. On the other hand, if $m_{\mathscr{Y}}(V_N) = m_{\mathscr{Y}}(\mathscr{Y})$, then for any $0 \leq \delta < m_{\mathscr{Y}}(V_N)$, the codebook can only contain a single codeword, and in this case, we have $C_N^{\delta *} = 0$. We conclude that the only non-trivial single-letter expression is obtained for the zero-error capacity, as stated next.

Corollary S2. *For any stationary, memoryless, uncertain channel N, let $\bar{X} \in \mathscr{F}_0(1)$ be a UV over one time step associated with a one-dimensional codebook that achieves the capacity $C_N(\{0\})^* = C_N(\{0\})_* = R_0$, and let $\bar{Y}$ be the UV corresponding to the received codeword, namely*

$$\begin{aligned}C_N(\{0\})^* &= I_0(\bar{Y};\bar{X}) \\ &= \sup_{X(1)\in\mathscr{F}_0(1)} I_0(Y(1);X(1)).\end{aligned} \tag{S62}$$

If for all one-dimensional codewords $x \in \mathscr{X} \setminus [\![\bar{X}]\!]$, there exists a set $\mathscr{S} \in [\![\bar{Y}|\bar{X}]\!]^*_{\delta/|[\![\bar{X}]\!]|}$ such that the uncertainty region $[\![Y|x]\!] \subseteq \mathscr{S}$, then, under Assumptions S1 and S2, we hold that the n-dimensional zero-error capacity

$$C_N^{0*} = I_0(\bar{Y};\bar{X}). \tag{S63}$$

Next, we present the sufficient conditions leading to the single letter expression for $C(\{\delta_n\})_*$.

Theorem S3. *For any stationary, memoryless, uncertain channel N, and for any $0 \leq \delta_1 < m_{\mathscr{Y}}(V_N)$, let $\bar{X} \in \mathscr{F}_{\bar{\delta}}(1)$ be a UV over one time step associated with a one-dimensional codebook that achieves the capacity $C_N(\{\delta_1\})_* = C_N(\{\delta_1\})^* = R_{\delta_1}$, and let $\bar{Y}$ be the UV corresponding to the received codeword, namely*

$$\begin{aligned}C_N(\{\delta_1\})_* &= I_{\bar{\delta}/|[\![\bar{X}]\!]|}(\bar{Y};\bar{X}) \\ &= \sup_{\substack{X(1)\in\mathscr{F}_{\bar{\delta}}(1), \\ \bar{\delta}\leq\delta_1/m_{\mathscr{Y}}([\![Y(1)]\!])}} I_{\bar{\delta}/|[\![X(1)]\!]|}(Y(1);X(1)).\end{aligned} \tag{S64}$$

Let

$$\hat{\delta} = \max_{\mathscr{S}\in[\![\bar{Y}|\bar{X}]\!]^*_{\bar{\delta}/|[\![\bar{X}]\!]|}} \frac{m_{\mathscr{Y}}(\mathscr{S})}{m_{\mathscr{Y}}([\![\bar{Y}]\!])}. \tag{S65}$$

If for all $n > 1$, we have $\bar{\delta}(\hat{\delta}|[\![\bar{X}]\!]|)^{n-1} \leq \delta_n < 1$, then under Assumption S1 we hold that the n-dimensional capacity

$$C_N(\{\delta_n\})_* = I_{\bar{\delta}/|[\![\bar{X}]\!]|}(\bar{Y};\bar{X}). \tag{S66}$$

Proof. First, we show that for all $n \in \mathbb{Z}_{>0}$ and $\delta_n \geq \bar{\delta}(\hat{\delta}|[\![\bar{X}]\!]|)^{n-1}$, there exists a codebook $\tilde{\mathscr{X}}(1:n) \in \mathscr{X}_N^{\delta_n}(n)$ such that

$$|\tilde{\mathscr{X}}(1:n)| = |\prod_{i=1}^{n}[\![\bar{Y}|\bar{X}]\!]^*_{\bar{\delta}/|[\![\bar{X}]\!]|}|. \tag{S67}$$

This, along with Definition 16, implies that for all $n \in \mathbb{Z}_{>0}$ and $\delta_n \geq \bar{\delta}(\hat{\delta}|[\![\bar{X}]\!]|)^{n-1}$, we have

$$R_{\delta_n} \geq I_{\bar{\delta}/|[\![\bar{X}]\!]|}(\bar{Y};\bar{X}), \tag{S68}$$

and therefore

$$C_N(\{\delta_n\})_* = \inf_{n\in\mathbb{Z}_{>0}} R_{\delta_n} \geq I_{\bar{\delta}/|[\![\bar{X}]\!]|}(\bar{Y};\bar{X}). \tag{S69}$$

Second, we show that

$$C_N(\{\delta_n\})_* \leq I_{\bar{\delta}/|[\![\bar{X}]\!]|}(\bar{Y};\bar{X}). \tag{S70}$$

Thus, combining (S69) and (S70), we hold that (S66) follows and the proof is complete.

We now start with the first step of showing (S69). Without loss of generality, we assume that $[\![\bar{Y}|\bar{X}]\!]^*_{\bar{\delta}/|[\![\bar{X}]\!]|} > 1$; otherwise,

$$I_{\bar{\delta}/|[\![\bar{X}]\!]|}(\bar{Y};\bar{X}) = 0, \tag{S71}$$

and (S66) holds trivially by the definition of $C_N(\{\delta_n\})_*$. Let

$$[\![\bar{X}(1:n)]\!] = \underbrace{[\![\bar{X}]\!] \times \cdots \times [\![\bar{X}]\!]}_{n}, \tag{S72}$$

and

$$[\![\bar{Y}(1:n)]\!] = \underbrace{[\![\bar{Y}]\!] \times \cdots \times [\![\bar{Y}]\!]}_{n}. \tag{S73}$$

Then, using 4) in Lemma S1 and the fact that N is a stationary, memoryless, uncertain channel, for all $\mathscr{S}_1, \mathscr{S}_2 \in \prod_{i=1}^{n}[\![\bar{Y}|\bar{X}]\!]^*_{\bar{\delta}/|[\![\bar{X}]\!]|}$, we have

$$\begin{aligned} \frac{m_{\mathscr{Y}}(\mathscr{S}_1 \cap \mathscr{S}_2)}{m_{\mathscr{Y}}([\![\bar{Y}(1:n)]\!])} &\leq \frac{\bar{\delta}\hat{\delta}^{n-1}}{|[\![\bar{X}]\!]|}, \\ &\overset{(a)}{\leq} \frac{\delta_n}{(|[\![\bar{X}]\!]|)^n}, \\ &\overset{(b)}{=} \frac{\delta_n}{|[\![\bar{X}(1:n)]\!]|}, \end{aligned} \tag{S74}$$

where (a) follows from the assumption in the theorem that $\delta_n \geq \bar{\delta}(\hat{\delta}|[\![\bar{X}]\!]|)^{n-1}$, and (b) follows from (S72).

Using 2) in Lemma S1, we hold that for all $\mathscr{S}_i \in \prod_{i=1}^{n}[\![\bar{Y}|\bar{X}]\!]^*_{\bar{\delta}/|[\![\bar{X}]\!]|}$, there exists $x_i(1:n) \in [\![\bar{X}(1:n)]\!]$ such that

$$[\![\bar{Y}(1:n)|x_i(1:n)]\!] \subseteq \mathscr{S}_i. \tag{S75}$$

Now, let

$$K = |\prod_{i=1}^{n}[\![\bar{Y}|\bar{X}]\!]^*_{\bar{\delta}/|[\![\bar{X}]\!]|}|. \tag{S76}$$

Consider a new UV $\tilde{X}(1:n)$ whose marginal range is composed of K elements of $[\![\bar{X}(1:n)]\!]$, namely

$$[\![\tilde{X}(1:n)]\!] = \{x_1(1:n), \ldots x_K(1:n)\}. \tag{S77}$$

Let $\tilde{Y}(1:n)$ be the UV corresponding to the received variable. For all $x(1:n) \in [\![\tilde{X}(1:n)]\!]$, we have $[\![\tilde{Y}(1:n)|x(1:n)]\!] = [\![\bar{Y}(1:n)|x(1:n)]\!]$ since N is a stationary, memoryless, uncertain channel. Using (S74), (S75), it now follows that for all $x(1:n), x'(1:n) \in [\![\tilde{X}(1:n)]\!]$, we have

$$\begin{aligned} &\frac{m_{\mathscr{Y}}([\![\tilde{Y}(1:n)|x(1:n)]\!] \cap [\![\tilde{Y}(1:n)|x'(1:n)]\!])}{m_{\mathscr{Y}}([\![\bar{Y}(1:n)]\!])} \\ &\leq \frac{\delta_n}{|[\![\bar{X}(1:n)]\!]|}, \\ &\overset{(a)}{\leq} \frac{\delta_n}{|[\![\tilde{X}(1:n)]\!]|}, \end{aligned} \tag{S78}$$

where (a) follows from the fact that using (S77), we have $[\![\tilde{X}(1:n)]\!] \subseteq [\![\bar{X}(1:n)]\!]$. This implies that for all $x(1:n), x'(1:n) \in [\![\tilde{X}(1:n)]\!]$,

$$\begin{aligned} &e_N(x(1:n), x'(1:n)) \\ &= \frac{m_{\mathscr{Y}}(S_N(x(1:n)) \cap S_N(x'(1:n)))}{m_{\mathscr{Y}}(\mathscr{Y}^n)}, \\ &\overset{(a)}{=} \frac{m_{\mathscr{Y}}([\![\tilde{Y}(1:n)|x(1:n)]\!] \cap [\![\tilde{Y}(1:n)|x'(1:n)]\!])}{m_{\mathscr{Y}}(\mathscr{Y}^n)}, \\ &\overset{(b)}{\leq} \frac{\delta_n}{|[\![\tilde{X}(1:n)]\!]|} \frac{m_{\mathscr{Y}}([\![\bar{Y}(1:n)]\!])}{m_{\mathscr{Y}}(\mathscr{Y}^n)}, \\ &\overset{(c)}{\leq} \frac{\delta_n}{|[\![\tilde{X}(1:n)]\!]|}, \end{aligned} \tag{S79}$$

where (a) follows from the fact that N is stationary and memoryless, and for all $x(1:n) \in \mathscr{X}^n$, we have

$$[\![Y(1:n)|x(1:n)]\!] = S_N(x(1:n)), \tag{S80}$$

(b) follows from (S78), and (c) follows from [(10) MainDoc] and $[\![\bar{Y}(1:n)]\!] \subseteq \mathscr{Y}^n$. This implies that the codebook $\tilde{\mathcal{X}}(1:n)$ corresponding to the UV $\tilde{X}(1:n)$ is (N, δ_n)-distinguishable. It follows that (S68) and (S69) hold, and the the first step of the proof follows.

Now, we prove the second step. We have

$$\begin{aligned} C_N(\{\delta_n\})_* &= \inf_{n \in \mathbb{Z}_{>0}} R_{\delta_n}, \\ &\overset{(a)}{\leq} R_{\delta_1}, \\ &\overset{(b)}{=} R^I_{\delta_1}, \\ &\overset{(c)}{=} I_{\bar{\delta}/|[\![\bar{X}]\!]|}(\bar{Y}; \bar{X}), \end{aligned} \tag{S81}$$

where (a) follows from the fact that

$$\inf_{n \in \mathbb{Z}_{>0}} R_{\delta_n} \leq R_{\delta_1}, \tag{S82}$$

(b) follows from the fact that, since $\delta_1 < m_{\mathscr{Y}}(V_N)$, we hold that

$$R_{\delta_1} = R^I_{\delta_1}, \tag{S83}$$

using [Theorem 9 MainDoc]and (c) follows from the fact that

$$R^I_{\delta_1} = I_{\bar{\delta}/|[\![\bar{X}]\!]|}(\bar{Y}; \bar{X}), \tag{S84}$$

using [(133) MainDoc], [(134) MainDoc], and (S64). Hence, the second step of the proof follows. □

Finally, we present the sufficient conditions leading a lower bound on $C_N(\{\downarrow 0\})^*$ in terms of a single letter expression.

Theorem S4. *Let $0 \leq \delta_1 < m_{\mathscr{Y}}(V_N)$. For any stationary, memoryless, uncertain channel N, let $X^* \in \mathscr{F}_{\delta^*}(1)$ be a UV over one time step associated with a one-dimensional codebook that achieves the largest one-dimensional δ_1-capacity, and let Y^* be the UV corresponding to the received codeword, namely X^* achieves $\sup_{\delta_1<m_{\mathscr{Y}}(V_N)} C_N(\{\delta_1\})_* = \sup_{\delta_1<m_{\mathscr{Y}}(V_N)} C_N(\{\delta_1\})^* = \sup_{\delta_1<m_{\mathscr{Y}}(V_N)} R_{\delta_1}$, and we have*

$$\begin{aligned}
&\sup_{\delta_1<m_{\mathscr{Y}}(V_N)} C_N(\{\delta_1\})_* \\
&= I_{\delta^*/|[\![X^*]\!]|}(Y^*;X^*) \\
&= \sup_{\delta_1<m_{\mathscr{Y}}(V_N)} \sup_{\substack{X(1)\in\mathscr{F}_{\tilde{\delta}}(1),\\ \tilde{\delta}\leq\delta_1/m_{\mathscr{Y}}([\![Y(1)]\!])}} I_{\tilde{\delta}/|[\![X(1)]\!]|}(Y(1);X(1)).
\end{aligned} \tag{S85}$$

Let

$$\hat{\delta}_* = \max_{\mathscr{S}\in[\![Y^*|X^*]\!]^*_{\delta^*/|[\![X^*]\!]|}} \frac{m_{\mathscr{Y}}(\mathscr{S})}{m_{\mathscr{Y}}([\![Y^*]\!])}. \tag{S86}$$

If $\hat{\delta}_*|[\![X^*]\!]| < 1$, then under Assumption S1 we hold that the n-dimensional capacity

$$C_N(\{\downarrow 0\})^* \geq I_{\delta^*/|[\![X^*]\!]|}(Y^*;X^*). \tag{S87}$$

Proof. Consider a sequence of $\{\delta_n\}$ such that $\delta_1 = \delta^*$ and $\delta_n = \delta^*(\hat{\delta}_*|[\![X^*]\!]|)^{n-1}$ for $n > 1$. Then, using Theorem S3 for this sequence $\{\delta_n\}$, we have that

$$C_N(\{\delta_n\})_* = I_{\delta^*/|[\![X^*]\!]|}(Y^*;X^*). \tag{S88}$$

Now, since $\hat{\delta}_*|[\![X^*]\!]| < 1$ using the assumption in the theorem, we have

$$\lim_{n\to\infty} \delta_n = 0. \tag{S89}$$

Using (S88) and (S89), we hold that

$$\begin{aligned}
C_N(\{\downarrow 0\})^* &\geq \sup_{\{\delta'_n\}:\delta'_n=o(1)} C_N(\{\delta'_n\})_*, \\
&\geq C_N(\{\delta_n\})_*, \\
&= I_{\delta^*/|[\![X^*]\!]|}(Y^*;X^*).
\end{aligned} \tag{S90}$$

□

S3. Examples

To cast our sufficient conditions for the existence of single letter expressions of capacity in a concrete setting, we now provide some examples and compute the corresponding capacity.

In the following, we represent stationary, memoryless, uncertain channels in graph form. Let $\mathcal{G}(V,E)$ be a directed graph, where V is the set of vertices and E is the set of edges. The vertices represent input and output codeword symbols, namely $V = \mathscr{X} \cup \mathscr{Y}$. A directed edge from node $x \in \mathscr{X}$ to node $y \in \mathscr{Y}$, denoted by $x \to y$, shows that given

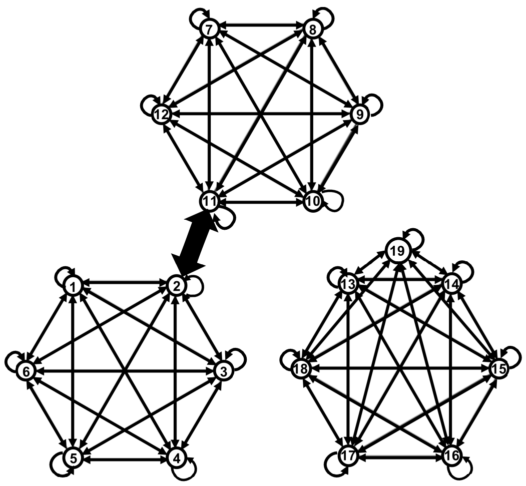

Figure S1. Channel described in Example S1. It consists of three complete graphs, and some additional edges. The thick solid arrow into node $y = 11$ represents multiple edges connecting all the nodes in the set $\{1,2,3,4,5,6\}$ to node 11. Similarly, all the nodes in the set $\{7,8,9,10,11,12\}$ are connected to node 2.

symbol x is transmitted, y may be received at the output of the channel. It follows that for all $x \in \mathscr{X}$, the channel transition map representing the noise experienced by each codeword is given by

$$N(x) = \{y : (x \to y) \in E\}. \tag{S91}$$

Example S1. *We consider a channel with*

$$\mathscr{X} = \mathscr{Y} = \{1, 2, 3, \ldots, 19\}. \tag{S92}$$

To define the channel transition map, we let for all $x \in \{1,2,3,4,5,6\}$

$$N(x) = \{1,2,3,4,5,6,11\}, \tag{S93}$$

for all $x \in \{7,8,9,10,11,12\}$

$$N(x) = \{7,8,9,10,11,12,2\}, \tag{S94}$$

and for all $x \in \{13,14,15,16,17,18,19\}$

$$N(x) = \{13,14,15,16,17,18,19\}. \tag{S95}$$

The corresponding graph is depicted in Figure S1. For any $\mathcal{Y}_n \subseteq \mathscr{Y}^n$*, we define the uncertainty function* $m_{\mathscr{Y}}(\mathcal{Y}_n)$ *in terms of cardinality*

$$m_{\mathscr{Y}}(\mathcal{Y}_n) = \frac{|\mathcal{Y}_n|}{|\mathscr{Y}^n|}. \tag{S96}$$

Note that for all $n \in \mathbb{Z}_{>0}$*, we have* $m_{\mathscr{Y}}(\mathscr{Y}^n) = 1$*.*

It is easy to show that $m_{\mathscr{Y}}(.)$ *satisfies Assumption S1. Namely, for* $n = 1$*, we hold that for all* $\mathcal{Y} \subseteq \mathscr{Y}$*,*

$$m_{\mathscr{Y}}(\mathcal{Y}) = \frac{|\mathcal{Y}|}{|\mathscr{Y}|}. \tag{S97}$$

Let $\mathcal{Y}_n = \mathcal{Y}(1) \times \mathcal{Y}(2) \ldots \times \mathcal{Y}(n)$, where $\mathcal{Y}(i) \subseteq \mathscr{Y}$. Then, we have

$$\begin{aligned} & m_{\mathscr{Y}}(\mathcal{Y}_n) \\ & = m_{\mathscr{Y}}(\mathcal{Y}(1) \times \mathcal{Y}(2) \ldots \times \mathcal{Y}(n)), \\ & = \frac{|\mathcal{Y}(1) \times \mathcal{Y}(2) \ldots \times \mathcal{Y}(n)|}{|\mathscr{Y}^n|}, \\ & \overset{(a)}{=} \frac{|\mathcal{Y}(1)|}{|\mathscr{Y}|} \frac{|\mathcal{Y}(2)|}{|\mathscr{Y}|} \cdots \frac{|\mathcal{Y}(n)|}{|\mathscr{Y}|}, \\ & \overset{(b)}{=} m_{\mathscr{Y}}(\mathcal{Y}(1)) m_{\mathscr{Y}}(\mathcal{Y}(2)) \ldots m_{\mathscr{Y}}(\mathcal{Y}(n)), \end{aligned} \tag{S98}$$

where (a) follows from the fact that for any two sets $\mathscr{S}_1$ and $\mathscr{S}_2$, $|\mathscr{S}_1 \times \mathscr{S}_2| = |\mathscr{S}_1||\mathscr{S}_2|$, and (b) follows from (S97). It follows that $m_{\mathscr{Y}}(.)$ satisfies Assumption S1.

A similar argument shows that $m_{\mathscr{Y}}(.)$ also satisfies Assumption S2. Namely, let $\mathcal{Y}_n = \mathcal{Y}(1) \cup \mathcal{Y}(2) \ldots \cup \mathcal{Y}(n)$, where $\mathcal{Y}(i) \in \mathscr{Y}$. Then, we have

$$\begin{aligned} & m_{\mathscr{Y}}(\mathcal{Y}_n) \\ & = m_{\mathscr{Y}}(\mathcal{Y}(1) \cup \mathcal{Y}(2) \ldots \cup \mathcal{Y}(n)), \\ & = \frac{|\mathcal{Y}(1) \cup \mathcal{Y}(2) \ldots \cup \mathcal{Y}(n)|}{|\mathscr{Y}|}, \\ & \overset{(a)}{\leq} \frac{|\mathcal{Y}(1)|}{|\mathscr{Y}|} + \frac{|\mathcal{Y}(2)|}{|\mathscr{Y}|} + \ldots + \frac{|\mathcal{Y}(n)|}{|\mathscr{Y}|}, \\ & \overset{(b)}{=} m_{\mathscr{Y}}(\mathcal{Y}(1)) + m_{\mathscr{Y}}(\mathcal{Y}(2)) + \ldots + m_{\mathscr{Y}}(\mathcal{Y}(n)), \end{aligned} \tag{S99}$$

where (a) follows from the fact that for any two sets $\mathscr{S}_1$ and $\mathscr{S}_2$, $|\mathscr{S}_1 \cup \mathscr{S}_2| \leq |\mathscr{S}_1| + |\mathscr{S}_2|$, and (b) follows from (S97). It follows that $m_{\mathscr{Y}}(.)$ satisfies Assumption S2.

We now compute the capacity $C_N(\{\delta_n\})^*$ for $\delta_1 = 2/9$ and for all $n > 1$ $\delta_n = (7/342)^n$.

Since V_N contains seven elements, we hold that $m_{\mathscr{Y}}(V_N) = 7/19$, and $\delta_1 < m_{\mathscr{Y}}(V_N)$. Consider a UV $\bar{X}$ representing a one-dimensional codebook such that

$$[\![\bar{X}]\!] = \{1, 7, 13\}. \tag{S100}$$

It follows that the corresponding output UV $\bar{Y}$ is such that

$$[\![\bar{Y}]\!] = \{1, 2, 3, \ldots, 18, 19\}. \tag{S101}$$

Letting $\bar{\delta} = 1/6$, we hold that $\bar{\delta}/|[\![X]\!]| = 1/18$ and the overlap family

$$[\![\bar{Y}|\bar{X}]\!]^*_{1/18} = \{\mathscr{S}_1, \mathscr{S}_2\}, \tag{S102}$$

where

$$\mathscr{S}_1 = \cup_{x \in \{1,7\}} N(x), \tag{S103}$$

$$\mathscr{S}_2 = \cup_{x \in \{13\}} N(x). \tag{S104}$$

We now show that $\bar{X}$ satisfies the sufficient conditions in Theorem S2. First, we note that for all $x \in \{2, 3, 4, 5, 6, 8, 9, 10, 11, 12\}$, we hold that $[\![\bar{Y}|x]\!] \subseteq \mathscr{S}_1$, and for all $x \in \{14, 15, 16, 17, 18, 19\}$, we have $[\![\bar{Y}|x]\!] \subseteq \mathscr{S}_2$. It follows that for all $x \in \mathscr{X} \setminus [\![\bar{X}]\!] = \{2, 3, 4, 5, 6, 8, 9, 10, 11, 12, 14, 15, 16, 17, 18, 19\}$, the uncertainity region $[\![\bar{Y}|x]\!] \subseteq \mathscr{S}$, where $\mathscr{S} \in [\![\bar{Y}|\bar{X}]\!]^*_{\bar{\delta}/|[\![\bar{X}]\!]|}$. Second, we hold that

$$\bar{\delta}(1 + 1/|[\![\bar{X}]\!]|) = 2/9 \leq \delta_1 / m_{\mathscr{Y}}([\![\bar{Y}]\!]). \tag{S105}$$

Third, we note that $\bar{\delta} = 1/6$,

$$\bar{\delta} m_{\mathscr{Y}}(V_N)/|[\![\bar{X}]\!]| = 7/342. \tag{S106}$$

It follows that for all $n > 1$, *we hold that* $\delta_n \leq (\bar{\delta} m_{\mathscr{Y}}(V_N)/|[\![\bar{X}]\!]|)^n$.

Since all the sufficient conditions in Theorem S2 are satisfied, we have

$$C_N(\{\delta_n\})^* = \log_2 |[\![\bar{Y}|\bar{X}]\!]^*_{1/18}| = 1. \tag{S107}$$

Example S2. *We now consider the same channel as in Example S1, shown in Figure S1, and we compute the capacity* C_N^{0*}. *We consider the one-dimensional codebook* $\bar{X}$ *in* (S100), *and the corresponding output UV* $\bar{Y}$ *in* (S101). *Then, we have*

$$[\![\bar{Y}|\bar{X}]\!]^*_0 = \{\mathscr{S}_1, \mathscr{S}_2\}, \tag{S108}$$

where

$$\mathscr{S}_1 = \cup_{x \in \{1,7\}} N(x), \tag{S109}$$

$$\mathscr{S}_2 = \cup_{x \in \{13\}} N(x). \tag{S110}$$

We now show that $\bar{X}$ *satisfies the sufficient conditions in Corollary S2. We note that for all* $x \in \{2,3,4,5,6,8,9,10,11,12\}$, *we hold that* $[\![\bar{Y}|x]\!] \subseteq \mathscr{S}_1$, *and for all* $x \in \{14,15,16,17,18,19\}$, *we have* $[\![\bar{Y}|x]\!] \subseteq \mathscr{S}_2$. *It follows that for all* $x \in \mathscr{X} \setminus [\![\bar{X}]\!] = \{2,3,4,5,6,8,9,10,11,12,14,15,16,17,18,19\}$, *the uncertainity region* $[\![\bar{Y}|x]\!] \subseteq \mathscr{S}$, *where* $\mathscr{S} \in [\![\bar{Y}|\bar{X}]\!]^*_0$.

Since all the sufficient conditions in Corollary S2 are satisfied, we have

$$C_N^{0*} = \log_2 |[\![\bar{Y}|\bar{X}]\!]^*_0| = 1. \tag{S111}$$

Example S3. *We now consider the same channel as in Example S1, shown in Figure S1, and we compute the capacity* $C_N(\{\delta_n\})_*$ *for* $\delta_1 = (2/6)^3$ *and for all* $n > 1$ $\delta_n = (2/6)^3((7/19)^3 3)^{n-1}$.

For any $\mathcal{Y}_n \subseteq \mathscr{Y}^n$, *we define the uncertainty function* $m_{\mathscr{Y}}(\mathcal{Y}_n)$ *in terms of cardinality*

$$m_{\mathscr{Y}}(\mathcal{Y}_n) = \left(\frac{|\mathcal{Y}_n|}{|\mathscr{Y}^n|}\right)^3. \tag{S112}$$

Note that for all $n \in \mathbb{Z}_{>0}$, *we have* $m_{\mathscr{Y}}(\mathscr{Y}^n) = 1$. *It is easy to show that* $m_{\mathscr{Y}}(.)$ *satisfies Assumption S1. For* $n = 1$, *we hold that for all* $\mathcal{Y} \subseteq \mathscr{Y}$,

$$m_{\mathscr{Y}}(\mathcal{Y}) = \left(\frac{|\mathcal{Y}|}{|\mathscr{Y}|}\right)^3. \tag{S113}$$

Let $\mathcal{Y}_n = \mathcal{Y}(1) \times \mathcal{Y}(2) \ldots \times \mathcal{Y}(n)$, *where* $\mathcal{Y}(i) \in \mathscr{Y}$. *Then, we have*

$$\begin{aligned}
&m_{\mathscr{Y}}(\mathcal{Y}_n) \\
&= m_{\mathscr{Y}}(\mathcal{Y}(1) \times \mathcal{Y}(2) \ldots \times \mathcal{Y}(n)), \\
&= \left(\frac{|\mathcal{Y}(1) \times \mathcal{Y}(2) \ldots \times \mathcal{Y}(n)|}{|\mathscr{Y}^n|}\right)^3, \\
&\overset{(a)}{=} \left(\frac{|\mathcal{Y}(1)|}{|\mathscr{Y}|}\right)^3 \left(\frac{|\mathcal{Y}(2)|}{|\mathscr{Y}|}\right)^3 \ldots \left(\frac{|\mathcal{Y}(n)|}{|\mathscr{Y}|}\right)^3, \\
&\overset{(b)}{=} m_{\mathscr{Y}}(\mathcal{Y}(1)) m_{\mathscr{Y}}(\mathcal{Y}(2)) \ldots m_{\mathscr{Y}}(\mathcal{Y}(n)),
\end{aligned} \tag{S114}$$

where (a) *follows from the fact that for any two sets* $\mathscr{S}_1$ *and* $\mathscr{S}_2$, $|\mathscr{S}_1 \times \mathscr{S}_2| = |\mathscr{S}_1||\mathscr{S}_2|$, *and* (b) *follows from* (S113). *It follows that* $m_{\mathscr{Y}}(.)$ *satisfies Assumption S1.*

Since $m_{\mathscr{Y}}(V_N) = (7/19)^3$, *we have* $\delta_1 < m_{\mathscr{Y}}(V_N)$. *We consider a UV* $\bar{X}$ *representing a one-dimensional codebook, a corresponding output UV* $\bar{Y}$, *and* $\bar{\delta} = (2/6)^3$, *so that*

$$[\![\bar{X}]\!] = \{1, 7, 13\}, \tag{S115}$$

$$[\![\bar{Y}]\!] = \{1, 2, \ldots, 19\}, \tag{S116}$$

$$[\![\bar{Y}|\bar{X}]\!]^*_{1/81} = \{\mathscr{S}_1, \mathscr{S}_2, \mathscr{S}_3\}, \tag{S117}$$

where

$$\mathscr{S}_1 = \cup_{x \in \{1\}} N(x), \tag{S118}$$

$$\mathscr{S}_2 = \cup_{x \in \{7\}} N(x), \tag{S119}$$

$$\mathscr{S}_3 = \cup_{x \in \{13\}} N(x). \tag{S120}$$

Since $\bar{\delta} = \delta_1 = (2/6)^3$, *we have*

$$\hat{\delta} = \max_{\mathscr{S} \in [\![\bar{Y}|\bar{X}]\!]^*_{\bar{\delta}/|[\![\bar{X}]\!]|}} \frac{m_{\mathscr{Y}}(\mathscr{S})}{m_{\mathscr{Y}}([\![\bar{Y}]\!])} = \left(\frac{7}{19}\right)^3. \tag{S121}$$

It follows that for all $n > 1$, *we have* $\delta_n \geq \bar{\delta}(\hat{\delta}|[\![\bar{X}]\!]|)^{n-1}$ *and all the sufficient conditions in Theorem S3 are satisfied, so that*

$$C_N(\{\delta_n\})_* = \log_2 |[\![\bar{Y}|\bar{X}]\!]^*_{1/81}| = \log_2(3). \tag{S122}$$

Example S4. *We again consider the same channel and the same uncertainty function as in Example S3, shown in Figure S1 and* (S112), *respectively. We compute the capacity* $C_N(\{\downarrow 0\})^*$. *Consider an UV* X^* *representing a one-dimensional codebook such that*

$$[\![X^*]\!] = \{1, 7, 13\}. \tag{S123}$$

It follows that the corresponding output UV Y^* *is such that*

$$[\![Y^*]\!] = \{1, 2, 3 \ldots 19\}. \tag{S124}$$

Letting $\delta^* = (2/6)^3$, *we hold that* $\delta^*/|[\![X^*]\!]| = 1/81$ *and the overlap family is*

$$[\![Y^*|X^*]\!]^*_{\delta^*/|[\![X^*]\!]|=1/81} = \{\mathscr{S}_1, \mathscr{S}_2, \mathscr{S}_3\}, \tag{S125}$$

where

$$\mathscr{S}_1 = \cup_{x \in \{1\}} N(x), \tag{S126}$$

$$\mathscr{S}_2 = \cup_{x \in \{7\}} N(x), \tag{S127}$$

$$\mathscr{S}_3 = \cup_{x \in \{13\}} N(x). \tag{S128}$$

Since V_N *contains seven elements, we hold that* $m_{\mathscr{Y}}(V_N) = (7/19)^3$, *and* $\delta^* < m_{\mathscr{Y}}(V_N)$. *Also, we hold that*

$$\hat{\delta}_* = \max_{\mathscr{S} \in [\![Y^*|X^*]\!]^*_{\delta^*/|[\![X^*]\!]|}} \frac{m_{\mathscr{Y}}(\mathscr{S})}{m_{\mathscr{Y}}([\![Y^*]\!])} = \left(\frac{7}{19}\right)^3. \tag{S129}$$

It follows that $\hat{\delta}_*|[\![X^*]\!]| < 1$.

Since all the sufficient conditions in Theorem S4 are satisfied, we have

$$C_N(\{\downarrow 0\})^* \geq \log_2 |[\![\bar{Y}|\bar{X}]\!]^*_{2/18}| = \log_2(3). \tag{S130}$$

S3.1. Discussion

The results in our examples show that for the channel presented in Figure S1 and the uncertainty function (S96), there exists a vanishing sequence $\delta_1 = 2/9, \{\delta_n\}_2^\infty = \{(7/342)^n\}$ such that

$$C_N^{0*} = C_N(\{\delta_n\})^*. \tag{S131}$$

For the same channel and uncertainty function, there is another vanishing sequence, $\delta_1 = 4/9, \{\delta_n\}_2^\infty = \{(14/342)^n\}$, such that

$$C_N^{0*} < C_N(\{\delta_n\})^*. \tag{S132}$$

On the other hand, for the same channel using the uncertainty function (S112), there exists a vanishing sequence $\delta_1 = (2/6)^3, \{\delta_n\}_2^\infty = \{(2/6)^3(3(7/19)^3)^{n-1}\}$ such that

$$C_N(\{\delta_n\})_* \leq C_N(\{\downarrow 0\})^*. \tag{S133}$$

S4. Proof of Lemma S1

Proof. Let us begin with part 1). We have

$$\begin{aligned} &[\![Y(1:n)]\!] \\ &= \cup_{x(1:n)\in[\![X(1:n)]\!]}[\![Y(1:n)|x(1:n)]\!], \\ &\overset{(a)}{=} \cup_{x(1:n)\in[\![X(1:n)]\!]}[\![Y(1)|x(1)]\!] \times \ldots [\![Y(n)|x(n)]\!], \\ &\overset{(b)}{=} \cup_{x(1)\in[\![X(1)]\!]}[\![Y(1)|x(1)]\!]\times \\ &\quad \ldots \times \cup_{x(n)\in[\![X(n)]\!]}[\![Y(n)|x(n)]\!], \\ &= [\![Y(1)]\!] \times [\![Y(2)]\!] \times \ldots \times [\![Y(n)]\!], \end{aligned} \tag{S134}$$

where (a) follows from (S5) and (b) follows from (S4). Now, we have

$$\begin{aligned} \bigcup_{\mathscr{S}\in\prod_{i=1}^n[\![Y(i)|X(i)]\!]^*_\delta} \mathscr{S} &\overset{(a)}{=} \prod_{i=1}^n \Big(\bigcup_{\mathscr{S}\in[\![Y(i)|X(i)]\!]^*_\delta} \mathscr{S} \Big), \\ &\overset{(b)}{=} \prod_{i=1}^n [\![Y(i)]\!], \\ &\overset{(c)}{=} [\![Y(1:n)]\!], \end{aligned} \tag{S135}$$

where (a) follows from the fact that the cartesian product is distributive over union, namely

$$\cup_{(i,j)\in I\times J} A_i \times B_j = (\cup_{i\in I} A_i) \times (\cup_{j\in J} B_j), \tag{S136}$$

(b) follows from the fact that for all $1 \leq i \leq n$, $[\![Y(i)|X(i)]\!]^*_\delta$ is a covering of $[\![Y(i)]\!]$ by [Definition 6 MainDoc], and (c) follows from (S134). Hence, part 1) follows.

Now, we prove part 2). Here, we will first show that for all $\mathscr{S} \in \prod_{i=1}^n [\![Y(i)|X(i)]\!]^*_\delta$, we hold that $\mathscr{S}$ is δ^n-connected. Second, we will show that $\mathscr{S}$ contains at least one singly δ^n-connected set.

Let us begin with the first step of part 2). Consider a set $\mathscr{S} \in \prod_{i=1}^{n} [\![Y(i)|X(i)]\!]^*_\delta$. Then, there exists a sequence $\{\mathscr{S}_i\}_{i=1}^{n}$ such that

$$\mathscr{S} = \mathscr{S}_1 \times \mathscr{S}_2 \times \ldots \times \mathscr{S}_n, \tag{S137}$$

and for all $1 \leq i \leq n$,

$$\mathscr{S}_i \in [\![Y(i)|X(i)]\!]^*_\delta. \tag{S138}$$

Now, consider two points $y_1(1:n), y_2(1:n) \in \mathscr{S}$. Then, using (S5), (S134) and (S137), for all $1 \leq i \leq n$, we hold that

$$y_1(i), y_2(i) \in \mathscr{S}_i. \tag{S139}$$

Also, since $\mathscr{S}_i$ is δ-connected using (S138) and Property 1 of [Definition 6 MainDoc], we have

$$y_1(i) \overset{\delta}{\leftrightsquigarrow} y_2(i), \tag{S140}$$

namely there exists a sequence $\{[\![Y(i)|x_k(i)]\!]\}_{k=1}^{N(i)}$ such that

$$y_1(i) \in [\![Y(i)|x_1(i)]\!], y_2(i) \in [\![Y(i)|x_{N(i)}(i)]\!], \tag{S141}$$

and for all $1 \leq k < N(i)$,

$$\frac{m_{\mathscr{Y}}([\![Y(i)|x_k(i)]\!] \cap [\![Y(i)|x_{k+1}(i)]\!])}{m_{\mathscr{Y}}([\![Y(i)]\!])} > \delta. \tag{S142}$$

Without loss of generality, let

$$N(1) \leq N(2) \leq \ldots \leq N(n). \tag{S143}$$

Now, consider the following sequence of conditional ranges

$$\begin{aligned}
&[\![Y(1:n)|x_1(1), x_1(2)\ldots x_1(n)]\!],\\
&[\![Y(1:n)|x_2(1), x_2(2)\ldots x_2(n)]\!],\\
&\ldots\\
&[\![Y(1:n)|x_{N(1)}(1), x_{N(1)}(2)\ldots x_{N(1)}(n)]\!],\\
&[\![Y(1:n)|x_{N(1)}(1), x_{N(1)+1}(2)\ldots x_{N(1)+1}(n)]\!],\\
&\ldots\\
&[\![Y(1:n)|x_{N(1)}(1), x_{N(2)}(2)\ldots x_{N(n)}(n)]\!].
\end{aligned} \tag{S144}$$

In this sequence, for all $1 \leq k < N(n)$, if $x_k(i) = x_{k+1}(i)$, then we have

$$\begin{aligned}
&\frac{m_{\mathscr{Y}}([\![Y(i)|x_k(i)]\!] \cap [\![Y(i)|x_{k+1}(i)]\!])}{m_{\mathscr{Y}}([\![Y(i)]\!])}\\
&\overset{(a)}{=} \frac{m_{\mathscr{Y}}([\![Y(i)|x_k(i)]\!])}{m_{\mathscr{Y}}([\![Y(i)]\!])},\\
&\overset{(b)}{>} \frac{\delta}{m_{\mathscr{Y}}([\![Y(i)]\!])},\\
&\overset{(c)}{\geq} \frac{\delta}{m_{\mathscr{Y}}(\mathscr{Y})},\\
&\overset{(d)}{>} \delta,
\end{aligned} \tag{S145}$$

where (a) follows from the fact that $x_k(i) = x_{k+1}(i)$, (b) follows from (S6), (c) follows from the fact that $[\![Y(i)]\!] \subseteq \mathscr{Y}$ and [(10) MainDoc] holds, and (d) follows from the fact that $m_{\mathscr{Y}}(\mathscr{Y}) = 1$. Additionally, in the sequence (S144), for all $1 \le k < N(n)$, if $x_k(i) \ne x_{k+1}(i)$, then we have that (S142) holds. This along with (S145) implies that for all $1 \le k < N(n)$, we have

$$\frac{m_{\mathscr{Y}}([\![Y(i)|x_k(i)]\!] \cap [\![Y(i)|x_{k+1}(i)]\!])}{m_{\mathscr{Y}}([\![Y(i)]\!])} > \delta. \tag{S146}$$

Now, using (S5) and (S141), we have

$$y_1(1:n) \in [\![Y(1:n)|x_1(1), \dots x_1(n)]\!], \tag{S147}$$

and

$$y_2(1:n) \in [\![Y(1:n)|x_{N(1)}(1), \dots x_{N(n)}(n)]\!]. \tag{S148}$$

Also, for all $1 \le k < N(n)$, the uncertainty associated with the intersection of the two consecutive conditional ranges in the sequence (S144) is

$$\begin{aligned} &\frac{m_{\mathscr{Y}}([\![Y(1:n)|x_k(1:n)]\!] \cap [\![Y(1:n)|x_{k+1}(1:n)]\!])}{m_{\mathscr{Y}}([\![Y(1:n)]\!])}, \\ &\overset{(a)}{=} \prod_{i=1}^{n} \frac{m_{\mathscr{Y}}([\![Y(i)|x_k(i)]\!] \cap [\![Y(i)|x_{k+1}(i)]\!])}{m_{\mathscr{Y}}([\![Y(i)]\!])}, \\ &\overset{(b)}{>} \delta^n, \end{aligned} \tag{S149}$$

where (a) follows from Assumption S1, (S5), (S134) and the fact that

$$\prod_{i=1}^{n} \mathscr{S}_i \cap \prod_{i=1}^{n} \mathscr{T}_i = (\mathscr{S}_1 \cap \mathscr{T}_1) \times \dots \times (\mathscr{S}_n \cap \mathscr{T}_n), \tag{S150}$$

(b) follows from (S146). Hence, using (S147), (S148) and (S149), we have

$$y_1(1:n) \overset{\delta^n}{\leftrightsquigarrow} y_2(1:n). \tag{S151}$$

Hence, $\mathscr{S}$ is δ^n-connected.

Now, let us prove the second step of part 2). For all $1 \le i \le n$, since $[\![Y(i)|X(i)]\!]^*_\delta$ satisfies Property 1 of [Definition 6 MainDoc], there exists an $x_i \in [\![X(i)]\!]$ such that

$$[\![Y(i)|x_i]\!] \subseteq \mathscr{S}_i. \tag{S152}$$

Therefore, for $x(1:n) = [x_1, x_2, \dots, x_n]$, we have

$$\begin{aligned} &[\![Y(1:n)|x(1:n)]\!] \\ &\overset{(a)}{=} [\![Y(1)|x(1)]\!] \times \dots \times [\![Y(n)|x(n)]\!], \\ &\overset{(b)}{=} [\![Y(1)|x_1]\!] \times \dots \times [\![Y(n)|x_n]\!], \\ &\overset{(c)}{\subseteq} \mathscr{S}_1 \times \mathscr{S}_2 \times \dots \mathscr{S}_n, \\ &\overset{(d)}{=} \mathscr{S}, \end{aligned} \tag{S153}$$

where (a) follows from (S5), (b) follows from the fact that $x(1:n) = [x_1, x_2, \dots, x_n]$, (c) follows from (S152), and (d) follows from (S137). Hence, $\mathscr{S}$ contains at least one singly δ^n-connected set, which concludes the second step of part 2).

Now, let us prove part 3). For all $1 \leq i \leq n$, since $[\![Y(i)|X(i)]\!]^*_\delta$ satisfies Property 3 of [Definition 6 Main Doc], for all $x(i) \in [\![X(i)]\!]$, there exists a set $\mathscr{S}(x(i)) \in [\![Y(i)|X(i)]\!]^*_\delta$ such that

$$[\![Y(i)|x(i)]\!] \subseteq \mathscr{S}(x(i)). \tag{S154}$$

Then for all $x(1:n) \in [\![X(1:n)]\!]$, we have

$$\begin{aligned} &[\![Y(1:n)|x(1:n)]\!] \\ &\overset{(a)}{=} [\![Y(1)|x(1)]\!] \times \ldots \times [\![Y(n)|x(n)]\!], \\ &\overset{(b)}{\subseteq} \mathscr{S}(x(1)) \times \ldots \times \mathscr{S}(x(n)) \\ &\in \prod_{i=1}^{n} [\![Y(i)|X(i)]\!]^*_\delta, \end{aligned} \tag{S155}$$

where (a) follows from (S5), and (b) follows from (S154). Hence, part 3) follows.

Finally, let us prove part 4). Consider two distinct sets $\mathscr{S}_1, \mathscr{S}_2 \in \prod_{i=1}^{n} [\![Y(i)|X(i)]\!]^*_\delta$. Then, we have

$$\mathscr{S}_1 = \mathscr{S}_{11} \times \mathscr{S}_{12} \times \ldots \mathscr{S}_{1n}, \tag{S156}$$

$$\mathscr{S}_2 = \mathscr{S}_{21} \times \mathscr{S}_{22} \times \ldots \mathscr{S}_{2n}, \tag{S157}$$

where for all $1 \leq i \leq n$

$$\mathscr{S}_{1i}, \mathscr{S}_{2i} \in [\![Y(i)|X(i)]\!]^*_\delta. \tag{S158}$$

Since $\mathscr{S}_1 \neq \mathscr{S}_2$, there exists $1 \leq i^* \leq n$ such that

$$\mathscr{S}_{1i^*} \neq \mathscr{S}_{2i^*}. \tag{S159}$$

Then, by Property 2 of [Definition 6 MainDoc] and (S158), we have

$$\frac{m_{\mathscr{Y}}(\mathscr{S}_{1i^*} \cap \mathscr{S}_{2i^*})}{m_{\mathscr{Y}}([\![Y(i^*)]\!])} \leq \delta. \tag{S160}$$

Also, using (S10), we hold that for all $1 \leq i \leq n$,

$$\frac{m_{\mathscr{Y}}(\mathscr{S}_{1i})}{m_{\mathscr{Y}}([\![Y(i)]\!])} \leq \hat{\delta}(n). \tag{S161}$$

Then, we have

$$\begin{aligned} &\frac{m_{\mathscr{Y}}(\mathscr{S}_1 \cap \mathscr{S}_2)}{m_{\mathscr{Y}}([\![Y(1:n)]\!])} \\ &\overset{(a)}{=} \frac{m_{\mathscr{Y}}((\mathscr{S}_{11} \times \ldots \mathscr{S}_{1n}) \cap (\mathscr{S}_{21} \times \ldots \mathscr{S}_{2n}))}{m_{\mathscr{Y}}([\![Y(1:n)]\!])}, \\ &\overset{(b)}{=} \frac{m_{\mathscr{Y}}((\mathscr{S}_{11} \cap \mathscr{S}_{21}) \times \ldots \times (\mathscr{S}_{1n} \cap \mathscr{S}_{2n}))}{m_{\mathscr{Y}}([\![Y(1:n)]\!])}, \\ &\overset{(c)}{=} \frac{m_{\mathscr{Y}}(\mathscr{S}_{11} \cap \mathscr{S}_{21}) \ldots m_{\mathscr{Y}}(\mathscr{S}_{1n} \cap \mathscr{S}_{2n})}{\prod_{i=1}^{n} m_{\mathscr{Y}}([\![Y(i)]\!])}, \\ &\overset{(d)}{\leq} \frac{m_{\mathscr{Y}}(\mathscr{S}_{1i^*} \cap \mathscr{S}_{2i^*})}{m_{\mathscr{Y}}([\![Y(i^*)]\!])} \prod_{i \neq i^*} \frac{m_{\mathscr{Y}}(\mathscr{S}_{1i})}{m_{\mathscr{Y}}([\![Y(i)]\!])} \\ &\overset{(e)}{\leq} \delta(\hat{\delta}(n))^{n-1}, \end{aligned} \tag{S162}$$

where (a) follows from (S156) and (S157), (b) follows from the fact that for all sequences of sets $\{\mathscr{S}_i\}_{i=1}^n$ and $\{\mathscr{T}_i\}_{i=1}^n$, we have

$$\prod_{i=1}^{n} \mathscr{S}_i \cap \prod_{i=1}^{n} \mathscr{T}_i = (\mathscr{S}_1 \cap \mathscr{T}_1) \times \ldots \times (\mathscr{S}_n \cap \mathscr{T}_n), \tag{S163}$$

(c) follows from Assumption S1 and (S134), (d) follows from [(10) MainDoc] and the fact that for all $1 \leq i \leq n$, $\mathscr{S}_{1i} \cap \mathscr{S}_{2i} \subseteq \mathscr{S}_{1i}$, and (e) follows from (S160) and (S161). Hence, part 4) follows. □

S5. Auxiliary result

Lemma S2. *Consider two UVs X and Y. Let*

$$\delta^* = \frac{\min_{y \in [\![Y]\!]} m_{\mathscr{X}}([\![X|y]\!])}{m_{\mathscr{X}}([\![X]\!])}. \tag{S164}$$

For all $\delta_1 < \delta^*$ and $\delta_2 \leq 1$, if $(X, Y) \overset{a}{\leftrightarrow} (\delta_1/|[\![Y]\!]|, \delta_2)$, then under Assumption S2, for all $[\![X|y]\!] \in [\![X|Y]\!]$, there exists a point $x \in [\![X|y]\!]$ such that for all $[\![X|y']\!] \in [\![X|Y]\!] \setminus \{[\![X|y]\!]\}$,

$$x \notin [\![X|y']\!]. \tag{S165}$$

Proof. We will prove this by contradiction. Consider a set $[\![X|y]\!]$. Let x satisfying (S165) do not exist. Then, for all $x' \in [\![X|y]\!]$, there exists a set $[\![X|y']\!] \in [\![X|Y]\!] \setminus \{[\![X|y]\!]\}$ such that

$$x' \in [\![X|y']\!]. \tag{S166}$$

Thus, we have

$$\begin{aligned} & m_{\mathscr{X}}(\cup_{[\![X|y']\!] \in [\![X|Y]\!] \setminus \{[\![X|y]\!]\}}([\![X|y]\!] \cap [\![X|y']\!])), \\ & \overset{(a)}{\geq} m_{\mathscr{X}}([\![X|y]\!]), \\ & \overset{(b)}{\geq} \delta^* m_{\mathscr{X}}([\![X]\!]), \\ & \overset{(c)}{>} \delta_1 m_{\mathscr{X}}([\![X]\!]), \end{aligned} \tag{S167}$$

where (a) follows from (S166), (b) follows from (S164), and (c) follows from the fact that $\delta_1 < \delta^*$. On the other hand, since $(X, Y) \overset{a}{\leftrightarrow} (\delta_1/|[\![Y]\!]|, \delta_2)$, we have

$$\begin{aligned} & m_{\mathscr{X}}(\cup_{[\![X|y']\!] \in [\![X|Y]\!] \setminus \{[\![X|y]\!]\}}([\![X|y]\!] \cap [\![X|y']\!])), \\ & \overset{(a)}{\leq} \sum_{[\![X|y']\!] \in [\![X|Y]\!] \setminus \{[\![X|y]\!]\}} m_{\mathscr{X}}([\![X|y]\!] \cap [\![X|y']\!]), \\ & \overset{(b)}{\leq} |[\![Y]\!]| \delta_1 m_{\mathscr{X}}([\![X]\!]) / |[\![Y]\!]|, \\ & = \delta_1 m_{\mathscr{X}}([\![X]\!]), \end{aligned} \tag{S168}$$

where (a) follows from Assumption S2, (b) follows from [Lemma 1 Main Doc]. It follows that (S167) and (S168) contradict each other, and therefore x satisfying (S165) exists. The statement of the lemma follows. □

S6. Proof of 4 claims in Theorem S2

Proof of Claim 1. By Property 1 of [Definition 6 Main Doc], we hold that for all $\mathscr{S}_i \in [\![Y(1:n)|X(1:n)]\!]^*_{\delta'/|[\![X(1:n)]\!]|}$, there exists a $\tilde{x}_i(1:n) \in [\![X(1:n)]\!]$ such that $[\![Y(1:n)|\tilde{x}_i(1:n)]\!] \subseteq$

$\mathscr{S}_i$. Now, consider a new UV $\tilde{X}(1:n)$ whose marginal range is composed of elements of $[\![X(1:n)]\!]$, namely

$$[\![\tilde{X}(1:n)]\!] = \{\tilde{x}_1(1:n), \dots \tilde{x}_K(1:n)\}, \tag{S169}$$

where

$$K = |[\![Y(1:n)|X(1:n)]\!]^*_{\delta'/|[\![X(1:n)]\!]|}|. \tag{S170}$$

Let $\tilde{Y}(1:n)$ be the UV corresponding to the received variable. Then, similar to [(92) MainDoc], by Property 2 of [Definition 6 MainDoc] and since N is stationary memoryless channel, for all $x(1:n), x'(1:n) \in [\![\tilde{X}(1:n)]\!]$, we have

$$\begin{aligned} &\frac{m_{\mathscr{Y}}([\![\tilde{Y}(1:n)|x(1:n)]\!] \cap [\![\tilde{Y}(1:n)|x'(1:n)]\!])}{m_{\mathscr{Y}}([\![Y(1:n)]\!])} \\ &\le \frac{\delta'}{|[\![X(1:n)]\!]|}, \\ &\overset{(a)}{\le} \frac{\delta'}{|[\![\tilde{X}(1:n)]\!]|}, \end{aligned} \tag{S171}$$

where (a) follows from the fact that $[\![\tilde{X}(1:n)]\!] \subseteq [\![X(1:n)]\!]$. Similar to [(93) Main Doc], for all $x(1:n), x'(1:n) \in [\![\tilde{X}(1:n)]\!]$, we hold that

$$\begin{aligned} &\frac{m_{\mathscr{Y}}([\![\tilde{Y}(1:n)|x(1:n)]\!] \cap [\![\tilde{Y}(1:n)|x'(1:n)]\!])}{m_{\mathscr{Y}}([\![\tilde{Y}(1:n)]\!])} \\ &\le \frac{\delta' m_{\mathscr{Y}}([\![Y(1:n)]\!])}{|[\![\tilde{X}(1:n)]\!]| m_{\mathscr{Y}}([\![\tilde{Y}(1:n)]\!])}, \\ &= \frac{\tilde{\delta}}{|[\![\tilde{X}(1:n)]\!]|}, \end{aligned} \tag{S172}$$

where

$$\tilde{\delta} = \frac{\delta' m_{\mathscr{Y}}([\![Y(1:n)]\!])}{m_{\mathscr{Y}}([\![\tilde{Y}(1:n)]\!])}. \tag{S173}$$

Then, by [Lemma 1 MainDoc] it follows that

$$(\tilde{X}(1:n), \tilde{Y}(1:n)) \overset{a}{\leftrightarrow} (1, \tilde{\delta}/|[\![\tilde{X}(1:n)]\!]|). \tag{S174}$$

Using (S51), we also have

$$\tilde{\delta} \le \frac{\delta_n}{m_{\mathscr{Y}}([\![\tilde{Y}(1:n)]\!])}. \tag{S175}$$

Additionally, we have

$$\begin{aligned} \tilde{\delta} &\le \frac{\delta_n}{m_{\mathscr{Y}}([\![\tilde{Y}(1:n)]\!])} \\ &\overset{(a)}{\le} \left(\frac{\bar{\delta} m_{\mathscr{Y}}(V_N)}{|[\![\bar{X}]\!]|}\right)^n \frac{1}{m_{\mathscr{Y}}([\![\tilde{Y}(1:n)]\!])} \\ &\overset{(b)}{\le} \frac{(\bar{\delta} m_{\mathscr{Y}}(V_N))^n}{m_{\mathscr{Y}}([\![\tilde{Y}(1:n)]\!])}, \\ &\overset{(c)}{<} \frac{(m_{\mathscr{Y}}(V_N))^n}{m_{\mathscr{Y}}([\![\tilde{Y}(1:n)]\!])}, \\ &\overset{(d)}{=} \frac{m_{\mathscr{Y}}(V_N^n)}{m_{\mathscr{Y}}([\![\tilde{Y}(1:n)]\!])}, \end{aligned} \tag{S176}$$

where (a) follows from the assumption in the theorem that

$$0 \leq \delta_n \leq (\bar{\delta} m_{\mathscr{Y}}(V_N)/|[\![\bar{X}]\!]|)^n, \tag{S177}$$

(b) follows from the fact that $|[\![\bar{X}]\!]| \geq 1$, (c) follows from the fact that using $\delta_1 < m_{\mathscr{Y}}(V_N)$, we have

$$\bar{\delta} \leq \frac{\delta_1}{m_{\mathscr{Y}}([\![\bar{Y}]\!])} < \frac{m_{\mathscr{Y}}(V_N)}{m_{\mathscr{Y}}([\![\bar{Y}]\!])} \leq 1, \tag{S178}$$

and $d)$ follows from Assumption S1. Now, we have

$$\begin{aligned} &|[\![\tilde{Y}(1:n)|\tilde{X}(1:n)]\!]^*_{\bar{\delta}/|[\![\tilde{X}(1:n)]\!]|}| \\ &\stackrel{(a)}{=} |[\![\tilde{X}(1:n)]\!]| \\ &\stackrel{(b)}{=} |[\![Y(1:n)|X(1:n)]\!]^*_{\bar{\delta}/|[\![X(1:n)]\!]|}|, \end{aligned} \tag{S179}$$

where (a) follows by combining (S174), (S176) and [Lemma 5 MainDoc] and (b) follows from (S169) and (S170). This along with (S52) implies that we have

$$\begin{aligned} &|[\![\tilde{Y}(1:n)|\tilde{X}(1:n)]\!]^*_{\bar{\delta}/|[\![\tilde{X}(1:n)]\!]|}| \\ &> |\prod_{i=1}^{n} [\![\bar{Y}|\bar{X}]\!]^*_{\bar{\delta}/|[\![\bar{X}]\!]|}|. \end{aligned} \tag{S180}$$

This concludes the proof of Claim 1. □

Proof of Claim 2. Since (S174) and (S176) holds, using [Lemma 5 MainDoc], we have

$$\begin{aligned} &[\![\tilde{Y}(1:n)|\tilde{X}(1:n)]\!]^*_{\bar{\delta}/|[\![\tilde{X}(1:n)]\!]|} \\ &= [\![\tilde{Y}(1:n)|\tilde{X}(1:n)]\!]. \end{aligned} \tag{S181}$$

Using (S181) and Property 1 of [Definition 6 MainDoc] we hold that for all $\mathscr{S} \in [\![\tilde{Y}(1:n)|\tilde{X}(1:n)]\!]^*_{\bar{\delta}/|[\![\tilde{X}(1:n)]\!]|}$, there exists a $\tilde{x}(1:n) \in [\![\tilde{X}(1:n)]\!]$ such that

$$\mathscr{S} = [\![\tilde{Y}(1:n)|\tilde{x}(1:n)]\!]. \tag{S182}$$

Now, for all $x \in \mathscr{X}$, let $\mathscr{S}(x) \in [\![\bar{Y}|\bar{X}]\!]^*_{\bar{\delta}/|[\![\bar{X}]\!]|}$ be such that

$$[\![\bar{Y}|x]\!] \subseteq \mathscr{S}(x). \tag{S183}$$

For all $x \in \mathscr{X} \setminus [\![\bar{X}]\!]$, the set $\mathscr{S}(x)$ exists from the assumption in the theorem. Also, for all $x \in [\![\bar{X}]\!]$, the set $\mathscr{S}(x)$ exists using Property 3 in [Definition 6 Main Doc]. Hence, for all $x \in \mathscr{X}$, we hold that $\mathscr{S}(x)$ satisfying (S183) exists.

Hence, for all $\tilde{x}(1:n) \in [\![\tilde{X}(1:n)]\!]$, we hold that

$$\begin{aligned} &[\![\tilde{Y}(1:n)|\tilde{x}(1:n)]\!] \\ &\stackrel{(a)}{=} [\![\tilde{Y}(1)|\tilde{x}(1)]\!] \times \ldots \times [\![\tilde{Y}(n)|\tilde{x}(n)]\!], \\ &\stackrel{(b)}{\subseteq} \mathscr{S}(x(1)) \times \ldots \times \mathscr{S}(x(n)), \\ &\stackrel{(c)}{\in} \prod_{i=1}^{n} [\![\bar{Y}|\bar{X}]\!]^*_{\bar{\delta}/|[\![\bar{X}]\!]|}, \end{aligned} \tag{S184}$$

where (a) follows from the fact that N is a stationary, memoryless, uncertain channel, (b) follows from the fact that for all $x \in \mathscr{X}$, $\mathscr{S}(x)$ exists, and (c) follows from the fact that for all $x \in \mathscr{X}$, $\mathscr{S}(x) \in [\![\bar{Y}|\bar{X}]\!]^*_{\bar{\delta}/|[\![\bar{X}]\!]|}$. Hence, Claim 2 is proved. □

Proof of Claim 3. Combining (S180) and (S181), we hold that

$$|[\![\tilde{Y}(1:n)|\tilde{X}(1:n)]\!]| > \prod_{i=1}^{n}[\![\bar{Y}|\bar{X}]\!]^*_{\bar{\delta}/|[\![\bar{X}]\!]|}. \tag{S185}$$

This along with (S184) implies that there exists a set $\mathscr{S} \in \prod_{i=1}^{n}[\![\bar{Y}|\bar{X}]\!]^*_{\bar{\delta}/|[\![\bar{X}]\!]|}$ which contains at least two sets $\mathscr{D}_1, \mathscr{D}_2 \in [\![\tilde{Y}(1:n)|\tilde{X}(1:n)]\!]^*_{\tilde{\delta}/|[\![\tilde{X}(1:n)]\!]|}$, namely

$$\mathscr{D}_1 \subset \mathscr{S}, \tag{S186}$$

$$\mathscr{D}_2 \subset \mathscr{S}. \tag{S187}$$

Using (S182), without loss of generality, let

$$\mathscr{D}_1 = [\![\tilde{Y}(1:n)|\tilde{x}_1(1:n)]\!], \tag{S188}$$

$$\mathscr{D}_2 = [\![\tilde{Y}(1:n)|\tilde{x}_2(1:n)]\!]. \tag{S189}$$

Also, let

$$\mathscr{S} = \mathscr{S}_1 \times \ldots \times \mathscr{S}_n, \tag{S190}$$

where $\mathscr{S}_1, \ldots, \mathscr{S}_n \in [\![\bar{Y}|\bar{X}]\!]^*_{\bar{\delta}/|[\![\bar{X}]\!]|}$. Also, we have

$$\frac{\bar{\delta}}{|[\![\bar{X}]\!]|} \le \bar{\delta} \le \frac{\delta_1}{m_{\mathscr{Y}}([\![\bar{Y}]\!])} < \frac{m_{\mathscr{Y}}(V_N)}{m_{\mathscr{Y}}([\![\bar{Y}]\!])}. \tag{S191}$$

Now, we have

$$\begin{aligned}\frac{\tilde{\delta}}{|[\![\tilde{X}(1:n)]\!]|} &\le \frac{\delta_n}{m_{\mathscr{Y}}([\![\tilde{Y}(1:n)]\!])},\\ &\overset{(a)}{\le} \left(\frac{\bar{\delta} m_{\mathscr{Y}}(V_N)}{|[\![\bar{X}]\!]|}\right)^n \frac{1}{m_{\mathscr{Y}}([\![\tilde{Y}(1:n)]\!])},\\ &\overset{(b)}{\le} \left(\frac{\bar{\delta}}{|[\![\bar{X}]\!]|}\right)^n,\end{aligned} \tag{S192}$$

where (a) follows from the assumption in the theorem that

$$\delta_n \le \left(\frac{\bar{\delta} m_{\mathscr{Y}}(V_N)}{|[\![\bar{X}]\!]|}\right)^n, \tag{S193}$$

and (b) follows from the fact that using Assumption S1, we have

$$m_{\mathscr{Y}}(V_N^n) = (m_{\mathscr{Y}}(V_N))^n \le m_{\mathscr{Y}}([\![\tilde{Y}(1:n)]\!]). \tag{S194}$$

Combining [Lemma 1 Main Doc] and (S174), we have

$$\begin{aligned}&\frac{m_{\mathscr{Y}}(\llbracket \tilde{Y}(1:n)|\tilde{x}_1(1:n)\rrbracket \cap \llbracket \tilde{Y}(1:n)|\tilde{x}_2(1:n)\rrbracket)}{m_{\mathscr{Y}}(\llbracket \tilde{Y}(1:n)\rrbracket)}\\ &\leq \frac{\tilde{\delta}}{|\llbracket \tilde{X}(1:n)\rrbracket|}\\ &\overset{(a)}{\leq} \left(\frac{\bar{\delta}}{|\llbracket \bar{X}\rrbracket|}\right)^n,\end{aligned} \tag{S195}$$

where (a) follows from (S192). This implies that there exists a $1 \leq i^* \leq n$ such that

$$\frac{m_{\mathscr{Y}}(\llbracket \tilde{Y}(i^*)|\tilde{x}_1(i^*)\rrbracket \cap \llbracket \tilde{Y}(i^*)|\tilde{x}_2(i^*)\rrbracket)}{(m_{\mathscr{Y}}(\llbracket \tilde{Y}(1:n)\rrbracket))^{1/n}} \leq \frac{\bar{\delta}}{|\llbracket \bar{X}\rrbracket|}, \tag{S196}$$

otherwise (S195) does not hold, namely

$$\begin{aligned}&\frac{m_{\mathscr{Y}}(\llbracket \tilde{Y}(1:n)|\tilde{x}_1(1:n)\rrbracket \cap \llbracket \tilde{Y}(1:n)|\tilde{x}_2(1:n)\rrbracket)}{m_{\mathscr{Y}}(\llbracket \tilde{Y}(1:n)\rrbracket)}\\ &\overset{(a)}{=} \prod_{i=1}^{n}\left(\frac{m_{\mathscr{Y}}(\llbracket \tilde{Y}(i)|\tilde{x}_1(i)\rrbracket \cap \llbracket \tilde{Y}(i)|\tilde{x}_2(i)\rrbracket)}{(m_{\mathscr{Y}}(\llbracket \tilde{Y}(1:n)\rrbracket))^{1/n}}\right),\\ &\overset{(b)}{>} \left(\frac{\bar{\delta}}{|\llbracket \bar{X}\rrbracket|}\right)^n,\end{aligned} \tag{S197}$$

where (a) follows from Assumption S1 and the fact that N is stationary memoryless, (b) follows from the hypothesis that i^* satisfying (S196) does not exist. □

Proof of Claim 4. Now, consider a UV X' such that

$$\begin{aligned}\llbracket X'\rrbracket = (&\llbracket \bar{X}\rrbracket \setminus \{x \in \mathscr{X} : \llbracket Y|x\rrbracket \subseteq \mathscr{S}_{i^*}\})\\ &\cup \{\tilde{x}_1(i^*)\} \cup \{\tilde{x}_2(i^*)\}.\end{aligned} \tag{S198}$$

For $\llbracket X'_1\rrbracket = (\llbracket \bar{X}\rrbracket \setminus \{x \in \mathscr{X} : \llbracket Y|x\rrbracket \subseteq \mathscr{S}_{i^*}\})$, the δ'_1- overlap family of $\llbracket Y'_1|X'_1\rrbracket$ satisfies

$$|\llbracket \bar{Y}|\bar{X}\rrbracket^*_{\bar{\delta}/|\llbracket \bar{X}\rrbracket|}| - 1 \overset{(a)}{\leq} |\llbracket Y'_1|X'_1\rrbracket^*_{\delta'_1}|, \tag{S199}$$

where

$$\delta'_1 = (\bar{\delta} m_{\mathscr{Y}}(\llbracket \bar{Y}\rrbracket))/(|\llbracket \bar{X}\rrbracket| m_{\mathscr{Y}}(\llbracket Y'_1\rrbracket), \tag{S200}$$

and (a) follows from the fact that

$$\mathcal{S}_1 = \{\mathscr{S}' \in \llbracket \bar{Y}|\bar{X}\rrbracket^*_{\bar{\delta}/|\llbracket \bar{X}\rrbracket|} : \mathscr{S}' \neq \mathscr{S}_{i^*}\} \tag{S201}$$

satisfies all the properties of $\llbracket Y'_1|X'_1\rrbracket^*_{\delta'_1}$ in [Definition 6 Main Doc].

Now, consider the UV X' such that $\llbracket X'\rrbracket = \llbracket X'_1\rrbracket \cup \{\tilde{x}_1(i^*)\} \cup \{\tilde{x}_2(i^*)\}$. We will show that

$$\mathcal{S}_3 = \mathcal{S}_1 \cup \{\llbracket \tilde{Y}(i^*)|\tilde{x}_1(i^*)\rrbracket\} \cup \{\llbracket \tilde{Y}(i^*)|\tilde{x}_2(i^*)\rrbracket\}. \tag{S202}$$

satisfies the property of $\llbracket Y'|X'\rrbracket^*_{\delta^*/|\llbracket \bar{X}'\rrbracket|}$, where

$$\delta^* = \frac{\bar{\delta}|\llbracket X'\rrbracket| m_{\mathscr{Y}}(\llbracket \bar{Y}\rrbracket)}{|\llbracket \bar{X}\rrbracket| m_{\mathscr{Y}}(\llbracket Y'\rrbracket)}. \tag{S203}$$

Using (S186), (S187) and Claim 2, we have

$$[\![\tilde{Y}(i^*)|\tilde{x}_1(i^*)]\!], [\![\tilde{Y}(i^*)|\tilde{x}_2(i^*)]\!] \subseteq \mathscr{S}_{i^*}. \tag{S204}$$

This along with the fact that $[\![\bar{Y}|\bar{X}]\!]_{\bar{\delta}/|[\![\bar{X}]\!]|}$ is an overlap family implies that for all $\mathscr{S}' \in \mathcal{S}_1$,

$$m_{\mathscr{Y}}([\![\tilde{Y}(i^*)|\tilde{x}_1(i^*)]\!] \cap \mathscr{S}') \leq \frac{\bar{\delta} m_{\mathscr{Y}}([\![\bar{Y}]\!])}{|[\![\bar{X}]\!]|}, \tag{S205}$$

and

$$m_{\mathscr{Y}}([\![\tilde{Y}(i^*)|\tilde{x}_2(i^*)]\!] \cap \mathscr{S}') \leq \frac{\bar{\delta} m_{\mathscr{Y}}([\![\bar{Y}]\!])}{|[\![\bar{X}]\!]|}. \tag{S206}$$

Also, we hold that

$$\begin{aligned} & m_{\mathscr{Y}}([\![\tilde{Y}(i^*)|\tilde{x}_1(i^*)]\!] \cap [\![\tilde{Y}(i^*)|\tilde{x}_2(i^*)]\!]) \\ & \stackrel{(a)}{\leq} \frac{\bar{\delta}(m_{\mathscr{Y}}([\![\tilde{Y}(1:n)]\!]))^{1/n}}{|[\![\bar{X}]\!]|} \\ & \stackrel{(b)}{\leq} \frac{\bar{\delta}(m_{\mathscr{Y}}([\![\bar{Y}(1:n)]\!]))^{1/n}}{|[\![\bar{X}]\!]|} \\ & \stackrel{(c)}{=} \frac{\bar{\delta} m_{\mathscr{Y}}([\![\bar{Y}]\!])}{|[\![\bar{X}]\!]|}, \end{aligned} \tag{S207}$$

where (a) follows from (S196), (b) follows from [(10) Main Doc] and $[\![\tilde{Y}(1:n)]\!] \subseteq [\![Y(1:n)]\!]$ by Claim 2, and (c) follows from Assumption S1 and (S45). Additionally, $[\![\tilde{Y}(i^*)|\tilde{x}_1(i)]\!]$ and $[\![\tilde{Y}(i^*)|\tilde{x}_2(i)]\!]$ are singly $\delta^*/|[\![\bar{X}']\!]|$ connected sets. This along with (S205), (S206) and (S207) implies that $\mathcal{S}_3$ satisfies all the properties of $[\![Y'|X']\!]^*_{\delta^*/|[\![X']\!]|}$. It follows that

$$\begin{aligned} |[\![Y'|X']\!]^*_{\delta^*/|[\![X']\!]|}| &\geq |\mathcal{S}_3|, \\ &\stackrel{(a)}{=} |\mathcal{S}_1| + 2, \\ &\stackrel{(b)}{=} |[\![\bar{Y}|\bar{X}]\!]^*_{\bar{\delta}/|[\![\bar{X}]\!]|}| + 1, \end{aligned} \tag{S208}$$

where (a) follows from (S202), and (b) follows from (S201).

Now, we will show that

$$|[\![X']\!]| \leq |[\![\bar{X}]\!]| + 1. \tag{S209}$$

We split the analysis into two mutually exclusive cases: $\tilde{x}_1(i^*) \in [\![\bar{X}]\!]$ or $\tilde{x}_2(i^*) \in [\![\bar{X}]\!]$; and $\tilde{x}_1(i^*), \tilde{x}_2(i^*) \notin [\![\bar{X}]\!]$. In the first case, if $\tilde{x}_1(i^*) \in [\![\bar{X}]\!]$ or $\tilde{x}_2(i^*) \in [\![\bar{X}]\!]$, then using (S198), we have

$$|[\![X']\!]| \leq |[\![\bar{X}]\!]| + 1. \tag{S210}$$

In the second case, if $\tilde{x}_1(i^*), \tilde{x}_2(i^*) \notin [\![\bar{X}]\!]$, then using (S204), there exists a non-empty set $\mathscr{P} \subseteq [\![\bar{X}]\!]$ such that

$$[\![\tilde{Y}(i^*)|\tilde{x}_1(i^*)]\!] \cup [\![\tilde{Y}(i^*)|\tilde{x}_2(i^*)]\!] \subseteq \cup_{x \in \mathscr{P}} [\![\bar{Y}|x]\!]. \tag{S211}$$

Also, there exists a $x' \in \mathcal{P}$ such that

$$[\![\bar{Y}|x']\!] \subseteq \mathscr{S}_{i^*}. \tag{S212}$$

This can be proved by contradiction. Let $x' \in \mathcal{P}$ satisfying (S212) does not exist. We have

$$\begin{aligned} & m_{\mathscr{Y}}(\cup_{\mathscr{S}' \in [\![\bar{Y}|\bar{X}]\!]^*_{\bar{\delta}/|[\![\bar{X}]\!]|}: \mathscr{S}' \neq \mathscr{S}_{i^*}} (\mathscr{S}_{i^*} \cap \mathscr{S}')) \\ & \overset{(a)}{\geq} m_{\mathscr{Y}}(\cup_{x: x \in \mathcal{P}} (\mathscr{S}_{i^*} \cap [\![\bar{Y}|x]\!])), \\ & \overset{(b)}{\geq} m_{\mathscr{Y}}([\![\tilde{Y}(i^*)|\tilde{x}_1(i^*)]\!] \cup [\![\tilde{Y}(i^*)|\tilde{x}_2(i^*)]\!]), \\ & \overset{(c)}{\geq} m_{\mathscr{Y}}(V_N) \\ & \overset{(d)}{>} \delta_1 \\ & \overset{(e)}{\geq} \bar{\delta} m_{\mathscr{Y}}([\![\bar{Y}]\!]), \end{aligned} \tag{S213}$$

where (a) follows from the fact that combining $\mathcal{P} \subseteq [\![X]\!]$, Property 3 of Definition [Definition 6 MainDoc], and the hypothesis that x' does not exist, we have

$$\cup_{x \in \mathscr{P}} [\![\bar{Y}|x]\!] \subseteq \mathscr{S}' \in \cup_{[\![\bar{Y}|\bar{X}]\!]^*_{\bar{\delta}/|[\![\bar{X}]\!]|}: \mathscr{S}' \neq \mathscr{S}_{i^*}} \mathscr{S}', \tag{S214}$$

(b) follows from (S204) and (S211), (c) follows from [(10) MainDoc] and the fact that for all $x \in \mathscr{X}$,

$$m_{\mathscr{Y}}(V_N) \leq m_{\mathscr{Y}}([\![Y|x]\!]), \tag{S215}$$

(d) follows from the fact that $\delta_1 < m_{\mathscr{Y}}(V_N)$, and (e) follows from the fact that $\bar{\delta} \leq \delta_1 / m_{\mathscr{Y}}([\![\bar{Y}]\!])$. On the other hand, since $[\![\bar{Y}|\bar{X}]\!]^*_{\bar{\delta}/|[\![\bar{X}]\!]|}$ is an overlap family, we have

$$\begin{aligned} & m_{\mathscr{Y}}(\cup_{\mathscr{S}' \in [\![\bar{Y}|\bar{X}]\!]^*_{\bar{\delta}/|[\![\bar{X}]\!]|}: \mathscr{S}' \neq \mathscr{S}_{i^*}} (\mathscr{S}_{i^*} \cap \mathscr{S}')), \\ & \overset{(a)}{\leq} \sum_{\mathscr{S}' \in [\![\bar{Y}|\bar{X}]\!]^*_{\bar{\delta}/|[\![\bar{X}]\!]|}: \mathscr{S}' \neq \mathscr{S}_{i^*}} m_{\mathscr{Y}}(\mathscr{S}_{i^*} \cap \mathscr{S}'), \\ & \overset{(b)}{\leq} \frac{\bar{\delta} |[\![\bar{Y}|\bar{X}]\!]^*_{\bar{\delta}/|[\![\bar{X}]\!]|}| m_{\mathscr{Y}}([\![\bar{Y}]\!])}{|[\![\bar{X}]\!]|}, \\ & \overset{(c)}{\leq} \bar{\delta} m_{\mathscr{Y}}([\![\bar{Y}]\!]) \end{aligned} \tag{S216}$$

where (a) follows from Assumption S2, (b) follows from Property 2 of [Definition 6 MainDoc], and (c) follows from the fact that using (S191), [Lemma 4 Main Doc] holds. Hence, (S213) and (S216) contradict each other, which implies x' satisfying (S212) exists. Now, using (S212) and (S198) , we hold that

$$|[\![X']\!]| \leq |[\![\bar{X}]\!]| + 1. \tag{S217}$$

Hence, (S209) holds.

Finally, we have

$$\begin{aligned} \delta^* &= \frac{\bar{\delta} |[\![X']\!]| m_{\mathscr{Y}}([\![\bar{Y}]\!])}{|[\![\bar{X}]\!]| m_{\mathscr{Y}}([\![Y']\!])}, \\ & \overset{(a)}{\leq} \frac{\bar{\delta} m_{\mathscr{Y}}([\![\bar{Y}]\!])}{m_{\mathscr{Y}}([\![Y']\!])} \left(1 + \frac{1}{|[\![\bar{X}]\!]|}\right), \\ & \overset{(b)}{\leq} \frac{\delta_1}{m_{\mathscr{Y}}([\![Y']\!])}, \end{aligned} \tag{S218}$$

where (a) follows from (S209), and (b) follows from the assumption in the theorem that $\bar{\delta}(1 + 1/|[\![\bar{X}]\!]|) \leq \delta_1/m_{\mathscr{Y}}([\![\bar{Y}]\!])$. Now, using (S208) and (S218), we hold that there exists a $\delta^* \leq \delta_1/m_{\mathscr{Y}}([\![Y']\!])$ such that

$$|[\![Y'|X']\!]^*_{\delta^*/|[\![X']\!]|}| > |[\![\bar{Y}|\bar{X}]\!]^*_{\bar{\delta}/|[\![\bar{X}]\!]|}|. \tag{S219}$$

This concludes the proof of Claim 4. □
